# Local Well-Posedness of the Skew Mean Curvature Flow for Small Data in $d\geqq 2$ Dimensions

**DOI:** 10.1007/s00205-023-01952-y

**Published:** 2024-01-25

**Authors:** Jiaxi Huang, Daniel Tataru

**Affiliations:** 1 School of Mathematics and Statistics, Beijing Institute of Technology, Beijing, 100081 People’s Republic of China; 2 Department of Mathematics, University of California, Berkeley, Berkeley, CA 94720 USA

**Keywords:** Primary: 35Q55, Secondary: 53E10

## Abstract

The skew mean curvature flow is an evolution equation for *d* dimensional manifolds embedded in ${\mathbb {R}}^{d+2}$ (or more generally, in a Riemannian manifold). It can be viewed as a Schrödinger analogue of the mean curvature flow, or alternatively as a quasilinear version of the Schrödinger Map equation. In an earlier paper, the authors introduced a harmonic/Coulomb gauge formulation of the problem, and used it to prove small data local well-posedness in dimensions $d \geqq 4$. In this article, we prove small data local well-posedness in low-regularity Sobolev spaces for the skew mean curvature flow in dimension $d\geqq 2$. This is achieved by introducing a new, heat gauge formulation of the equations, which turns out to be more robust in low dimensions.

## Introduction

In this article we continue our study of the local well-posedness for the skew mean curvature flow (SMCF). This is a nonlinear Schrödinger type flow modeling the evolution of a *d* dimensional oriented manifold embedded into a fixed oriented $d+2$ dimensional manifold; it can be seen as a Schrödinger analogue of the well studied mean curvature flow.

In an earlier work [9], we considered the (SMCF) flow in higher dimension $d \geqq 4$, and proved local well-posedness for small initial data in low regularity Sobolev spaces. This was achieved by developing a suitable harmonic/Coulomb gauge formulation of the equations, which allowed us to reformulate the problem as a quasilinear Schrödinger evolution.

In this article, we consider the small data local well-posedness for the skew mean curvature flow in low dimensions $d \geqq 2$, also for low regularity initial data. As the earlier harmonic/Coulomb gauge formulation has issues in low dimensions, here we introduce an alternative heat gauge, which resolves these difficulties.

### The (SMCF) Equations

Let $\Sigma ^d$ be a *d*-dimensional oriented manifold, and $({\mathcal {N}}^{d+2},g_{{\mathcal {N}}})$ be a $d+2$-dimensional oriented Riemannian manifold. Let $I=[0,T]$ be an interval and $F:I\times \Sigma ^d \rightarrow {\mathcal {N}}$ be a one parameter family of immersions. This induces a time dependent Riemannian structure on $\Sigma ^d$. For each $t\in I$, we denote the submanifold by $\Sigma _t=F(t,\Sigma )$, its tangent bundle by $T\Sigma _t$, and its normal bundle by $N\Sigma _t$ respectively. For an arbitrary vector *Z* at *F* we denote by $Z^\perp$ its orthogonal projection onto $N\Sigma _t$. The mean curvature ${\textbf{H}}(F)$ of $\Sigma _t$ can be identified naturally with a section of the normal bundle $N\Sigma _t$.

The normal bundle $N\Sigma _t$ is a rank two vector bundle with a naturally induced complex structure *J*(*F*) which simply rotates a vector in the normal space by $\pi /2$ positively. Namely, for any point $y=F(t,x)\in \Sigma _t$ and any normal vector $\nu \in N_{y}\Sigma _t$, we define $J(F)\in N_{y}\Sigma _t$ as the unique vector with the same length so that

$$\begin{aligned} J(F)\nu \bot \nu , \qquad \omega (F_{*}(e_1), F_{*}(e_2),\cdots F_{*}(e_d), \nu , J(F)\nu )>0, \end{aligned}$$

where $\omega$ is the volume form of ${\mathcal {N}}$ and $\{e_1,\cdots ,e_d\}$ is an oriented basis of $\Sigma ^d$. The skew mean curvature flow (SMCF) is defined by the initial value problem

1.1 $$\begin{aligned} \left\{ \begin{aligned}&(\partial _t F)^{\perp }=J(F){\textbf{H}}(F),\\&F(\cdot ,0)=F_0, \end{aligned}\right. \end{aligned}$$

which evolves a codimension two submanifold along its binormal direction with a speed given by its mean curvature.

The (SMCF) was derived both in physics and mathematics. The one-dimensional (SMCF) in the Euclidean space ${{\mathbb {R}}}^3$ is the well-known vortex filament equation (VFE)

$$\begin{aligned} \partial _t \gamma =\partial _s \gamma \times \partial _s^2 \gamma , \end{aligned}$$

where $\gamma$ is a time-dependent space curve, *s* is its arc-length parameter and $\times$ denotes the cross product in ${{\mathbb {R}}}^3$. The (VFE) was first discovered by Da Rios [4] in 1906 in the study of the free motion of a vortex filament.

The (SMCF) also arises in the study of asymptotic dynamics of vortices in the context of superfluidity and superconductivity. For the Gross–Pitaevskii equation, which models the wave function associated with a Bose–Einstein condensate, physics evidence indicates that the vortices would evolve along the (SMCF). An incomplete verification was attempted by Lin [18] for the vortex filaments in three space dimensions. For higher dimensions, Jerrard [11] proved this conjecture when the initial singular set is a codimension two sphere with multiplicity one.

The other motivation is that the (SMCF) naturally arises in the study of the hydrodynamical Euler equation. A singular vortex in a fluid is called a vortex membrane in higher dimensions if it is supported on a codimension two subset. The law of locally induced motion of a vortex membrane can be deduced from the Euler equation by applying the Biot–Savart formula. Shashikanth [23] first investigated the motion of a vortex membrane in ${{\mathbb {R}}}^4$ and showed that it is governed by the two dimensional (SMCF), while Khesin [15] then generalized this conclusion to any dimensional vortex membranes in Euclidean spaces.

From a mathematical standpoint, the (SMCF) equation is a canonical geometric flow for codimension two submanifolds which can be viewed as the Schrödinger analogue of the well studied mean curvature flow. In fact, the infinite-dimensional space of codimension two immersions of a Riemannian manifold admits a generalized Marsden–Weinstein sympletic structure, and hence the Hamiltonian flow of the volume functional on this space is verified to be the (SMCF). Haller–Vizman [8] noted this fact where they studied the nonlinear Grassmannians. For a detailed mathematical derivation of these equations we refer the reader to the article [26, Section 2.1].

The one dimensional case of this problem has been extensively studied. This is because the one dimensional (SMCF) flow agrees the classical Schrödinger Map type equation, provided that one chooses suitable coordinates, i.e. the arclength parametrization. As such, it exhibits many special properties (e.g. complete integrability) which are absent in higher dimensions. For more details we refer the reader to the survey article of Vega [27] as well as [1] and [8].

The study of higher dimensional (SMCF), on the other hand, is far less developed. Song–Sun [26] proved the local existence of (SMCF) with a smooth, compact oriented surface as the initial data in two dimensions, then Song [25] generalized this result to compact oriented manifolds for all $d\geqq 2$ and also proved a corresponding uniqueness result. Song [24] also proved that the Gauss map of a *d* dimensional (SMCF) in ${{\mathbb {R}}}^{d+2}$ satisfies a Schrödinger Map type equation but relative to the varying metric. More recently, Li [16, 17] considered a class of transversal small pertubations of Euclidean planes under the (SMCF) and proved a global regularity result for small initial data.

This article is instead concerned with the case when $\Sigma ^d = {{\mathbb {R}}}^d$, i.e. where $\Sigma _t$ has a trivial topology. We will further restrict to the case when ${\mathcal {N}}^{d+2}$ is the Euclidean space ${{\mathbb {R}}}^{d+2}$. Thus, the reader should visualize $\Sigma _t$ as an asymptotically flat codimension two submanifold of ${{\mathbb {R}}}^{d+2}$.

Such manifolds $\Sigma ={{\mathbb {R}}}^d$ with $d\geqq 4$ were already considered in our earlier work [9], where we proved the local well-posedness for small data in low-regularity Sobolev spaces. Here we consider instead the lower dimensional case, namely the dimensions $d = 2,3$. A key role in both [9] and in this article was played by our gauge choices, which are discussed next.

### Gauge Choices for (SMCF)

There are two components for the gauge choice, which are briefly discussed here and in full detail in Section 2:

1. The choice of coordinates on $I \times \Sigma$.
2. The choice of an orthonormal frame on $I \times N\Sigma$.

Indeed, as written above in (1.1), the (SMCF) equations are independent of the choice of coordinates in $I \times \Sigma$; here we include the time interval *I* to emphasize that coordinates may be chosen in a time dependent fashion. The manifold $\Sigma ^d$ simply serves to provide a parametrization for the moving manifold $\Sigma _t$; it determines the topology of $\Sigma _t$, but nothing else. Thus, the (SMCF) system written in the form (1.1) should be seen as a geometric evolution, with a large gauge group, namely the group of time dependent changes of coordinates in $I \times \Sigma$. One may think of the gauge choice here as having two components, (i) the choice of coordinates at the initial time, and (ii) the time evolution of the coordinates. One way to describe the latter choice is to rewrite the equations in the form

$$\begin{aligned} \left\{ \begin{aligned}&(\partial _t - V \partial _x) F =J(F){\textbf{H}}(F),\\&F(\cdot ,0)=F_0, \end{aligned}\right. \end{aligned}$$

where the vector field *V* can be freely chosen, and captures the time evolution of the coordinates. Indeed, some of the earlier papers [26] and [25] on (SMCF) use this formulation with $V = 0$. This would seem to simplify the equations, however it introduces difficulties at the level of comparing solutions.This is because the regularity of the map *F* is no longer determined by the regularity of the second fundamental form, and instead there is a loss of derivatives which may only be avoided if the initial data is assumed to have extra regularity. This loss is what prevents a complete low regularity theory in that approach.

In our earlier work [9] in dimension $d \geqq 4$, we chose *harmonic coordinates* on $\Sigma$, separately at each time. This implicitly fixes *V*, which may be obtained as the solution of an appropriate elliptic equation. The same approach could be made to work in dimension $d = 3$, if one uses a more careful study of the linearized equation as in the present paper. Unfortunately this does not seem to work well in two dimensions, essentially due to a lack of sufficient control on the metric at low regularity, which is caused by a lack of decay of the fundamental solution for the Laplacian.

To rectify this issue, in the present paper we use instead a *heat gauge*, where the coordinates and implicitely the metric are determined dynamically via a heat flow. This in particular requires also a good choice of coordinates at the initial time; there, we fall back to the harmonic coordinates. In dimension three and higher, this is all that is needed, and in effect both gauge choices, i.e. the heat gauge and the harmonic gauge, work equally well. However, in two dimensions the harmonic coordinates fail to yield the needed low frequency decay of the metric. We rectify this by adding an a-priori low frequency assumption on the metric in suitable coordinates, and then propagate this in time via the heat gauge.

We now discuss the second component of the gauge choice, namely the orthonormal frame in the normal bundle. Such a choice is needed in order to fix the second fundamental form for $\Sigma$; indeed, the (SMCF) is most naturally interpreted as a nonlinear Schrödinger evolution for the second fundamental form of $\Sigma$. In our earlier paper [9] we use the Coulomb gauge. But that seems to no longer be well behaved in two dimensions, so we replace it again with a heat flow. In this context, this strategy is reminiscent of the work of the second author and collaborators for the Chern–Simons–Schrödinger flow in [20].

### Scaling and Function Spaces

To understand what are the natural thresholds for local well-posedness, it is interesting to to consider the scaling properties of the solutions. As one might expect, a clean scaling law is obtained when $\Sigma ^d = {{\mathbb {R}}}^d$ and ${\mathcal {N}}^{d+2} = {{\mathbb {R}}}^{d+2}$. Then we have the following:

#### Proposition 1.1

(Scale invariance for (SMCF)) Assume that *F* is a solution of (1.1) with initial data $F(0)=F_0$, then ${F}_\mu (t,x):=\mu ^{-1}F(\mu ^2 t,\mu x)$ is a solution of (1.1) with initial data ${F}_\mu (0)=\mu ^{-1}F_0(\mu x)$.

The above scaling would suggest the critical Sobolev space for our moving surfaces $\Sigma _t$ to be $\dot{H}^{\frac{d}{2}+1}$. However, instead of working directly with the surfaces, it is far more convenient to track the regularity at the level of the curvature ${\textbf{H}}(\Sigma _t)$, which scales at the level of $\dot{H}^{\frac{d}{2}-1}$.

For our main result we will use instead inhomogeneous Sobolev spaces, and it will suffice to go one derivative above scaling. There is also a low frequency issue, precisely in two space dimensions where the $L^2$ norm is critical. There we will need to make a slightly stronger assumption on the low frequency part of the initial data.

### The Main Result

Our objective in this paper is to establish the local well-posedness of skew mean curvature flow for small data at low regularity. A key observation is that providing a rigorous description of fractional Sobolev spaces for functions (tensors) on a rough manifold is a delicate matter, which a-priori requires both a good choice of coordinates on the manifold and a good frame on the vector bundle (the normal bundle in our case). This is done in the next section, where we fix the gauge and write the equation as a quasilinear Schrödinger evolution in a good gauge. At this point, we content ourselves with a less precise formulation of the main result.

#### Theorem 1.2

(Small data local well-posedness in dimensions $d\geqq 3$) Let $d\geqq 3$, $s>\frac{d}{2}$ and $\sigma _d=\frac{d}{2}-\delta$. Then there exists $\epsilon _0>0$ sufficiently small such that, for all initial data $\Sigma _0$ with metric $g_0$ and mean curvature ${\textbf{H}}_0$ satisfying

1.2 $$\begin{aligned} \Vert |D|^{\sigma _d} (g_0-I_d)\Vert _{H^{s+1-\sigma _d}}\leqq \epsilon _0, \qquad \Vert {\textbf{H}}_0 \Vert _{H^s(\Sigma _0)}\leqq \epsilon _0, \end{aligned}$$

relative to some parametrization of $\Sigma _0$, the skew mean curvature flow (1.1) for maps from ${{\mathbb {R}}}^d$ to the Euclidean space $({{\mathbb {R}}}^{d+2},g_{{{\mathbb {R}}}^{d+2}})$ is locally well-posed on the time interval $I=[0,1]$ in a suitable gauge.

With a slight adjustment, a similar result holds in dimension $d=2$.

#### Theorem 1.3

(Small data local well-posedness in dimension $d=2$) Let $d = 2$, $s>\frac{d}{2}$ and $\sigma _d=\frac{d}{2}-\delta$. Then there exists $\epsilon _0>0$ sufficiently small such that, for all initial data $\Sigma _0$ with metric $g_0$ and mean curvature ${\textbf{H}}_0$ satisfying

$$\begin{aligned} \Vert |D|^{\sigma _d} (g_0-I_d)\Vert _{H^{s+1-\sigma _d}}\leqq \epsilon _0, \qquad \Vert {\textbf{H}}_0 \Vert _{H^s(\Sigma _0)}\leqq \epsilon _0, \end{aligned}$$

as well as a low frequency bound for $g_0$

1.3 $$\begin{aligned} \Vert g_0-I_d\Vert _{Y_{0}^{lo}}<\epsilon _0, \end{aligned}$$

relative to some parametrization of $\Sigma _0$, the skew mean curvature flow (1.1) for maps from ${{\mathbb {R}}}^d$ to the Euclidean space $({{\mathbb {R}}}^{d+2},g_{{{\mathbb {R}}}^{d+2}})$ is locally well-posed on the time interval $I=[0,1]$ in a suitable gauge.

We continue with some comments on the function spaces in the above theorems:

- For the metric $g_0$, we use the difference $g_0-I_d$ in the above statements in order to emphasize the normalization $g_0 \rightarrow I_d$ at infinity.
- In dimension $d \geqq 3$, the $g_0-I_d$ norm in (1.2) only plays a qualitative role, namely to place us in a regime where, in harmonic coordinates, $g_0$ is uniquely determined by the mean curvature ${\textbf{H}}_0$.
- The $Y_0^{lo}$ norm in (1.3), defined in Section 3, captures low frequency ${l}^1$ summability properties for $g_0$ with respect to cube lattice partitions of ${{\mathbb {R}}}^d$. Similar norm appear in our analysis in dimensions $d \geqq 3$. The main difference is that in higher dimension, the *Y* norms of $g_0-I_d$ can be estimated in terms of the $H^s$ norm of ${\textbf{H}}$ in harmonic coordinates. In two dimensions, this estimate borderline fails, so we instead include the $Y_0^{lo}$ bound in the hypothesis.

Following the spirit of our earlier work [9], in these results we consider rough data and provide a full, Hadamard style well-posedness result based on a more modern, frequency envelope approach and using a paradifferential form for both the full and the linearized equations. For an overview of these ideas we refer the reader to the expository paper [10]. This is unlike any of the prior results, which prove only existence and uniqueness for smooth data.

The favourable gauge mentioned in the theorem is defined in the next section in two steps:

- at the initial time, where we proceed as in [9], and use
  Harmonic coordinates on the manifold $\Sigma _0$.
  The Coulomb gauge for the orthonormal frame on the normal bundle $N\Sigma _0$.
- dynamically for $t > 0$, where we use
  The heat coordinates on the manifolds $\Sigma _t$.
  The heat gauge for the orthonormal frame on the normal bundle $N\Sigma$.

One simple example of initial data allowed by our theorem consists of graph submanifolds with defining functions $u_1$, $u_2$, of the form

$$\begin{aligned} \Sigma _0 = \{ x, u_1(x), u_2(x); x \in {{\mathbb {R}}}^d\} \end{aligned}$$

Here one may simply take $u_1$ and $u_2$ to be small in $H^{s+2}$, with the added low frequency control in the $Y^{lo}_0$ space in dimension two. However, the $H^{s+2}$ control is only needed at high frequency, while at low frequency it suffices to have control only in homogeneous norms $\dot{H}^{\frac{d}{2}+1-\delta }$ with $\delta > 0$. This allows for perturbations which are not small in any uniform norm.

#### Example 1.3.1

(*Bump-like sub-manifolds*) Let $\phi _i,\ i=1,2$ be Schwartz functions. Then for small $\epsilon > 0$ and $\delta > 0$, the manifold $\Sigma _0$ given by the defining functions

$$\begin{aligned} u_j = \epsilon ^{\frac{d}{2} -2 +\delta } \phi _j(\epsilon x) \end{aligned}$$

satisfies the hypotheses of our theorem. with $\epsilon >0$ sufficiently small. This manifold is not a small perturbation of the Euclidean plane in low dimension.

#### Example 1.3.2

(*Sub-manifolds with nontrivial asymptotics*) For small $\epsilon _j > 0$ and $\delta > 0$, the manifold $\Sigma _0$ given by the defining functions

$$\begin{aligned} u_j = \epsilon _j (1+x^2)^{1-\frac{d}{4}-\delta } \end{aligned}$$

satisfies the hypotheses of our theorem. with $\epsilon _j>0$ sufficiently small. This manifold is also not a small perturbation of the Euclidean plane.

In the next section we reformulate the (SMCF) equations as a quasilinear Schrödinger evolution for good scalar complex variable $\lambda$, which is exactly the second fundamental form but represented in the good gauge. There we provide an alternate formulation of the above result, as a well-posedness result for the $\lambda$ equation. In the final section of the paper we close the circle and show that one can reconstruct the full (SMCF) flow starting from the good variable $\lambda$.

Once our problem is rephrased as a nonlinear Schrödinger evolution, one may compare its study with earlier results on general quasilinear Schrödinger evolutions. This story begins with the classical work of Kenig–Ponce–Vega [12–14], where local well-posedness is established for more regular and localized data. Lower regularity results in translation invariant Sobolev spaces were later established by Marzuola–Metcalfe–Tataru [20–22]. The local energy decay properties of the Schrödinger equation, as developed earlier in [2, 3, 5, 6] play a key role in these results. While here we are using some of the ideas in the above papers, the present problem is both more complex and exhibits additional structure. Because of this, new ideas and more work are required in order to close the estimates required for both the full problem and for its linearization.

### An Overview of the Paper

Our first objective in this article will be to provide a self-contained formulation of the (SMCF) flow, interpreted as a nonlinear Schrödinger equation for a well chosen variable. This variable, denoted by $\lambda$, represents the second fundamental form on $\Sigma _t$, in complex notation. We remark that in our earlier paper [9] we have used instead the complex representation $\psi$ of the mean curvature ${\textbf{H}}$ as the good variable, and $\lambda$ was uniquely determined by $\psi$ via an elliptic div-curl system. However, solving this system in two dimensions is a delicate matter, which is why here we switch to $\lambda$. The slight downside of this strategy is that the components of $\lambda$ are not independent, and instead satisfy a set of compatibility conditions which need to be propagated along the flow.

In addition to the main variable $\lambda$, we will use several dependent variables, as follows:

- The Riemannian metric *g* on $\Sigma _t$.
- The magnetic potential *A*, associated to the natural connection on the normal bundle $N \Sigma _t$.

These additional variables will be viewed as uniquely determined by our main variable $\lambda$ and initial metric $g_0$ in a dynamical fashion. This is first done at the initial time by choosing harmonic coordinates on $\Sigma _0$, respectively the Coulomb gauge on $N \Sigma _0$. Finally, our dynamical gauge choice also has two components:

(i) The choice of coordinates on $\Sigma _t$; here we use heat coordinates, with suitable boundary conditions at infinity.
(ii) The choice of the orthonormal frame on $N\Sigma _t$; here we use the heat gauge, again assuming flatness at infinity.

To begin this analysis, in the next section we describe the gauge choices, so that by the end we obtain

- A nonlinear Schrödinger equation for $\lambda$, see (2.28).
- A parabolic system (2.29) for the dependent variables ${{\mathcal {S}}}=(g,A)$, together with suitable compatibility conditions (constraints).

Setting the stage to solve these equations, in Section 3 we describe the function spaces for both $\lambda$ and ${{\mathcal {S}}}$. This is done at two levels, first at fixed time, which is needed in order to track data sets, and then in the space-time setting, which is needed in order to solve both the heat flows (2.29) and the Schrödinger evolution (2.28). The fixed time spaces are classical Sobolev spaces, with matched regularities for all the components. The main space-time norms are the so called local energy spaces associated to the Schrödinger evolution, as developed in [20–22]. In addition, we also use parabolic mixed norm spaces, which capture the regularity gain in the heat flows.

We begin our analysis in Section 4, where we place the initial data in the harmonic/Coulomb gauge. In higher dimension this analysis was already carried out in our earlier paper [9]. Thus our emphasis here is on the two dimensional case, where some additional low frequency issues arise in connection with the *Y* norms for the metric *g*. Compared to our earlier article [9], here we are able to improve the analysis and relax the low frequency component of the *Y* norm. This suffices in dimension three, but is only borderline in dimension two, which is why we add the low frequency *Y* bound to the hypothesis of Theorem 1.3.

Next, in Section 5, we consider the solvability of the parabolic system (2.29). We will do this in two steps. First we prove that this system is solvable in the space ${{\mathcal {E}}}^s$. Then we prove space-time bounds for the metric *h* in local energy spaces; the latter will be needed in the study of the Schrödinger evolution (2.28).

Finally, we turn our attention to the Schrödinger system (2.28), whose study may be compared with earlier results on general quasilinear Schrödinger evolutions. This begins with the classical work of Kenig–Ponce–Vega [12–14], where local well-posedness is established for more regular and localized data. Lower regularity results in translation invariant Sobolev spaces were later established by Marzuola–Metcalfe–Tataru [20–22]. The local energy decay properties of the Schrödinger equation, as developed earlier in [2, 3, 5, 6] play a key role in these results. Here we are following a similar track, though the present problem is both more complex and exhibits additional structure. Because of this, new ideas and more work are required in order to close the estimates required for both the full problem and for its linearization.

We divide our approach in several steps. In Section 6 we establish several multilinear and nonlinear estimates in our space-time function spaces. These are then used in Section 7 in order to prove local energy decay bounds first for the linear paradifferential Schrödinger flow, and then for a full linear Schrödinger flow associated to the linearization of our main evolution.

The analysis is completed in Section 8, where we combine the linear heat flow bounds and the linear Schrödinger bounds in order to (i) construct solutions for the full nonlinear Schrödinger flow, and (ii) to prove the uniqueness and continuous dependence of the solutions. The solutions are initially constructed without reference to the constraint equations, but then we prove that the constraints are indeed satisfied, by propagating them from the initial time.

Last but not least, in the last section we prove that the full set of variables $(\lambda ,g,A)$ suffice in order to uniquely reconstruct the defining function *F* for the evolving surfaces $\Sigma _t$, as $H^{s+2}_{loc}$ manifolds. More precisely, with respect to the parametrization provided by our chosen gauge, *F* has regularity

$$\begin{aligned} \partial _t F,\ \partial _x^2 F \in C[0,1;H^s]. \end{aligned}$$

## The Differentiated Equations and the Gauge Choice

The goal of this section is to introduce our main independent variable $\lambda$, which represents the second fundamental form in complex notation, as well as the following auxiliary variables: the metric *g*, the connection coefficients *A* for the normal bundle. For $\lambda$ we start with (1.1) and derive a nonlinear Schödinger type system (2.28), with coefficients depending on ${{\mathcal {S}}}= (g,A)$. Under suitable gauge conditions, the auxiliary variables ${{\mathcal {S}}}$ are shown to satisfy a parabolic system (2.29), as well as a natural set of constraints. We conclude the section with a gauge formulation of our main result, see Theorem 2.5. Here we will introduce the heat coordinates and heat gauge in detail. For some of the detailed derivations, we refer to section 2 in [9].

### The Riemannian Metric *g* and the Second Fundamental Form

Let $(\Sigma ^d,g)$ be a *d*-dimensional oriented manifold and let $({{\mathbb {R}}}^{d+2},g_{{{\mathbb {R}}}^{d+2}})$ be $(d+2)$-dimensional Euclidean space. Let $\alpha ,{\beta },\gamma ,\cdots \in \{1,2,\cdots ,d\}$. Considering the immersion $F:\Sigma \rightarrow ({{\mathbb {R}}}^{d+2},g_{{{\mathbb {R}}}^{d+2}})$, we obtain the induced metric *g* in $\Sigma$,

2.1 $$\begin{aligned} g_{\alpha {\beta }}=\partial _{x_{\alpha }} F\cdot \partial _{x_{{\beta }}} F. \end{aligned}$$

We denote the inverse of the matrix $g_{\alpha {\beta }}$ by $g^{\alpha {\beta }}$, i.e.

$$\begin{aligned} g^{\alpha {\beta }}:=(g_{\alpha {\beta }})^{-1},\qquad g_{\alpha \gamma }g^{\gamma {\beta }}=\delta _{\alpha }^{{\beta }}. \end{aligned}$$

Let $\nabla$ be the cannonical Levi–Civita connection on $\Sigma$ associated with the induced metric *g*. A direct computation shows that on the Riemannian manifold $(\Sigma ,g)$ we have the Christoffel symbols

$$\begin{aligned} \Gamma ^{\gamma }_{\alpha {\beta }}=\ g^{\gamma \sigma }\Gamma _{\alpha {\beta },\sigma }=\ g^{\gamma \sigma }\partial ^2_{\alpha {\beta }}F\cdot \partial _\sigma F. \end{aligned}$$

For any tensor $T^{\alpha _1\cdots \alpha _r}_{{\beta }_1\cdots {\beta }_s}\textrm{d}x^{\beta _1}\otimes ...\textrm{d}x^{\beta _s}\otimes \frac{\partial }{\partial x^{\alpha _1}}\otimes ...\otimes \frac{ \partial }{\partial x^{\alpha _r}}$, we define its *covariant derivative* as follows

2.2 $$\begin{aligned} \nabla _\gamma T^{\alpha _1\cdots \alpha _r}_{{\beta }_1\cdots {\beta }_s}=\partial _\gamma T^{\alpha _1\cdots \alpha _r}_{{\beta }_1\cdots {\beta }_s}-\sum _{i=1}^s\Gamma _{\gamma {\beta }_i}^\sigma T^{\alpha _1\cdots \alpha _r}_{{\beta }_1\cdots {\beta }_{i-1}\sigma {\beta }_{i+1}\cdots {\beta }_s}+\sum _{j=1}^r\Gamma _{\gamma \delta }^{\alpha _j} T^{\alpha _1\cdots \alpha _{j-1}\delta \alpha _{j+1}\cdots \alpha _r}_{{\beta }_1\cdots {\beta }_s}.\nonumber \\ \end{aligned}$$

Hence, the Laplace–Beltrami operator $\Delta _g$ can be written in the form

$$\begin{aligned} \Delta _g f&=\ {\text {tr}}\nabla ^2 f=g^{\alpha {\beta }}(\partial _{\alpha {\beta }}^2f-\Gamma ^{\gamma }_{\alpha {\beta }}\partial _{\gamma } f), \end{aligned}$$

for any twice differentiable function $f:\Sigma \rightarrow {{\mathbb {R}}}$. The curvature *R* on the Riemannian manifold $(\Sigma ,g)$ is given by

$$\begin{aligned} R_{\gamma \alpha {\beta }}^{\sigma }=\partial _{\alpha } \Gamma _{{\beta }\gamma }^{\sigma }-\partial _{{\beta }} \Gamma _{\alpha \gamma }^{\sigma } +\Gamma _{{\beta }\gamma }^m\Gamma _{\alpha m}^{\sigma } -\Gamma _{\alpha \gamma }^m\Gamma _{{\beta }m}^{\sigma }. \end{aligned}$$

We also have

2.3 $$\begin{aligned} R_{\sigma \gamma \alpha {\beta }}=\partial _{\alpha } \Gamma _{{\beta }\gamma ,\sigma }-\partial _{{\beta }} \Gamma _{\alpha \gamma ,\sigma } +\Gamma _{{\beta }\sigma }^m\Gamma _{\alpha \gamma ,m} -\Gamma _{\alpha \sigma }^m\Gamma _{{\beta }\gamma ,m}, \end{aligned}$$

and the Ricci curvature

$$\begin{aligned} {\text {Ric}}_{\alpha {\beta }}=R^{\sigma }_{\alpha \sigma {\beta }}=g^{\sigma \gamma }R_{\gamma \alpha \sigma {\beta }}. \end{aligned}$$

Next, we derive the second fundamental form for $\Sigma$. Let ${\bar{\nabla }}$ be the Levi–Civita connection in $({{\mathbb {R}}}^{d+2},g_{{{\mathbb {R}}}^{d+2}})$ and let ${\textbf{h}}$ be the second fundamental form for $\Sigma$ as an embedded manifold. For any vector fields $u,v\in T_{*}\Sigma$, the Gauss relation is

$$\begin{aligned} {\bar{\nabla }}_u F_{*}v=F_{*}(\nabla _u v)+ {\textbf{h}}(u,v). \end{aligned}$$

Then we have

$$\begin{aligned} {\textbf{h}}_{\alpha {\beta }}={\textbf{h}}(\partial _{\alpha },\partial _{{\beta }})={\bar{\nabla }}_{\partial _{\alpha }} \partial _{{\beta }} F-F_{*}(\nabla _{\partial _{\alpha }}\partial _{{\beta }}) =\partial _{\alpha {\beta }}^2 F-\Gamma _{\alpha {\beta }}^{\gamma } \partial _{\gamma } F. \end{aligned}$$

This gives the mean curvature ${\textbf{H}}$ at *F*(*x*),

$$\begin{aligned} {\textbf{H}}={\text {tr}}_g {\textbf{h}}=g^{\alpha {\beta }}{\textbf{h}}_{\alpha {\beta }}=g^{\alpha {\beta }}(\partial ^2_{\alpha {\beta }}F-\Gamma ^{\gamma }_{\alpha {\beta }}\partial _{\gamma } F)=\Delta _g F. \end{aligned}$$

Hence, the *F*-equation in (1.1) is rewritten as

$$\begin{aligned} (\partial _t F)^{\perp }=J(F)\Delta _g F=J(F)g^{\alpha {\beta }}(\partial ^2_{\alpha {\beta }}F-\Gamma ^{\gamma }_{\alpha {\beta }}\partial _{\gamma } F). \end{aligned}$$

This equation is still independent of the choice of coordinates in $\Sigma ^d$.

### The Complex Structure Equations

Here we introduce a complex structure on the normal bundle $N\Sigma _t$. This is achieved by choosing $\{\nu _1,\nu _2\}$ to be an orthonormal basis of $N\Sigma _t$ such that

$$\begin{aligned} J\nu _1=\nu _2,\quad J\nu _2=-\nu _1. \end{aligned}$$

Such a choice is not unique; in making it we introduce a second component to our gauge group, namely the group of sections of an *SU*(1) bundle over $I \times {{\mathbb {R}}}^d$.

The vectors $\{ F_1,\cdots ,F_d,\nu _1,\nu _2\}$ form a frame at each point on the manifold $(\Sigma ,g)$, where $F_{\alpha }$ for $\alpha \in \{1,\cdots ,d\}$ are defined as

$$\begin{aligned} F_{\alpha }=\partial _{\alpha }F. \end{aligned}$$

We define the tensors $\kappa _{\alpha {\beta }},\ \tau _{\alpha {\beta }}$, the connection coefficients $A_{\alpha }$ and the temporal component *B* of the connection in the normal bundle by

$$\begin{aligned} \kappa _{\alpha {\beta }}:=\partial _{\alpha } F_{{\beta }}\cdot \nu _1,\quad \tau _{\alpha {\beta }}:=\partial _{\alpha } F_{{\beta }}\cdot \nu _2,\quad A_{\alpha }=\partial _{\alpha }\nu _1\cdot \nu _2,\quad B=\partial _t \nu _1\cdot \nu _2. \end{aligned}$$

Then we complexify the normal frame $\{\nu _1,\nu _2\}$ and second fundamental form as

$$\begin{aligned} m=\nu _1+i\nu _2,\quad \lambda _{\alpha {\beta }}=\kappa _{\alpha {\beta }}+i\tau _{\alpha {\beta }}. \end{aligned}$$

Here we can define the *complex scalar mean curvature* $\psi$ to be

2.4 $$\begin{aligned} \psi :={\text {tr}}\,\lambda =g^{\alpha {\beta }}\lambda _{\alpha {\beta }}. \end{aligned}$$

Our objective for the rest of this section will be to interpret the (SMCF) equation as a nonlinear Schrödinger evolution for $\lambda$, by making suitable gauge choices. We remark that the action of sections of the *SU*(1) bundle is given by

2.5 $$\begin{aligned} \psi \rightarrow e^{i \theta \psi }, \quad \lambda \rightarrow e^{i \theta \lambda }, \quad m \rightarrow e^{i\theta } m, \quad A_\alpha \rightarrow A_\alpha - \partial _\alpha \theta . \end{aligned}$$

for a real valued function $\theta$.

If we differentiate the frame, we obtain a set of structure equations of the following type

2.6 $$\begin{aligned} \left\{ \begin{aligned}&\partial _{\alpha }F_{{\beta }}=\Gamma ^{\gamma }_{\alpha {\beta }}F_{\gamma }+\mathop {\textrm{Re}}\nolimits (\lambda _{\alpha {\beta }}{\bar{m}}),\\&\partial _{\alpha }^A m=-\lambda ^{\gamma }_{\alpha } F_{\gamma }, \end{aligned}\right. \end{aligned}$$

where $$\partial _{\alpha }^A=\partial _{\alpha }+iA_{\alpha }$$.

### The Gauss and Codazzi Relations

The Gauss and Codazzi equations are derived from the equality of second derivatives $\partial _{\alpha }\partial _{{\beta }}F_{\gamma }=\partial _{{\beta }}\partial _{\alpha }F_{\gamma }$ for the tangent vectors on the submanifold $\Sigma$ and for the normal vectors respectively. Here we use the Gauss and Codazzi relations to derive the Riemannian curvature, the first compatibility condition and a symmetry.

By the structure equations (2.6), we get

2.7 $$\begin{aligned} \begin{aligned} \partial _{\alpha }\partial _{\beta }F_{\gamma }&=(\partial _{\alpha }\Gamma _{{\beta }\gamma }^{\sigma }+\Gamma _{{\beta }\gamma }^{\mu }\Gamma ^{\sigma }_{\alpha \mu } -\mathop {\textrm{Re}}\nolimits (\lambda _{{\beta }\gamma }{\bar{\lambda }}_{\alpha }^{\sigma }))F_{\sigma }\\&\quad +\mathop {\textrm{Re}}\nolimits [(\partial _{\alpha }^A\lambda _{{\beta }\gamma }+\Gamma ^{\sigma }_{{\beta }\gamma }\lambda _{\alpha \sigma }){\bar{m}}]. \end{aligned} \end{aligned}$$

Then in view of $\partial _{\alpha }\partial _{{\beta }} F_{\gamma }=\partial _{{\beta }}\partial _{\alpha } F_{\gamma }$ and equating the coefficients of the tangent vectors, we obtain

$$\begin{aligned} \partial _{\alpha }\Gamma _{{\beta }\gamma }^{\sigma }+\Gamma _{{\beta }\gamma }^{\mu }\Gamma ^{\sigma }_{\alpha \mu }-\partial _{{\beta }}\Gamma _{\alpha \gamma }^{\sigma }-\Gamma _{\alpha \gamma }^{\mu }\Gamma ^{\sigma }_{{\beta }\mu }=\mathop {\textrm{Re}}\nolimits (\lambda _{{\beta }\gamma }{\bar{\lambda }}_{\alpha }^{\sigma }-\lambda _{\alpha \gamma }{\bar{\lambda }}_{{\beta }}^{\sigma }). \end{aligned}$$

This gives the Riemannian curvature

2.8 $$\begin{aligned} R_{\sigma \gamma \alpha {\beta }}=\mathop {\textrm{Re}}\nolimits (\lambda _{{\beta }\gamma }{\bar{\lambda }}_{\alpha \sigma }-\lambda _{\alpha \gamma }{\bar{\lambda }}_{{\beta }\sigma }), \end{aligned}$$

which is a complex formulation of the Gauss equation. Correspondingly we obtain the the Ricci curvature

2.9 $$\begin{aligned} {\text {Ric}}_{\gamma {\beta }}=\mathop {\textrm{Re}}\nolimits (\lambda _{\gamma {\beta }}{\bar{\psi }}-\lambda _{\gamma \alpha }{\bar{\lambda }}_{{\beta }}^{\alpha }). \end{aligned}$$

After equating the coefficients of the vector *m* in (2.7), we obtain

$$\begin{aligned} \partial ^A_{\alpha }\lambda _{{\beta }\gamma }+\Gamma _{{\beta }\gamma }^{\sigma }\lambda _{\alpha \sigma }=\partial ^A_{{\beta }}\lambda _{\alpha \gamma }+\Gamma _{\alpha \gamma }^{\sigma }\lambda _{{\beta }\sigma }, \end{aligned}$$

By the definition of covariant derivatives (2.2), we obtain the complex formulation of the Codazzi equation, namely

2.10 $$\begin{aligned} \nabla ^A_{\alpha } \lambda _{{\beta }\gamma }=\nabla ^A_{{\beta }}\lambda _{\alpha \gamma }. \end{aligned}$$

Next, we use the relation $\partial _{\alpha }\partial _{{\beta }}m=\partial _{{\beta }}\partial _{\alpha }m$ in order to derive a compatibility condition between the connection *A* in the normal bundle and the second fundamental form. Indeed, from $\partial _{\alpha }\partial _{{\beta }}m=\partial _{{\beta }}\partial _{\alpha }m$ we obtain the commutation relation

2.11 $$\begin{aligned} {[}\partial ^A_{\alpha },\partial ^A_{{\beta }}]m=i(\partial _{\alpha } A_{{\beta }}-\partial _{{\beta }} A_{\alpha })m. \end{aligned}$$

By (2.6) we have

$$\begin{aligned} \partial ^A_{\alpha }\partial ^A_{{\beta }} m&=-\partial ^A_{\alpha }(\lambda ^{\gamma }_{{\beta }} F_{\gamma })=-(\partial ^A_{\alpha }\lambda ^{\sigma }_{{\beta }}+\lambda ^{\gamma }_{{\beta }}\Gamma ^{\sigma }_{\alpha \gamma })F_{\sigma }-\lambda ^{\gamma }_{{\beta }}\mathop {\textrm{Re}}\nolimits (\lambda _{\alpha \gamma }{\bar{m}})). \end{aligned}$$

Then multiplying (2.11) by *m* yields

$$\begin{aligned} \partial _{\alpha } A_{{\beta }}-\partial _{{\beta }} A_{\alpha }=\mathop {\textrm{Im}}\nolimits (\lambda ^{\gamma }_{\alpha }{\bar{\lambda }}_{{\beta }\gamma }). \end{aligned}$$

This gives the compatibility condition for the curvature *A*,

2.12 $$\begin{aligned} \nabla _{\alpha } A_{{\beta }}-\nabla _{{\beta }} A_{\alpha }=\mathop {\textrm{Im}}\nolimits (\lambda ^{\gamma }_{\alpha }{\bar{\lambda }}_{{\beta }\gamma }), \end{aligned}$$

which can be seen as the complex form of the Ricci equations. We remark that, by equating the coefficients of the tangent vectors in (2.11) , we also obtain the relation (2.10) again.

### The Motion of the Frame $\{F_1,\cdots ,F_d,m\}$ under (SMCF)

Here we derive the equations of motion for the frame, assuming that the immersion *F* satisfying (1.1).

We begin by rewriting the SMCF equations in the form

$$\begin{aligned} \partial _t F=J(F){\textbf{H}}(F)+V^{\gamma } F_{\gamma }, \end{aligned}$$

where $V^{\gamma }$ is a vector field on the manifold $\Sigma$, which in general depends on the choice of coordinates. By the definition of *m* and $\lambda _{\alpha {\beta }}$, the above *F*-equation is rewritten as

2.13 $$\begin{aligned} \partial _t F=-\mathop {\textrm{Im}}\nolimits (\psi {\bar{m}})+V^{\gamma } F_{\gamma }. \end{aligned}$$

Then by (2.13), the structure equations (2.6) and the orthogonality relation $m\bot F_{\alpha }=0$ we obtain the following equations of motion for the frame

2.14 $$\begin{aligned} \left\{ \begin{aligned}&\partial _t F_{\alpha }=-\mathop {\textrm{Im}}\nolimits (\partial ^A_{\alpha } \psi {\bar{m}}-i\lambda _{\alpha \gamma }V^{\gamma } {\bar{m}})+[\mathop {\textrm{Im}}\nolimits (\psi {\bar{\lambda }}^{\gamma }_{\alpha })+\nabla _{\alpha } V^{\gamma }]F_{\gamma },\\&\partial ^{B}_t m=-i(\partial ^{A,\alpha } \psi -i\lambda ^{\alpha }_{\gamma }V^{\gamma } )F_{\alpha }. \end{aligned}\right. \end{aligned}$$

where covariant derivative $\partial _t^B=\partial _t +iB$ and $B=\langle \partial _t \nu _1,\nu _2\rangle$ is the temporal component of the connection in the normal bundle.

From this we obtain the evolution equation for the metric *g*. By the definition of the induced metric *g* (2.1) and (2.14) , we have

2.15 $$\begin{aligned} \partial _t g_{\alpha {\beta }}&=\ 2\mathop {\textrm{Im}}\nolimits (\psi {\bar{\lambda }}_{\alpha {\beta }})+\nabla _{\alpha }V_{{\beta }}+\nabla _{{\beta }}V_{\alpha }. \end{aligned}$$

So far, the choice of *V* has been unspecified; it depends on the choice of coordinates on our manifold as the time varies.

### The Motion of *A* and $\lambda$ Under (SMCF)

Here we use the equations of motion for the frame in (2.14) in order to repeat the computations of Section 2.3 with respect to time differentiation, with the aim of computing the time derivative of both $\lambda$ and *A*. We start from the commutation relation

$$\begin{aligned} {[}\partial ^{B}_t,\partial ^A_{\alpha }]m=i(\partial _t A_{\alpha }-\partial _{\alpha } B)m. \end{aligned}$$

In order, for the left-hand side, by (2.6) and (2.14) we have

$$\begin{aligned} \partial ^{B}_t\partial ^A_{\alpha } m&=-[\partial ^{B}_t\lambda ^{\sigma }_{\alpha }+\lambda ^{\gamma }_{\alpha }(\mathop {\textrm{Im}}\nolimits (\psi {\bar{\lambda }}^{\sigma }_{\gamma })+\nabla _{\gamma }V^{\sigma })]F_{\sigma }+\lambda ^{\gamma }_{\alpha }\mathop {\textrm{Im}}\nolimits (\partial ^A_{\gamma }\psi {\bar{m}}-i\lambda _{\gamma \sigma }V^{\sigma }{\bar{m}}), \end{aligned}$$

and

$$\begin{aligned} \partial ^A_{\alpha }\partial ^{B}_t m&=-i\nabla ^A_{\alpha }(\partial ^{A,\sigma } \psi -i\lambda ^{\sigma }_{\gamma }V^{\gamma } )F_{\sigma }-i(\partial ^{A,\sigma } \psi -i\lambda ^{\sigma }_{\gamma }V^{\gamma } )\mathop {\textrm{Re}}\nolimits (\lambda _{\alpha \gamma }{\bar{m}}). \end{aligned}$$

Then by the above three equalities, equating the coefficients of the tangent vectors and the normal vector *m*, we obtain the evolution equation for $\lambda$

2.16 $$\begin{aligned} \partial ^{B}_t\lambda ^{\sigma }_{\alpha }+\lambda ^{\gamma }_{\alpha }(\mathop {\textrm{Im}}\nolimits (\psi {\bar{\lambda }}^{\sigma }_{\gamma })+\nabla _{\gamma } V^{\sigma })=i\nabla ^A_{\alpha }(\partial ^{A,\sigma } \psi -i\lambda ^{\sigma }_{\gamma }V^{\gamma } ), \end{aligned}$$

as well as the compatibility condition (curvature relation)

2.17 $$\begin{aligned} \partial _t A_{\alpha }-\partial _{\alpha } B = \mathop {\textrm{Re}}\nolimits (\lambda _{\alpha }^{\gamma }{\bar{\partial }}^A_{\gamma }{\bar{\psi }})-\mathop {\textrm{Im}}\nolimits (\lambda ^\gamma _\alpha {\bar{\lambda }}_{\gamma \sigma })V^\sigma . \end{aligned}$$

### The Equations for the Connection *A* in the Coulomb Gauge and the Heat Gauge

Here we take the first step towards fixing the gauge, and consider the choice of the orthonormal frame in $N\Sigma$. Our starting point consists of the curvature relations (2.12) at fixed time, respectively (2.17) dynamically, together with the gauge group (2.5). We will fix the gauge in two steps, first in a static, elliptic fashion at the initial time, and then dynamically, using a heat flow, for later times.

At the initial time $t=0$ we obtain an elliptic system for *A* by imposing the Coulomb gauge condition

2.18 $$\begin{aligned} \nabla ^\alpha A_\alpha =0. \end{aligned}$$

As in [9], this yields

#### Lemma 2.1

(Div-curl system for *A*) Under the Coulomb gauge condition (2.18), the connection *A* solves

2.19 $$\begin{aligned} \nabla ^\alpha A_\alpha =0,\quad \nabla _\alpha A_{\beta }-\nabla _{\beta }A_\alpha =\mathop {\textrm{Im}}\nolimits (\lambda ^\gamma _\alpha {\bar{\lambda }}_{{\beta }\gamma }). \end{aligned}$$

In our previous work [9], the connection coefficients *A* and *B* were determined via the Coulomb gauge condition (2.18) at all times. Instead, in this article we only enforce the Coulomb gauge condition at the initial time $t = 0$, while for $t > 0$ we adopt from [19] a different gauge condition called the *parabolic gauge* or *heat gauge*. This is defined by the relation

2.20 $$\begin{aligned} \nabla ^\alpha A_\alpha =B, \end{aligned}$$

which in turn yields a parabolic equation for *A*:

#### Lemma 2.2

(Parabolic equations for *A*) Under the heat gauge condition (2.20), the connection *A* solves

2.21 $$\begin{aligned} (\partial _t -\nabla _\sigma \nabla ^\sigma ) A_\alpha= & {} \nabla ^\sigma \mathop {\textrm{Im}}\nolimits (\lambda ^\gamma _\alpha {\bar{\lambda }}_{\sigma \gamma })-{\text {Ric}}_{\alpha \delta }A^\delta \nonumber \\{} & {} +\mathop {\textrm{Re}}\nolimits (\lambda ^\gamma _\alpha \overline{\nabla ^A_\gamma \psi })-\mathop {\textrm{Im}}\nolimits (\lambda ^\gamma _\alpha {\bar{\lambda }}_{\gamma \sigma })V^\sigma . \end{aligned}$$

#### Proof

Since by (2.12) we have

$$\begin{aligned} \nabla _\alpha \nabla ^\sigma A_\sigma&=[\nabla _\alpha ,\nabla ^\sigma ]A_\sigma +\nabla ^\sigma (\nabla _\alpha A_\sigma -\nabla _\sigma A_\alpha )+\nabla ^\sigma \nabla _\sigma A_\alpha \\&=-{\text {Ric}}_{\alpha \sigma }A^\sigma +\nabla ^\sigma \mathop {\textrm{Im}}\nolimits (\lambda ^\gamma _\alpha {\bar{\lambda }}_{\sigma \gamma })+\nabla ^\sigma \nabla _\sigma A_\alpha \end{aligned}$$

Then the equations (2.21) is obtained from (2.17) and the heat gauge (2.20). $\square$

### The Equations for the Metric *g* in Harmonic Coordinates and Heat Coordinates

Here we take the next step towards fixing the gauge, by choosing to work in harmonic coordinates at $t=0$ and heat coordinates for $t>0$. Precisely, at the initial time $t=0$ we will require the coordinate functions $\{x_{\alpha },\alpha =1,\cdots ,d\}$ to be globally Lipschitz solutions of the elliptic equations

2.22 $$\begin{aligned} \Delta _g x_{\alpha }=0. \end{aligned}$$

This determines the coordinates uniquely modulo time dependent affine transformations. This remaining ambiguity will be removed later on by imposing suitable boundary conditions at infinity. After this, the only remaining degrees of freedom in the choice of coordinates at $t = 0$ will be given by translations and rigid rotations.

Here we interpret the above harmonic coordinate condition at fixed time as an elliptic equation for the metric *g*. The equations (2.22) may be expressed in terms of the Christoffel symbols $\Gamma$, which must satisfy the condition

2.23 $$\begin{aligned} g^{\alpha {\beta }}\Gamma ^{\gamma }_{\alpha {\beta }}=0,\quad \textrm{for}\ \gamma =1,\cdots ,d. \end{aligned}$$

This leads to an equation for the metric *g*:

#### Lemma 2.3

(Elliptic equations of *g*, Lemma 2.4 [9]) In harmonic coordinates, the metric *g* satisfies

2.24 $$\begin{aligned} \begin{aligned} g^{\alpha {\beta }}\partial ^2_{\alpha {\beta }}g_{\gamma \sigma }&= [-\partial _{\gamma } g^{\alpha {\beta }}\partial _{{\beta }} g_{\alpha \sigma }-\partial _{\sigma } g^{\alpha {\beta }}\partial _{{\beta }} g_{\alpha \gamma }+\partial _{\gamma } g_{\alpha {\beta }}\partial _{\sigma } g^{\alpha {\beta }}]\\&\quad +2g^{\alpha {\beta }}\Gamma _{\sigma \alpha ,\nu }\Gamma ^{\nu }_{{\beta }\gamma }-2\mathop {\textrm{Re}}\nolimits (\lambda _{\gamma \sigma }{\bar{\psi }}-\lambda _{\alpha \gamma }{\bar{\lambda }}_{\sigma }^{\alpha }). \end{aligned} \end{aligned}$$

For latter times $t>0$ we will introduce the *heat gauge*, where we require the coordinate functions $\{x^\alpha ,\alpha =1,\cdots ,d\}$ to be global Lipschitz solutions of the heat equations

$$\begin{aligned} (\partial _t - \Delta _g - V^\gamma \partial _\gamma ) x_\alpha = 0. \end{aligned}$$

This can be rewritten as

$$\begin{aligned} \Delta _g x^\gamma =- V^\gamma , \end{aligned}$$

and can also be expressed in terms of the Christoffel symbols $\Gamma$, namely,

2.25 $$\begin{aligned} g^{\alpha {\beta }}\Gamma ^\gamma _{\alpha {\beta }}=V^\gamma . \end{aligned}$$

Once a choice of coordinates is made at the initial time, the coordinates will be uniquely determined later on by this gauge condition.

With the advection field *V* fixed via the heat coordinate condition (2.25), we can derive a parabolic equation for the metric *g*.

#### Lemma 2.4

(Parabolic equations for metric *g*) Under the condition (2.25), the metric *g* solves

2.26 $$\begin{aligned} \begin{aligned} \partial _t g_{\mu \nu }-g^{\alpha {\beta }}\partial ^2_{\alpha {\beta }}g_{\mu \nu }&=2{\text {Ric}}_{\mu \nu }+2\mathop {\textrm{Im}}\nolimits (\psi {\bar{\lambda }}_{\mu \nu })-2g^{\alpha {\beta }}\Gamma _{\mu {\beta },\sigma }\Gamma ^\sigma _{\alpha \nu }\\&\quad +\partial _{\mu }g^{\alpha {\beta }}\Gamma _{\alpha {\beta },\nu }+\partial _{\nu }g^{\alpha {\beta }}\Gamma _{\alpha {\beta },\mu }. \end{aligned} \end{aligned}$$

#### Proof

By the relation (2.25) we have

$$\begin{aligned} \nabla _\mu V_\nu = g^{\alpha {\beta }}\partial _\mu \Gamma _{\alpha {\beta },\nu }+\partial _\mu g^{\alpha {\beta }}\Gamma _{\alpha {\beta },\nu }-g^{\alpha {\beta }}\Gamma _{\mu \nu }^\sigma \Gamma _{\alpha {\beta },\sigma } \end{aligned}$$

Using the expression for $\Gamma$ and for the Riemannian curvature (2.3) we have

$$\begin{aligned} g^{\alpha {\beta }}(\partial _\mu \Gamma _{\alpha {\beta },\nu }+\partial _\nu \Gamma _{\alpha {\beta },\mu })&=g^{\alpha {\beta }}\left[ \partial _\mu \left( \partial _\alpha g_{{\beta }\nu }-\frac{1}{2}\partial _\nu g_{\alpha {\beta }}\right) +\partial _\nu \left( \partial _\alpha g_{{\beta }\mu }-\frac{1}{2}\partial _\mu g_{\alpha {\beta }}\right) \right] \\&= g^{\alpha {\beta }}[\partial _\alpha (\partial _\mu g_{{\beta }\nu }+\partial _\nu g_{{\beta }\mu }-\partial _{\beta }g_{\mu \nu })-\partial _\mu (\partial _\nu g_{\alpha {\beta }}+\partial _\alpha g_{\nu {\beta }}\\&\quad -\partial _{\beta }g_{\alpha \nu })+\partial ^2_{\alpha {\beta }}g_{\mu \nu }]\\&=g^{\alpha {\beta }}[2\partial _\alpha \Gamma _{\mu \nu ,{\beta }}-2\partial _{\mu }\Gamma _{\alpha \nu ,{\beta }} +\partial ^2_{\alpha {\beta }}g_{\mu \nu }]\\&= 2g^{\alpha {\beta }}(R_{{\beta }\nu \alpha \mu }-\Gamma _{\nu {\beta },\sigma }\Gamma ^\sigma _{\alpha \mu }+\Gamma _{\alpha {\beta },\sigma }\Gamma ^\sigma _{\mu \nu })+g^{\alpha {\beta }}\partial ^2_{\alpha {\beta }}g_{\mu \nu }. \end{aligned}$$

We then obtain

$$\begin{aligned} \nabla _\mu V_\nu +\nabla _\nu V_\mu =g^{\alpha {\beta }}\partial ^2_{\alpha {\beta }}g_{\mu \nu }+2{\text {Ric}}_{\mu \nu }+\partial _\mu g^{\alpha {\beta }}\Gamma _{\alpha {\beta },\nu }+\partial _\nu g^{\alpha {\beta }}\Gamma _{\alpha {\beta },\mu }-2g^{\alpha {\beta }}\Gamma _{\nu {\beta },\sigma }\Gamma ^\sigma _{\alpha \mu }. \end{aligned}$$

Combined with (2.15), this implies (2.26). $\square$

### Derivation of the Modified Schrödinger System from SMCF

Here we carry out the last step in our analysis of the equations, and obtain the main Schrödinger equation which governs the time evolution of $\lambda$.

Our starting point is the equations (2.16), which are rewritten as

$$\begin{aligned} i\partial ^{B}_t\lambda _{\alpha {\beta }}+\nabla ^A_{\alpha }\nabla ^{A}_{\beta }\psi -i\lambda ^{\gamma }_{\alpha }\mathop {\textrm{Im}}\nolimits (\psi {\bar{\lambda }}_{\gamma {\beta }})-i\lambda ^{\gamma }_{\alpha }\nabla _{{\beta }} V_{\gamma }-i\lambda _{\beta }^\gamma \nabla _\alpha V_\gamma -iV^\gamma \nabla ^A_\gamma \lambda _{\alpha {\beta }}=0, \end{aligned}$$

We use the compatibility conditions (2.4), (2.12) and (2.8) to write the second term as

$$\begin{aligned} \nabla ^A_{\alpha }\nabla ^{A}_{\beta }\psi&= \nabla ^A_\alpha \nabla ^A_{\sigma }\lambda ^\sigma _{\beta }=[\nabla ^A_\alpha ,\nabla ^A_\sigma ]\lambda ^\sigma _{\beta }+\nabla ^A_\sigma \nabla ^{A,\sigma }\lambda _{\alpha {\beta }}\\&= R_{\alpha \sigma \sigma \delta }\lambda ^\delta _{\beta }+R_{\alpha \sigma {\beta }\delta }\lambda ^{\sigma \delta }+i\mathop {\textrm{Im}}\nolimits (\lambda _{\alpha \mu }{\bar{\lambda }}^\mu _\sigma )\lambda ^\sigma _{\beta }+\nabla ^A_\sigma \nabla ^{A,\sigma }\lambda _{\alpha {\beta }}\\&=-{\text {Ric}}_{\alpha \delta }\lambda ^\delta _{\beta }+R_{\alpha \sigma {\beta }\delta }\lambda ^{\sigma \delta }+i\mathop {\textrm{Im}}\nolimits (\lambda _{\alpha \mu }{\bar{\lambda }}^\mu _\sigma )\lambda ^\sigma _{\beta }+\nabla ^A_\sigma \nabla ^{A,\sigma }\lambda _{\alpha {\beta }}\\&= -\mathop {\textrm{Re}}\nolimits (\lambda _{\alpha \delta }{\bar{\psi }})\lambda ^\delta _{\beta }+\mathop {\textrm{Re}}\nolimits (\lambda _{\sigma \delta }{\bar{\lambda }}_{\alpha {\beta }}-\lambda _{\sigma {\beta }}{\bar{\lambda }}_{\alpha \delta })\lambda ^{\sigma \delta } +\lambda _{\alpha \mu }{\bar{\lambda }}^\mu _\sigma \lambda ^\sigma _{\beta }+\nabla ^A_\sigma \nabla ^{A,\sigma }\lambda _{\alpha {\beta }} \end{aligned}$$

Since

$$\begin{aligned} \frac{1}{2}\big [-\mathop {\textrm{Re}}\nolimits (\lambda _{\alpha \delta }{\bar{\psi }})\lambda ^\delta _{\beta }-\mathop {\textrm{Re}}\nolimits (\lambda _{{\beta }\delta }{\bar{\psi }})\lambda ^\delta _\alpha -i\lambda ^{\gamma }_{\alpha }\mathop {\textrm{Im}}\nolimits (\psi {\bar{\lambda }}_{\gamma {\beta }}) -i\lambda ^{\gamma }_{{\beta }}\mathop {\textrm{Im}}\nolimits (\psi {\bar{\lambda }}_{\gamma \alpha })\big ] =-\psi \mathop {\textrm{Re}}\nolimits (\lambda _{\alpha \delta }{\bar{\lambda }}^\delta _{\beta }), \end{aligned}$$

we obtain the $\lambda$-equations

2.27 $$\begin{aligned} i\partial ^{B}_t\lambda _{\alpha {\beta }} +\nabla ^A_\sigma \nabla ^{A,\sigma }\lambda _{\alpha {\beta }}= & {} iV^\gamma \nabla ^A_\gamma \lambda _{\alpha {\beta }} +i\lambda ^{\gamma }_{\alpha }\nabla _{{\beta }} V_{\gamma } +i\lambda _{\beta }^\gamma \nabla _\alpha V_\gamma +\psi \mathop {\textrm{Re}}\nolimits (\lambda _{\alpha \delta }{\bar{\lambda }}^\delta _{\beta })\nonumber \\{} & {} -\mathop {\textrm{Re}}\nolimits (\lambda _{\sigma \delta }{\bar{\lambda }}_{\alpha {\beta }}-\lambda _{\sigma {\beta }}{\bar{\lambda }}_{\alpha \delta })\lambda ^{\sigma \delta } -\lambda _{\alpha \mu }{\bar{\lambda }}^\mu _\sigma \lambda ^\sigma _{\beta }. \end{aligned}$$

In conclusion, under the heat coordinate condition $g^{\alpha {\beta }}\Gamma ^{\gamma }_{\alpha {\beta }}=V^\gamma$ and heat gauge condition $\nabla ^{\alpha }A_{\alpha }=B$, by (2.27), (2.26) and (2.21), we obtain the covariant Schrödinger equation for the complex second fundamental form tensor $\lambda$

2.28 $$\begin{aligned} \left\{ \begin{aligned}&\begin{aligned} (i\partial _t+\nabla _\sigma \nabla ^\sigma )\lambda _{\alpha {\beta }} =&i(V-2A)^\sigma \nabla _\sigma \lambda _{\alpha {\beta }}-i\nabla _\sigma A^\sigma \lambda _{\alpha {\beta }}+i\lambda ^{\gamma }_{\alpha }\nabla _{{\beta }} V_{\gamma } +i\lambda _{\beta }^\gamma \nabla _\alpha V_\gamma \\&+(B+A_\sigma A^\sigma -V_\sigma A^\sigma )\lambda _{\alpha {\beta }} +\psi \mathop {\textrm{Re}}\nolimits (\lambda _{\alpha \delta }{\bar{\lambda }}^\delta _{\beta }) \\&-\mathop {\textrm{Re}}\nolimits (\lambda _{\sigma \delta }{\bar{\lambda }}_{\alpha {\beta }}-\lambda _{\sigma {\beta }}{\bar{\lambda }}_{\alpha \delta })\lambda ^{\sigma \delta } -\lambda _{\alpha \mu }{\bar{\lambda }}^\mu _\sigma \lambda ^\sigma _{\beta }, \end{aligned}\\&\lambda (0,x) = \lambda _0(x). \end{aligned} \right. \end{aligned}$$

These equations are fully covariant, and do not depend on the gauge choices made earlier. On the other hand, our gauge choices imply that the advection field *V* and the connection coefficient *B* are determined by the metric *g* and connection *A* via (2.25), respectively, (2.20). In turn, the metric *g* and the connection coefficients *A* are determined in an parabolic fashion via the following equations

2.29 $$\begin{aligned} \left\{ \begin{aligned}&\begin{aligned} (\partial _t-g^{\alpha {\beta }}\partial ^2_{\alpha {\beta }})g_{\mu \nu }=&2{\text {Ric}}_{\mu \nu }+2\mathop {\textrm{Im}}\nolimits (\psi {\bar{\lambda }}_{\mu \nu })-2g^{\alpha {\beta }}\Gamma _{\mu {\beta },\sigma }\Gamma ^\sigma _{\alpha \nu }\\&+\partial _{\mu }g^{\alpha {\beta }}\Gamma _{\alpha {\beta },\nu }+\partial _{\nu }g^{\alpha {\beta }}\Gamma _{\alpha {\beta },\mu }. \end{aligned}\\&(\partial _t -\nabla _\sigma \nabla ^\sigma ) A_\alpha =-\nabla ^\sigma \mathop {\textrm{Im}}\nolimits (\lambda ^\gamma _\alpha {\bar{\lambda }}_{\sigma \gamma })-{\text {Ric}}_{\alpha \delta }A^\delta +\mathop {\textrm{Re}}\nolimits (\lambda ^\gamma _\alpha \overline{\nabla ^A_\gamma \psi })-\mathop {\textrm{Im}}\nolimits (\lambda ^\gamma _\alpha {\bar{\lambda }}_{\gamma \sigma })V^\sigma ,\\&V^\gamma =g^{\alpha {\beta }}\Gamma _{\alpha {\beta }}^\gamma ,\quad B=\nabla ^\alpha A_\alpha , \end{aligned}\right. \nonumber \\ \end{aligned}$$

with initial data

2.30 $$\begin{aligned} g(0,x)=g_0(x),\quad A(0,x) = A_0(x). \end{aligned}$$

These are determined at the initial time by choosing harmonic coordinates on $\Sigma _0$, respectively the Coulomb gauge for *A*.

Fixing the remaining degrees of freedom (i.e. the affine group for the choice of the coordinates as well as the time dependence of the *SU*(1) connection) we can assume that the following conditions hold at infinity in an averaged sense:

$$\begin{aligned} g(\infty ) = I_d,\quad A(\infty ) = 0. \end{aligned}$$

These are needed to insure the unique solvability of the above parabolic equations in a suitable class of functions. For the metric *g* it will be useful to use the representation

$$\begin{aligned} g = I_d + h \end{aligned}$$

so that *h* vanishes at infinity.

We have arrived at the main Schrödinger-Parabolic system (2.28)–(2.29), whose solvability is the primary objective of the rest of the paper. This system is accompanied by a family of compatibility conditions as follows:

(i) The Gauss equations (2.8) connecting the curvature *R* of *g* and $\lambda$.
(ii) The Codazzi equations (2.10) for $\lambda$.
(iii) The Ricci equations (2.12) for the curvature of *A*.
(iv) The compatibility condition (2.17) for the *B*.

We will solve the system irrespective of these compatibility conditions, but then show them to be satisfied for small solutions to the nonlinear system (2.28)–(2.29), by propagating them from the initial time $t=0$.

Now we can restate here the small data local well-posedness result for the (SMCF) system in Theorem 1.2 in terms of the above system:

#### Theorem 2.5

(Small data local well-posedness in the good gauge) Let $d\geqq 2$ and $s>\frac{d}{2}$. Then there exists $\epsilon _0>0$ sufficiently small such that, for all initial data $(\lambda _0, h_0,A_0)$ satisfying the constraints (2.10), (2.8) and (2.12) and with

2.31 $$\begin{aligned} \Vert \lambda _0\Vert _{H^s}+\Vert h_0\Vert _{{\textbf{Y}}_0^{s+2}} + \Vert A_0\Vert _{H^{s+1}} \leqq \epsilon _0, \end{aligned}$$

the modified Schrödinger system (2.28), coupled with the parabolic system (2.29) for (*h*, *A*) is locally well-posed in $l^2X^s\times {{\varvec{ {\mathcal {E}}}}}^s$ on the time interval $I=[0,1]$. Moreover, the second fundamental form $\lambda$, the metric *g* and the connection coefficients *A* satisfy the bounds

2.32 $$\begin{aligned} \Vert \lambda \Vert _{l^2 X^s} + \Vert (h,A)\Vert _{{{\varvec{ {\mathcal {E}}}}}^s}\lesssim \Vert \lambda _0\Vert _{H^s} + \Vert h_0\Vert _{{\textbf{Y}}_0^{s+2}} + \Vert A_0\Vert _{H^{s+1}}. \end{aligned}$$

In addition, the functions $(\lambda , g,A)$ satisfy the constraints (2.8), (2.10), (2.12) and (2.17).

Here the solution $\lambda$ satisfies in particular the expected bounds

$$\begin{aligned} \Vert \lambda \Vert _{C[0,1;H^s]} \lesssim \Vert \lambda _0\Vert _{H^s}. \end{aligned}$$

The spaces $l^2 X^s$ and ${{\varvec{ {\mathcal {E}}}}}^s$, defined in the next section, contain a more complete description of the full set of variables $\lambda ,h,A$, which includes both Sobolev regularity and local energy bounds.

In the above theorem, by well-posedness we mean a full Hadamard-type well-posedness, including the following properties:

(i) Existence of solutions $\lambda \in C[0,1;H^s]$, with the additional regularity properties (2.32).
(ii) Uniqueness in the same class.
(iii) Continuous dependence of solutions with respect to the initial data in the strong $H^s$ topology.
(iv) Weak Lipschitz dependence of solutions with respect to the initial data in the weaker $L^2$ topology.
(v) Energy bounds and propagation of higher regularity.

We conclude this section with several remarks concerning the result in Theorem 2.5:

#### Remark 2.5.1

(*The variable* $\lambda$ *vs* $\psi$) In our earlier paper [9] we have worked with $\psi$ as the main dynamic variable for the Schrödinger flow, and the full second fundamental form $\lambda$ was obtained from $\psi$ by solving an elliptic div-curl system derived from the Codazzi relations (2.10). Here we work directly with $\lambda$, because solving this elliptic system has issues at the $L^2$ level in two^1^ space dimensions. The downside is that the components of $\lambda$ are not independent, and are instead connected via the compatibility relations (2.10). Thus, these relations will have to be propagated dynamically.

#### Remark 2.5.2

(*Initial data sets*) The harmonic/Coulomb gauge condition at the initial time plays no role in Theorem 2.5, where smallness is assumed for both $\lambda _0$, $h_0$ and $A_0$. However, it is useful in order to connect Theorem 2.5 with the earlier statement in Theorems 1.2, 1.3.

## Function Spaces and Notations

The goal of this section is to define the function spaces where we aim to solve the (SMCF) system in the good gauge, given by (2.28). Both the spaces and the notation presented in this section are similar to those introduced in [20–22].

We begin with some constants. Let regularity index $s>d/2$ and $\delta >0$ be a small^2^ constant satisfying

$$\begin{aligned} 0<\delta \ll s-s_d. \end{aligned}$$

We then define the constant $\sigma _d$ depending on dimensions *d* as

3.1 $$\begin{aligned} \sigma _d=d/2-\delta . \end{aligned}$$

For a function *u*(*t*, *x*) or *u*(*x*), let ${\hat{u}}={{\mathcal {F}}}u$ and $\check{u}={{\mathcal {F}}}^{-1}u$ denote the Fourier transform and inverse Fourier transform in the spatial variable *x*, respectively. Fix a smooth radial function $\varphi :{{\mathbb {R}}}^d \rightarrow [0,1]$ supported in $\{x\in {{\mathbb {R}}}^d:|x|\leqq 2\}$ and equal to 1 in $\{x\in {{\mathbb {R}}}^d:|x|\leqq 1\}$, and for any $i\in {{\mathbb {Z}}}$, let

$$\begin{aligned} \varphi _i(x):=\varphi (x/2^i)-\varphi (x/2^{i-1}). \end{aligned}$$

We then have the spatial Littlewood–Paley decomposition,

$$\begin{aligned} \sum _{i=-\infty }^{\infty }P_i (D)=1, \quad \sum _{i=0}^{\infty }S_i (D)=1, \end{aligned}$$

where $P_i$ localizes to frequency $2^i$ for $i\in {{\mathbb {Z}}}$, i.e,

$$\begin{aligned} {{\mathcal {F}}}(P_i u)=\varphi _i(\xi ){\hat{u}}(\xi ), \end{aligned}$$

and

$$\begin{aligned} S_0(D)=\sum _{i\leqq 0}P_i(D),\quad S_i(D)=P_i(D),\ \textrm{for}\ i>0. \end{aligned}$$

For simplicity of notation, we set

$$\begin{aligned} u_j=S_j u,\quad u_{\leqq j}=\sum _{i=0}^j S_i u,\quad u_{\geqq j}=\sum _{i=j}^{\infty } S_i u. \end{aligned}$$

For each $j\in {{\mathbb {N}}}$, let ${{\mathcal {Q}}}_j$ denote a partition of ${{\mathbb {R}}}^d$ into cubes of side length $2^j$, and let $\{\chi _Q\}$ denote an associated partition of unity. For a translation-invariant Sobolev-type space *U*, set $l^p_j U$ to be the Banach space with associated norm

$$\begin{aligned} \Vert u\Vert _{l^p_j U}^p=\sum _{Q\in {{\mathcal {Q}}}_j}\Vert \chi _Q u\Vert _U^p \end{aligned}$$

with the obvious modification for $p=\infty$.

Next we define the $l^2X^s$ and $l^2N^s$ spaces, which will be used for the primary variable $\lambda$, respectively for the source term in the Schrödinger equation for $\lambda$. Following [20–22], we first define the *X*-norm as

$$\begin{aligned} \Vert u\Vert _{X}=\sup _{l \in {{\mathbb {N}}}} \sup _{Q\in {{\mathcal {Q}}}_l} 2^{-\frac{l}{2}}\Vert u\Vert _{L^2L^2([0,1]\times Q)}. \end{aligned}$$

Here and throughout, $L^pL^q$ represents $L^p_tL^q_x$. To measure the source term, we use an atomic space *N* satisfying $X=N^{*}$. A function *a* is an atom in *N* if there is a $j\geqq 0$ and a $Q\in {{\mathcal {Q}}}_j$ such that *a* is supported in $[0,1]\times Q$ and

$$\begin{aligned} \Vert a\Vert _{L^2([0,1]\times Q)}\lesssim 2^{-\frac{j}{2}}. \end{aligned}$$

Then we define *N* as linear combinations of the form

$$\begin{aligned} f=\sum _k c_k a_k,\ \ \sum _k|c_k|<\infty ,\ \ a_k\ \textrm{atom}, \end{aligned}$$

with norm

$$\begin{aligned} \Vert f\Vert _N=\inf \bigg \{\sum _k |c_k|: f=\sum _k c_k a_k,\ a_k\ \textrm{atoms}\bigg \}. \end{aligned}$$

For solutions which are localized to frequency $2^j$ with $j \geqq 0$, we will work in the space

$$\begin{aligned} X_j=2^{-\frac{j}{2}}X\cap L^{\infty }L^2, \end{aligned}$$

with norm

$$\begin{aligned} \Vert u\Vert _{X_j}=2^{\frac{j}{2}}\Vert u\Vert _X+\Vert u\Vert _{L^{\infty }L^2}. \end{aligned}$$

One way to assemble the $X_j$ norms is via the $X^s$ space

$$\begin{aligned} \Vert u\Vert _{X^s}^2=\sum _{j\geqq 0} 2^{2js}\Vert S_j u\Vert _{X_j}^2. \end{aligned}$$

But we will also add the $l^p$ spatial summation on the $2^j$ scale to $X_j$, in order to obtain the space $l^p_j X_j$ with norm

$$\begin{aligned} \Vert u\Vert _{l^p_j X_j} =\left( \sum _{Q\in {{\mathcal {Q}}}_j} \Vert \chi _Q u\Vert _{X_j}^p\right) ^{1/p}. \end{aligned}$$

We then define the space $l^p X^s$ by

$$\begin{aligned} \Vert u\Vert _{l^p X^s}^2=\sum _{j\geqq 0}2^{2js}\Vert S_j u\Vert _{l^p_j X_j}^2. \end{aligned}$$

For the solutions of Schrödinger equation in (2.28), we will be working primarily in $l^2 X^s$.

We analogously define

$$\begin{aligned} N_j=2^{\frac{j}{2}}N+L^1L^2, \end{aligned}$$

which has norm

$$\begin{aligned} \Vert f\Vert _{N_j}=\inf _{f=2^{\frac{j}{2}}f_1+f_2} \big (\Vert f_1\Vert _N+\Vert f_2\Vert _{L^1L^2}\big ), \end{aligned}$$

and

$$\begin{aligned} \Vert f\Vert _{l^pN^s}^2=\sum _{j\geqq 0}2^{2js}\Vert S_j f\Vert _{l^p_j N_j}^2. \end{aligned}$$

Here we shall be working primarily with $l^2N^s$.

We also note that for any *j*, we have

$$\begin{aligned} \sup _{Q\in {{\mathcal {Q}}}_j} 2^{-\frac{j}{2}}\Vert u\Vert _{L^2L^2([0,1]\times Q)}\leqq \Vert u\Vert _{X}, \end{aligned}$$

hence

$$\begin{aligned} \Vert u\Vert _N\lesssim 2^{j/2}\Vert u\Vert _{l^1_jL^2L^2}. \end{aligned}$$

This bound will come in handy at several places later on.

For the parabolic system (2.29), it is natural to work in spaces of the form $L^\infty H^s$. However, in order to obtain frequency envelope bounds it is more convenient to slightly strengthen this norm. Precisely, we define the $Z^{\sigma ,s}$ norm as

$$\begin{aligned} \Vert h\Vert _{Z^{\sigma ,s}}^2 =\Vert |D|^\sigma S_0 h\Vert _{L^\infty L^2}^2+\sum _{j\geqq 1} 2^{2sj} \Vert S_j h\Vert _{L^\infty L^2}^2. \end{aligned}$$

Compared to $L^\infty H^s$, here we just commute the $L^\infty _t$ and $l^2$ frequency summation. For simplicity of notation, we denote $Z^s:=Z^{0,s}$. In particular we have

$$\begin{aligned} Z^s \subset L^\infty H^s. \end{aligned}$$

With these notations, we will seek the solution (*h*, *A*) to the parabolic system (2.29) in the space ${{\mathcal {E}}}^s$ defined by

$$\begin{aligned} \Vert (h,A)\Vert _{{\mathcal {E}}^s}=\Vert h\Vert _{Z^{\sigma _d,s+2}}+\Vert A\Vert _{Z^{s+1}}. \end{aligned}$$

Correspondingly, at fixed time we define the space ${{\mathcal {H}}}^s$ as

$$\begin{aligned} \Vert (h,A)\Vert _{{{\mathcal {H}}}^s}=\Vert |D|^{\sigma _d}h\Vert _{H^{s+2-\sigma _d}}+\Vert A\Vert _{H^{s+1}}. \end{aligned}$$

In addition to the above standard norms, for the study of the Schrödinger equation for $\lambda$ we will also need to control a stronger norm ${\textbf{Y}}^{s+2}$ for the metric $h=g-I_d$; this will be defined in what follows.

First, similarly to the $l^p_j X_j$ norms above, we also add the $l^p$ spatial summation on the $2^j$ scale to $Z_j$, in order to obtain the space $l^p Z^{\sigma ,s}$ with norm

$$\begin{aligned} \Vert h\Vert _{l^pZ^{\sigma ,s}}^2=\sum _{j\in {\mathbb {Z}}} 2^{2\sigma j^-+2sj^+} \Vert P_j h\Vert _{l^p_{|j|}Z_j}^2=\sum _{j\in {\mathbb {Z}}} 2^{2\sigma j^-+2sj^+} \Vert P_j h\Vert _{l^p_{|j|}L^\infty L^2}^2. \end{aligned}$$

Here we need to decompose the low frequency part, this allows us to obtain a estimate of *h* in $l^2 Z^{\sigma _d,s+2}$ in Proposition 5.4. Correspondingly, we will strengthen the $Z^{\sigma _d,s+2}$ norm of *h* to $l^2 Z^{\sigma _d,s+2}$.

More importantly, we will also introduce some additional structure which is associated to spatial scales larger than the frequency. Precisely, to measure the portion of *h* which is localized to frequency $2^j$, this time with $j\in {{\mathbb {Z}}}$, we decompose $P_j h$ as an atomic summation of components $h_{j,l}$ associated to spatial scales $2^l$ with $l\geqq |j|$, i.e.

$$\begin{aligned} P_jh=\sum _{l\geqq |j|}h_{j,l}. \end{aligned}$$

Then we define the $Y_j$-norm by

$$\begin{aligned} \Vert P_j h \Vert _{Y_j} =\inf _{P_jh=\sum _{l\geqq |j|}h_{j,l}} \sum _{l\geqq |j|} 2^{l-|j|} \Vert h_{j,l}\Vert _{l^1_lL^{\infty }L^2}. \end{aligned}$$

In the decomposition of $P_j h$ we may project and assume that all terms are also localized at frequency $2^j$. However in the definition of the $Y_j$ norms we make no such assumption.

Assembling together the dyadic pieces in an $l^2$ Besov fashion, we obtain the $Y^{s}$ space with norm given by

$$\begin{aligned} \Vert h\Vert _{Y^{s}}^2=\sum _{j\in {{\mathbb {Z}}}} 2^{2(\frac{d}{2}-\delta ) j^-+2sj^+}\Vert P_jh\Vert _{Y_j}^2. \end{aligned}$$

Then for *h*-equation in (2.28), we will be working primarily in ${\textbf{Y}}^{s+2}$, whose norm is defined by

$$\begin{aligned} \Vert h\Vert _{{\textbf{Y}}^{s+2}}=\Vert h\Vert _{l^2Z^{\sigma _d,s+2}}+\Vert h\Vert _{Y^{s+2}}. \end{aligned}$$

Collecting all the components defined above, for the parabolic system (2.29) we define the final ${{\varvec{ {\mathcal {E}}}}}^s$ norm as

$$\begin{aligned} \Vert (h,A)\Vert _{{{\varvec{ {\mathcal {E}}}}}^s}=\Vert h\Vert _{{\textbf{Y}}^{s+2}} +\Vert A\Vert _{Z^{s+1}}. \end{aligned}$$

At fixed time, we can remove the $L^\infty _t$ in ${\textbf{Y}}^{s+2}$ and ${{\varvec{ {\mathcal {E}}}}}^s$, and obtain the function spaces ${\textbf{Y}}_0^{s+2}$ and ${{\varvec{ {\mathcal {E}}}}}^s_0$ respectively. Precisely, we define the $Y_{0j}$ norm corresponding to $Y_j$ as

$$\begin{aligned} \Vert P_j h \Vert _{Y_{0j}} =\inf _{P_jh=\sum _{l\geqq |j|}h_{j,l}} \sum _{l\geqq |j|} 2^{l-|j|} \Vert h_{j,l}\Vert _{l^1_lL^2}. \end{aligned}$$

and obtain the $Y^s_0$ space with norm given by

$$\begin{aligned} \Vert h\Vert _{Y_0^{s}}^2=\sum _{j\in {{\mathbb {Z}}}} 2^{2(\frac{d}{2}-\delta ) j^-+2sj^+}\Vert P_jh\Vert _{Y_{0j}}^2. \end{aligned}$$

Then we obtain the space ${\textbf{Y}}^{s+2}_0$ with norm defined by

$$\begin{aligned} \Vert h\Vert _{{\textbf{Y}}^{s+2}_0}=\Vert |D|^{\sigma _d}h\Vert _{H^{s+2-\sigma _d}}+\Vert h\Vert _{Y^{s+2}_0}, \end{aligned}$$

and the space ${{\varvec{ {\mathcal {E}}}}}^s_0$ defined by

$$\begin{aligned} \Vert (h,A)\Vert _{{{\varvec{ {\mathcal {E}}}}}^s_0}=\Vert h\Vert _{{\textbf{Y}}^{s+2}_0} +\Vert A\Vert _{H^{s+1}}. \end{aligned}$$

Finally, to capture only the low frequency information in the $Y_0^s$ spaces, we introduce the $Y_0^{lo}$ norm, which is used in our main two dimensional result in Theorem 1.3:

$$\begin{aligned} \Vert h\Vert _{Y_0^{lo}}^2=\Vert P_{\geqq 0} h\Vert _{Y_{00} \cap L^\infty }+ \sum _{j < 0} 2^{2(\frac{d}{2}-\delta ) j^-}\Vert P_jh\Vert _{Y_{0j}}^2. \end{aligned}$$

Next, we define the frequency envelopes as in [20–22] which will be used in multilinear estimates. Consider a Sobolev-type space *U* for which we have

$$\begin{aligned} \Vert u\Vert _U^2=\sum _{k=0}^{\infty } \Vert S_k u\Vert _U^2. \end{aligned}$$

A frequency envelope for a function $u\in U$ is a positive $l^2$-sequence, $\{ a_j\}$, with

$$\begin{aligned} \Vert S_j u\Vert _U\leqq a_j. \end{aligned}$$

We shall only permit slowly varying frequency envelopes. Thus, we require $a_0\approx \Vert u\Vert _U$ and

3.2 $$\begin{aligned} a_j\leqq 2^{\delta |j-k|} a_k,\quad j,k\geqq 0,\ 0<\delta \ll s-d/2. \end{aligned}$$

The constant $\delta$ shall be chosen later and only depends on *s* and the dimension *d*. Such frequency envelopes always exist. For example, one may choose

3.3 $$\begin{aligned} a_j=2^{-\delta j}\Vert u\Vert _U+\max _k 2^{-\delta |j-k|} \Vert S_k u\Vert _U. \end{aligned}$$

Since we often use Littlewood–Paley decompositions, the next lemma is a convenient tool to see that our function spaces are invariant under the action of some standard classes of multipliers:

### Lemma 3.1

For any Schwartz function $f\in {{\mathcal {S}}}$, multiplier *m*(*D*) with $\Vert {{\mathcal {F}}}^{-1}(m(\xi ))\Vert _{L^1}<\infty$, and translation-invariant Sobolev-type space *U*, we have

$$\begin{aligned} \Vert m(D)f\Vert _{U}\lesssim \Vert {{\mathcal {F}}}^{-1}(m(\xi ))\Vert _{L^1}\Vert f\Vert _U. \end{aligned}$$

Finally, we state a Bernstein-type inequality and two estimates.

### Lemma 3.2

(Bernstein-type inequality, Lemma 3.2 [9]) For any $j,k\in {{\mathbb {Z}}}$ with $j+k\geqq 0$, $1\leqq r<\infty$ and $1\leqq q\leqq p\leqq \infty$, we have

$$\begin{aligned}&\Vert P_k f\Vert _{l^r_jL^p}\lesssim 2^{kd(\frac{1}{q}-\frac{1}{p})}\Vert P_k f\Vert _{l^r_jL^q}. \end{aligned}$$

### Proposition 3.3

(Algebra property) For any $f,\ g\in Y^{lo}_0$ we have

3.4 $$\begin{aligned} \Vert fg\Vert _{Y^{lo}_0}\lesssim \Vert f\Vert _{Y^{lo}_0}\Vert g\Vert _{Y^{lo}_0}. \end{aligned}$$

### Proof

We first note that by Bernstein’s inequality we have $Y^{lo}_0 \subset L^\infty$. Then for the high-low and low-high interactions we can estimate

$$\begin{aligned}&\Vert P_j (P_j f P_{<j} g)\Vert _{Y_{0j}}+\Vert P_j (P_{<j} f P_{j} g)\Vert _{Y_{0j}}\\ {}&\quad \lesssim \Vert P_j f\Vert _{Y_{0j}} \Vert P_{<j}g\Vert _{L^\infty }+\Vert P_{<j} f\Vert _{L^\infty } \Vert P_{j}g\Vert _{Y_{0j}}. \end{aligned}$$

For the high-high interactions, we have

$$\begin{aligned}&2^{(\frac{d}{2}-\delta )j^-}\Vert P_j \Big (\sum _{0>l>j}P_l f P_l g+P_{\geqq 0}f P_{\geqq 0}g\Big )\Vert _{Y_{0j}}\\&\quad \lesssim 2^{(d-\delta )j^-}\Big (\sum _{0>l>j}\Vert P_j (P_l f P_l g)\Vert _{l^1_{|j|}L^1}+\Vert P_{\geqq 0}f P_{\geqq 0}g\Vert _{l^1_{|j|}L^1}\Big )\\&\quad \lesssim \sum _{0>l>j} 2^{(d-\delta )(j^--l)} 2^{(d-\delta )l}\Vert P_l f\Vert _{L^2} \Vert P_l g\Vert _{L^2}+2^{(d-\delta )j^-}\Vert P_{\geqq 0}f\Vert _{L^2}\Vert P_{\geqq 0}g\Vert _{L^2}\\&\quad \lesssim \sum _{0>l>j} 2^{(d-\delta )(j^--l)} 2^{(d-\delta )l}\Vert P_l f\Vert _{Y_{0j}} \Vert P_l g\Vert _{Y_{0j}}+2^{(d-\delta )j^-}\Vert P_{\geqq 0}f\Vert _{Y_{00}}\Vert P_{\geqq 0}g\Vert _{Y_{00}}. \end{aligned}$$

These two bounds imply that

$$\begin{aligned} \Vert P_{<0}(fg)\Vert _{Y^{lo}_0}\lesssim \Vert f\Vert _{Y^{lo}_0}\Vert g\Vert _{Y^{lo}_0}. \end{aligned}$$

For the high-frequency part $P_{\geqq 0}(fg)$, we bound its $L^\infty$ norm by

$$\begin{aligned} \Vert P_{\geqq 0}(fg)\Vert _{L^\infty } \lesssim \Vert f\Vert _{L^\infty }\Vert g\Vert _{L^\infty }\lesssim \Vert f\Vert _{Y^{lo}_0}\Vert g\Vert _{Y^{lo}_0}. \end{aligned}$$

To bound its $Y_{00}$ norm, we further decompose it as

$$\begin{aligned} P_{\geqq 0}(fg)=P_{\geqq 0}(P_{\geqq 0}f \cdot g)+P_{\geqq 0}(P_{<0}f\cdot P_{\geqq 0}g)+P_{\geqq 0}(P_{<0}f\cdot P_{[-3,-1]}g). \end{aligned}$$

The first term is bounded by

$$\begin{aligned} \Vert P_{\geqq 0}(P_{\geqq 0}f \cdot g)\Vert _{Y_{00}} \lesssim \Vert P_{\geqq 0}f\Vert _{Y_{00}} \Vert g\Vert _{L^\infty }\lesssim \Vert f\Vert _{Y^{lo}_0}\Vert g\Vert _{Y^{lo}_0}. \end{aligned}$$

The second term is bounded similarly. We bound the last term by

$$\begin{aligned} \Vert P_{\geqq 0}(P_{< 0}f \cdot P_{[-3,-1]} g)\Vert _{Y_{00}} \lesssim \Vert P_{< 0}f \Vert _{L^\infty }\Vert P_{[-3,-1]}g\Vert _{Y_{00}} \lesssim \Vert f\Vert _{Y^{lo}_0}\Vert g\Vert _{Y^{lo}_0}. \end{aligned}$$

This completes the bound for high frequency part, and thus the proof of the proposition. $\square$

### Lemma 3.4

For any Schwartz function *f*, $j\in {{\mathbb {N}}}$ and $1\leqq r\leqq \infty$, we have

3.5 $$\begin{aligned}{} & {} \Vert e^{t\Delta }f\Vert _{l^r_jL^\infty _tL^2}\lesssim \Vert f\Vert _{l^r_j L^2}, \end{aligned}$$

3.6 $$\begin{aligned}{} & {} \Vert \int _0^t e^{(t-s)\Delta } S_j f\ \textrm{d}s\Vert _{l^r_j L^\infty L^2}\lesssim 2^{-2j^+}\Vert S_j f\Vert _{l^r_jL^\infty L^2}. \end{aligned}$$

### Proof

We use the heat kernel

$$\begin{aligned} K(t,x) = (4\pi t)^{-\frac{d}{2}} e^{-\frac{x^2}{4t}} \end{aligned}$$

which we decompose with respect to cubes $Q \in {{\mathcal {Q}}}_j$. Then from the corresponding decomposition

$$\begin{aligned} e^{t\Delta } f = \sum _{Q \in {{\mathcal {Q}}}_j} (\chi _Q(x) K(t,x)) *_x f \end{aligned}$$

we obtain

$$\begin{aligned} \Vert e^{t\Delta }f\Vert _{l^r_jL^\infty _tL^2} \lesssim \Vert K\Vert _{l^1_j L^\infty _t L^1_x} \Vert f\Vert _{L^2} \end{aligned}$$

Since $t \in [0,1]$ and $r \geqq 0$, we can use the exponential off-diagonal decay for *K* on the unit scale to conclude that

$$\begin{aligned} \Vert K\Vert _{l^1_j L^\infty _t L^1_x} \lesssim 1, \end{aligned}$$

and thus (3.5) follows.

For the second bound, we separate the low frequencies and use the kernel $K_0$ for $S_0e^{(t-s)\Delta }$ with a similar cube decomposition to estimate

$$\begin{aligned} \Vert \int _0^t e^{(t-s)\Delta } S_0 f\ \textrm{d}s\Vert _{l^r_0 L^\infty L^2} \lesssim \Vert K_0 \Vert _{l^1_j L^1_{t,x}} \Vert f\Vert _{l^r_0 L^\infty L^2} \end{aligned}$$

where the $K_0$ norm is easily estimates using the rapid kernel decay on the unit scale.

Similarly, for high frequencies $j > 0$ we use the kernel $K_j$ for $S_j e^{(t-s)\Delta }$ with a similar cube decomposition to estimate

$$\begin{aligned} \Vert \int _0^t e^{(t-s)\Delta } S_j f\ \textrm{d}s\Vert _{l^r_0 L^\infty L^2} \lesssim \Vert K_j \Vert _{l^1_j L^1_{t,x}} \Vert f\Vert _{l^r_0 L^\infty L^2} \end{aligned}$$

For fixed *t* we use the exponential symbol decay to obtain

$$\begin{aligned} \Vert K_j \Vert _{l^1_j L^1_{x}} \lesssim e^{-c 2^{2j} t}, \end{aligned}$$

and now the time integration yields the desired $2^{-2j}$ decay. This concludes the proof of (3.6). $\square$

## The Initial Data

Our evolution begins at time $t = 0$, where we need to make a good gauge choice for the initial submanifold $\Sigma _0$. This has two components,

(i) a good set of coordinates on $\Sigma _0$, namely the global harmonic coordinates, represented via the map $F : {{\mathbb {R}}}^d \rightarrow {{\mathbb {R}}}^{d+2}$.
(ii) a good orthonormal frame in $N \Sigma _0$, where we will use the Coulomb gauge.

Once this is done, we have the frame in the tangent space and the frame *m* in the normal bundle. In turn, as described in Section 2, these generate the metric *g*, the second fundamental form $\lambda$ with trace $\psi$ and the connection *A*, all at the initial time $t = 0$.

We will first carry out the construction of the global harmonic coordinates, and use them to prove bounds for the parametrization *F* and for the metric $g_0 = I_d + h_0$. Then we introduce the Coulomb gauge, which in turn determines $\lambda _0$ and $A_0$.

The final objective of this section will be to describe the regularity and size of $(\lambda _0,g_0,A_0)$, and thus justify the smallness condition (2.31) for the Schrödinger-Parabolic system(2.28)–(2.29). The main result of this section is stated below in Proposition 4.1 for dimensions $d\geqq 3$ and Proposition 4.2 for dimension 2, respectively.

In order to state the following propositions, we define some notations. Let $F:{{\mathbb {R}}}^d_x \rightarrow ({{\mathbb {R}}}^{d+2},g_{{{\mathbb {R}}}^{d+2}})$ be an immersion with induced metric *g*(*x*). For any change of coordinate $y=x+\phi (x)$, we denote

$$\begin{aligned} {\tilde{F}}(y)=F(x(y)), \end{aligned}$$

and its induced metric by ${{\tilde{g}}}_{\alpha {\beta }}(y)=\langle \partial _{y_\alpha }{{\tilde{F}}},\partial _{y_{\beta }}{{\tilde{F}}}\rangle$. We also denote its Christoffel symbol as ${\tilde{\Gamma }}$ and ${{\tilde{h}}}(y)={{\tilde{g}}}(y)-I_d$. The main results are summarized as follows:

### Proposition 4.1

(Harmonic coordinates and initial data in dimensions $d\geqq 3$) Let $d\geqq 3$, $s>\frac{d}{2}$. Let $F:({{\mathbb {R}}}^d_x,g)\rightarrow ({{\mathbb {R}}}^{d+2},g_{{{\mathbb {R}}}^{d+2}})$ be an immersion with induced metric $g=I_d+h$. Assume that the metric *h* and the mean curvature ${\textbf{H}}$ satisfy the smallness conditions

4.1 $$\begin{aligned} \Vert |D|^{\sigma _d} h\Vert _{H^{s+1-{\sigma _d}}}\leqq \epsilon _0,\quad \Vert {\textbf{H}}\Vert _{H^s}\leqq \epsilon _0. \end{aligned}$$

Then there exists a unique change of coordinates $y=x+\phi (x)$ with $\lim _{x\rightarrow \infty }\phi (x)=0$ and $\nabla \phi$ uniformly small, such that the new coordinates $\{y_1,\cdots ,y_d\}$ are global harmonic coordinates. Moreover, we have the bound

4.2 $$\begin{aligned} \Vert |D|^{\sigma _d} \nabla \phi \Vert _{H^{s+1-{\sigma _d}}} \lesssim \Vert |D|^{\sigma _d} h\Vert _{H^{s+1-{\sigma _d}}} \end{aligned}$$

and, in the new coordinates $\{y_1,\cdots ,y_d\}$, for the metric and mean curvature we have

4.3 $$\begin{aligned} \Vert |D_y|^{\sigma _d}{{\tilde{h}}}\Vert _{H^{s+1-\sigma _d}(\textrm{d}y)}+\Vert {\textbf{H}}\Vert _{H^{s}(\textrm{d}y)}\lesssim \epsilon _0. \end{aligned}$$

In addition, under the harmonic coordinate condition (2.23) for *g*, respectively the Coulomb gauge (2.18) for *A*, we have the following bounds for complex second fundamental form $\lambda$, metric $h = g - I_d$ and *A*:

4.4 $$\begin{aligned} \Vert \lambda \Vert _{H^s}+\Vert h\Vert _{{\textbf{Y}}_0^{s+2}} +\Vert A \Vert _{H^{s+1}} \lesssim \epsilon _0. \end{aligned}$$

Compared to the above higher dimensions cases, in dimensions 2 we would work in a smaller function space.

### Proposition 4.2

(Harmonic coordinates and initial data in dimension 2) Let $d= 2$, $s>\frac{d}{2}$, and $\sigma _d$ be as in (3.1). Let $F:({{\mathbb {R}}}^d_x,g)\rightarrow ({{\mathbb {R}}}^{d+2},g_{{{\mathbb {R}}}^{d+2}})$ be an immersion with induced metric $g=I_d+h$. Assume that the metric *h* and mean curvature ${\textbf{H}}$ satisfy the smallness conditions

4.5 $$\begin{aligned} \Vert |D|^{\sigma _d} h\Vert _{H^{s+1-{\sigma _d}}}\leqq \epsilon _0,\quad \Vert h\Vert _{Y^{lo}_0}\leqq \epsilon _0, \quad \Vert {\textbf{H}}\Vert _{H^s}\leqq \epsilon _0. \end{aligned}$$

Then there exists a change of coordinates $y=x+\phi (x)$, with $\nabla \phi$ uniformly small and with $\lim _{x\rightarrow \infty }\nabla \phi (x)=0$, unique modulo constants, such that the new coordinates $\{y_1,\cdots ,y_d\}$ are global harmonic coordinates. Moreover, we have the bound

4.6 $$\begin{aligned} \Vert |D|^{\sigma _d}\nabla \phi \Vert _{H^{s+1-\sigma _d}}\lesssim \Vert |D|^{\sigma _d} h\Vert _{H^{s+1-\sigma _d}}, \end{aligned}$$

and, in the new coordinates $\{y_1,\cdots ,y_d\}$, for the metric and mean curvature we have

4.7 $$\begin{aligned} \Vert |D_y|^{\sigma _d}{{\tilde{h}}}\Vert _{H^{s+1-\sigma _d}(\textrm{d}y)}+\Vert {{\tilde{h}}}\Vert _{Y^{lo}_0}+\Vert {\textbf{H}}\Vert _{H^{s}(\textrm{d}y)}\lesssim \epsilon _0\,. \end{aligned}$$

In addition, under the harmonic coordinate condition (2.23) for *g*, respectively the Coulomb gauge (2.18) for *A*, we have the following bounds for complex second fundamental form $\lambda$, metric $h = g - I_d$ and *A*:

4.8 $$\begin{aligned} \Vert \lambda \Vert _{H^s}+\Vert h\Vert _{{\textbf{Y}}_0^{s+2}} +\Vert A \Vert _{H^{s+1}} \lesssim \epsilon _0. \end{aligned}$$

We remark that the bounds (4.4) respectively (4.8) are the only way the harmonic/Coulomb gauge condition at $t = 0$ enters this paper. Later, in the study of the parabolic system (2.29), we simply assume that the initial data $(\lambda _0,h_0,A_0)$ satisfies the above smallness condition.

Of the three components of the initial data, $\lambda _0$ may be thought of as the fundamental one. Indeed, the initial data $(g_0,A_0)$ for the heat flow (2.29) is determined by $\lambda _0$ via the harmonic coordinate condition (2.23) for *g*, respectively the Coulomb gauge (2.18) for *A*, which yield the elliptic equations in Lemmas 2.3 and 2.1. This was the point of view adopted in our previous paper [9] in high dimension, and it largely applies here as well. The only exception to this is in two space dimensions, where we a-priori make an additional low frequency assumption on the metric *g*, namely the $Y_0^{lo}$ bound, which cannot be recovered from the $\lambda _0$ bounds.

### Global Harmonic Coordinates

Here we make a change of coordinates to gain the harmonic coordinates, and then prove that in the new coordinates, the metric *h* and mean curvature ${\textbf{H}}$ are also small.

*Step 1: Solve the* $\phi$ *equation and prove the bounds* (4.2) *and* (4.6). To obtain harmonic coordinates, we start with the bound for metric

4.9 $$\begin{aligned} \Vert |D|^{\sigma _d}h\Vert _{H^{s+1-\sigma _d}}\lesssim \epsilon _0. \end{aligned}$$

We make a change of coordinates $x+\phi (x)=y$ with $\nabla \phi$ small such that the new coordinates are harmonic. Since the operator $\Delta _g$ does not depend on the coordinates, by (2.23) we have

$$\begin{aligned} \Delta _g (x+\phi (x))=0, \end{aligned}$$

which implies

4.10 $$\begin{aligned} \Delta _g \phi _\gamma = g^{\alpha {\beta }}\Gamma _{\alpha {\beta }}^\gamma , \end{aligned}$$

and which we write schematically in the form

$$\begin{aligned} \Delta \phi =h\nabla ^2 \phi +g\nabla h \nabla \phi +g\nabla h. \end{aligned}$$

Since the leading order term in the right hand side is $\nabla h$, by the assumption on the metric $|D|^{\sigma _d}h \in H^{s+1-\sigma _d}$ we will work in the space

$$\begin{aligned} \{ \phi : \Vert |D|^{1+\sigma _d}\phi \Vert _{H^{s+1-\sigma _d}}<\infty \} \end{aligned}$$

Then by Sobolev embeddings and the smallness of *h* we can uniquely solve the equation (4.10) in this space using the contraction principle, obtaining a solution $\phi$ which satisfies the bound

4.11 $$\begin{aligned} \Vert |D|^{\sigma _d} \nabla \phi \Vert _{H^{s+1-\sigma _d}}\lesssim \Vert |D|^{\sigma _d}h\Vert _{H^{s+1-\sigma _d}}\lesssim \epsilon _0. \end{aligned}$$

which is exactly (4.2) and (4.6) in Theorem 4.1, respectively Theorem 4.2.

*Step 2: Prove the bounds* (4.3) *and* (4.7) *for* ${{\tilde{h}}}$ *and* ${\textbf{H}}$ *in Sobolev spaces.* First we prove that the desired ${{\tilde{h}}}$ bound holds in the *x*-coordinates,

4.12 $$\begin{aligned} \Vert |D_x|^{\sigma _d}{{{\tilde{h}}}}(y(x))\Vert _{H^{s+1-\sigma _d}(\textrm{d}x)}\lesssim \Vert |D|^{\sigma _d} h\Vert _{H^{s+1-\sigma _d}(\textrm{d}x)}. \end{aligned}$$

By the above change of coordinate and (4.2) we have $\frac{\partial x}{\partial y}=I_d+{{\mathcal {P}}}(x)$ where ${{\mathcal {P}}}$ is an algebraic function of $\nabla \phi$. Hence by algebra and Moser estimates we have

4.13 $$\begin{aligned} \Vert |D|^{\sigma _d} {{\mathcal {P}}}\Vert _{H^{s+1-\sigma _d}}\lesssim \Vert |D|^{\sigma _d} \nabla \phi \Vert _{H^{s+1-\sigma _d}}\lesssim \epsilon _0. \end{aligned}$$

Then the desired bound (4.12) follows from the relation

$$\begin{aligned} {\tilde{g}}_{\alpha {\beta }}(y(x))=g_{\mu \nu }(x)(\delta ^\mu _\alpha +{{\mathcal {P}}}^{\mu }_\alpha )(\delta ^\nu _{\beta }+{{\mathcal {P}}}^{\nu }_{\beta }), \end{aligned}$$

again by using algebra bounds in the same space.

In order to complete the proof of (4.3) and (4.7), we need to be able to transfer the Sobolev norms from the *x* to the *y* coordinates. For this we will apply the following lemma:

#### Lemma 4.3

Let the change of coordinates $x+\phi (x)=y$ be as in Proposition 4.1. Define the linear operator *T* as $T(f)(y)=f(x(y))$ for any function $f\in L^2(\textrm{d}x)$. Then we have

4.14 $$\begin{aligned}&\Vert T(f)(y)\Vert _{H^\sigma (\textrm{d}y)}\lesssim \Vert f(x)\Vert _{H^\sigma (\textrm{d}x)}, \qquad \sigma \in [0,s+1], \end{aligned}$$

4.15 $$\begin{aligned}&\Vert T(f)(y)\Vert _{\dot{H}^{\alpha }(\textrm{d}y)}\lesssim \Vert f(x)\Vert _{\dot{H}^{\alpha }(\textrm{d}x)},\qquad \alpha \in [0,\frac{d}{2}). \end{aligned}$$

#### Proof

The first bound is obtained from (4.13) and (4.2) using the same argument as in Lemma 8.5 in [9], It remains to prove the second bound (4.15).

By the smallness of $\phi$ (4.2) we have

$$\begin{aligned} \Vert T(f)(y)\Vert _{L^2(\textrm{d}y)}&\lesssim \Vert f(x) \sqrt{I+\partial _x\phi } \Vert _{L^2(\textrm{d}x)}\\&\lesssim (1+\Vert |D|^{1+\sigma _d}\Vert _{H^{s-\sigma _d}})^N\Vert f(x)\Vert _{L^2(\textrm{d}x)}\lesssim \Vert f(x)\Vert _{L^2(\textrm{d}x)}. \end{aligned}$$

Similarly, by (4.2) and (4.13) we also have

$$\begin{aligned} \Vert \partial _y T(f)(y)\Vert _{L^2(\textrm{d}y)}&\lesssim \Vert (1+{{\mathcal {P}}}) \partial _x f(x) \sqrt{I+\partial _x\phi } \Vert _{L^2(\textrm{d}x)} \lesssim \Vert \partial _x f(x)\Vert _{L^2(\textrm{d}x)}. \end{aligned}$$

Then by interpolation we obtain (4.15) for $\alpha \in [0,1]$. This suffices in dimension $d = 2$. In higher dimension, we inductively increase the range of $\alpha$ by differentiating. Precisely, for $\alpha > 1$ we have

$$\begin{aligned} \Vert T(f)(y)\Vert _{\dot{H}^{\alpha }(\textrm{d}y)} = \Vert \partial _y T(f)(y)\Vert _{\dot{H}^{\alpha -1}(\textrm{d}y)} \end{aligned}$$

Here

$$\begin{aligned} \partial _y T(f)(y) = T( (I+{{\mathcal {P}}}) \partial _x f) \end{aligned}$$

and, by (4.13),

$$\begin{aligned} \Vert (I+{{\mathcal {P}}}) \partial _x f\Vert _{\dot{H}^{\alpha -1}(\textrm{d}x)} \lesssim \Vert \partial _x f\Vert _{\dot{H}^{\alpha -1}(\textrm{d}x)} \end{aligned}$$

Hence we have reduced the $\dot{H}^\alpha$ bound to the $\dot{H}^{\alpha -1}$ bound. $\square$

Given this lemma, by (4.12), (4.14) with $\sigma = s+1$ and (4.15) we obtain

$$\begin{aligned} \Vert |D|^{\sigma _d} {{{\tilde{h}}}}\Vert _{H^{s+1-\sigma _d}(\textrm{d}y)}\lesssim \Vert |D|^{\sigma _d} h\Vert _{H^{s+1-\sigma _d}(\textrm{d}x)} \end{aligned}$$

Hence the ${{\tilde{h}}}$ bounds in (4.3) and (4.7) follow. Similarly, the ${\textbf{H}}$ bound is also directly transferred to the *y* coordinates by Lemma 4.3.

*Step 3: Prove bounds for* $\partial ^2 F$ *in harmonic coordinates.* While this bound was not explicitely stated in Propositions 4.1, 4.2, it will play an important role later in the proof of the bounds (4.4) and (4.8).

#### Lemma 4.4

Let $d\geqq 2$, $s>\frac{d}{2}$, and $F:({{\mathbb {R}}}^d,g)\rightarrow ({{\mathbb {R}}}^{d+2},g_{{{\mathbb {R}}}^{d+2}})$ be an immersion with metric $\Vert |D|^{\sigma _d}h\Vert _{H^{s+1-\sigma _d}}\lesssim \epsilon _0$ and mean curvature $\Vert {\textbf{H}}\Vert _{H^s}\lesssim \epsilon _0$ in some coordinates. Then we have

4.16 $$\begin{aligned} \Vert \partial ^2 F\Vert _{H^s}\lesssim \epsilon _0. \end{aligned}$$

We note that, as a corollary, it follows that we also have the bound

4.17 $$\begin{aligned} \Vert \nabla {{\tilde{h}}}\Vert _{H^s} \lesssim \epsilon _0. \end{aligned}$$

This bound in effect superseeds the ${{\tilde{h}}}$ bound in (4.3), (4.7), with one exception, namely in two dimensions at low frequency.

Another corollary of this is the corresponding bound for the second fundamental form ${\textbf{h}}$, namely

4.18 $$\begin{aligned} \Vert {\textbf{h}}\Vert _{H^s} \lesssim \epsilon _0. \end{aligned}$$

#### Proof of Proposition 4.4

By the smallness of $|D|^{\sigma _d}(g-I_d)$ and Sobolev embedding, we have

$$\begin{aligned} \Vert g^{\alpha {\beta }}\Gamma _{\alpha {\beta }}^\gamma \partial _\gamma F\Vert _{H^s}&\lesssim (1+\Vert |D|^{\sigma _d} h\Vert _{H^{s+1-\sigma _d}})\Vert |D|^{\sigma _d} h\Vert _{H^{s+1-\sigma _d}}(\Vert \partial _\gamma F\Vert _{L^\infty \cap {\dot{H}}^s})\\&\lesssim \epsilon _0 (\Vert g\Vert _{L^\infty }^{1/2}+\Vert \partial ^2 F\Vert _{H^s}) \lesssim \epsilon _0 (1+\Vert \partial ^2 F\Vert _{H^s}). \end{aligned}$$

Then we can bound $\partial ^2 F$ by

$$\begin{aligned} \Vert \partial ^2 F\Vert _{H^s}&= \Vert {\mathcal {R}} \Delta F\Vert _{H^s}\lesssim \Vert \Delta F\Vert _{H^s}\\&\lesssim \Vert \Delta _g F\Vert _{H^s}+\Vert h^{\alpha {\beta }}\partial ^2_{\alpha {\beta }}F\Vert _{H^s}+\Vert g^{\alpha {\beta }} \Gamma _{\alpha {\beta }}^\gamma \partial _\gamma F \Vert _{H^s}\\&\lesssim \Vert {\textbf{H}}\Vert _{H^s}+\epsilon _0(1+ \Vert \partial ^2 F\Vert _{H^s})\\&\lesssim \epsilon _0(1+ \Vert \partial ^2 F\Vert _{H^s}), \end{aligned}$$

which implies (4.16), and thus completes the proof of lemma. $\square$

*Step 4: Prove the* $Y_{0}^{lo}$ *bound for the metric* *h* *in* (4.7) *in two dimensions.* To transfer the $Y_0^{lo}$ bounds to ${{\tilde{h}}}$, our starting point is the estimate

$$\begin{aligned} \Vert h\Vert _{Y_0^{lo}} \lesssim \epsilon _0 \end{aligned}$$

Next we show that $\nabla \phi$ satisfies a similar bound,

4.19 $$\begin{aligned} \Vert \nabla \phi \Vert _{{{\tilde{Y}}}_0^{lo}} \lesssim \epsilon _0 \end{aligned}$$

#### Proof of (4.19)

We use the $\phi$-equations (4.10), which have the form

$$\begin{aligned} \Delta \phi =\nabla h+h\nabla ^2 \phi +g\nabla h \nabla \phi +h\nabla h. \end{aligned}$$

To get (4.19) via the contraction principle it suffices to estimate the right hand side above in order to prove that

$$\begin{aligned} \Vert \nabla \phi \Vert _{Y^{lo}_0}\lesssim \Vert h\Vert _{Y^{lo}_0}+\epsilon _0(\Vert h\Vert _{Y^{lo}_0}+\Vert \nabla \phi \Vert _{Y^{lo}_0})+\epsilon _0^2 \end{aligned}$$

First, we bound the $Y_j$ norm of $\nabla \phi$. For the $\nabla h$, we easily have

$$\begin{aligned} \Vert P_j\nabla ^{-1} \nabla h\Vert _{Y_j}\lesssim \Vert P_j h\Vert _{Y_j}, \end{aligned}$$

which is acceptable. We will next show how to bound the most umbalanced term $h\nabla ^2 \phi$; the rest of the terms are estimated similarly. For the high-low interactions $P_j h \nabla ^2 P_{<j} \phi$, by (4.11) we have

$$\begin{aligned} \Vert P_j \nabla ^{-1}(P_j h \nabla ^2 P_{<j}\phi )\Vert _{Y_{0j}}&\lesssim \Vert P_j h\Vert _{Y_{0j}} \Vert \nabla P_{<j}\phi \Vert _{L^\infty }\lesssim \epsilon _0\Vert P_j h\Vert _{Y_{0j}}. \end{aligned}$$

Similarly, for the low-high interactions $P_{<j} h \nabla ^2 P_j\phi$, by (4.9) we have

$$\begin{aligned} \Vert P_{j} \nabla ^{-1}(P_{<j} h \nabla ^2 P_j\phi )\Vert _{Y_{0j}}&\lesssim \Vert P_{<j} h\Vert _{L^\infty } \Vert \nabla P_{j}\phi \Vert _{Y_{0j}}\lesssim \epsilon _0 \Vert \nabla P_{j}\phi \Vert _{Y_{0j}}. \end{aligned}$$

Finally we consider the high-high interactions, $\sum _{l> j} P_j( P_{l} h \nabla ^2 P_l\phi )$. Here we use Bernstein’s inequality to obtain

$$\begin{aligned} 2^{(\frac{d}{2}-\delta )j}\Vert \sum _{l> j} \nabla ^{-1} P_j( P_{l} h \nabla ^2 P_l\phi )\Vert _{Y_{0j}}&\lesssim 2^{(\frac{d}{2}-1-\delta )j}\sum _{l>j}\Vert P_j( P_{l} h \nabla ^2 P_l\phi )\Vert _{l^1_{|j|}L^2}\\&\lesssim 2^{(d-1-\delta )j}\sum _{l>j}\Vert P_{l} h\Vert _{L^2}\Vert \nabla ^2 P_l\phi \Vert _{L^2}\\&\lesssim \sum _{l>j} 2^{(d-1-\delta )(j-l)} \Vert |D|^{\frac{d}{2}-\delta } P_l h\Vert _{L^2}\Vert |D|^\frac{d}{2} P_l \nabla \phi \Vert _{L^2}, \end{aligned}$$

which in view of the bound (4.11) gives

$$\begin{aligned} \Big (\sum _{j<0}2^{2(\frac{d}{2}-\delta )j^-}\Vert \sum _{l> j} \nabla ^{-1} P_j( P_{l} h \nabla ^2 P_l\phi )\Vert _{Y_{0j}}^2\Big )^{1/2}\lesssim \Vert |D|^{\sigma _d} h\Vert _{L^2} \Vert |D|^{\frac{d}{2}} \nabla \phi \Vert _{L^2}\lesssim \epsilon _0^2. \end{aligned}$$

Secondly, we bound the $Y_{00}\cap L^\infty$ norm for the high frequency part $P_{\geqq 0} \nabla \phi$. The $L^\infty$ bound follows from the $H^s$ bound for $\phi$ and Sobolev embeddings. It remains to estimate its $Y_{00}$ norm. Since the operator $P_{>0} \nabla ^{-1} \nabla$ has the kernel localized to the unit spatial scale, we have

$$\begin{aligned} \Vert P_{\geqq 0}\nabla ^{-1}\nabla h\Vert _{Y_{00}}\lesssim \Vert P_{\geqq 0}\Vert _{Y_{00}}. \end{aligned}$$

Here we also only discuss the term $h\nabla ^2\phi$; the contributions of the other terms are estimated similarly. We first divide this term as

$$\begin{aligned} P_{\geqq 0}\nabla ^{-1}(P_{\geqq 0 }h \nabla ^2 \phi )+P_{\geqq 0}\nabla ^{-1}(P_{< 0 }h \nabla ^2 P_{\geqq 0} \phi )+P_{\geqq 0}\nabla ^{-1}(P_{< 0 }h \nabla ^2 P_{[-3,-1]}\phi ). \end{aligned}$$

For the first term, we directly have

$$\begin{aligned} \Vert P_{\geqq 0}\nabla ^{-1}(P_{\geqq 0 }h \nabla ^2 \phi )\Vert _{Y_{00}}\lesssim \Vert P_{\geqq 0}h\Vert _{Y_{00}}\Vert \nabla ^2 \phi \Vert _{L^\infty }\lesssim \epsilon _0\Vert P_{\geqq 0}h\Vert _{Y_{00}}. \end{aligned}$$

The second term, we further divide it as

$$\begin{aligned} P_{\geqq 0}\nabla ^{-1}(P_{< 0 }h \nabla ^2 P_{\geqq 0} \phi )=P_{\geqq 0}{\mathcal {R}} (P_{< 0 }h \nabla P_{\geqq 0} \phi )+P_{\geqq 0}\nabla ^{-1}(\nabla P_{< 0 }h \nabla P_{\geqq 0} \phi ), \end{aligned}$$

where ${\mathcal {R}}$ is Riesz transform. Then we bound this by

$$\begin{aligned} \Vert P_{\geqq 0}\nabla ^{-1}(P_{< 0 }h \nabla ^2 P_{\geqq 0} \phi )\Vert _{Y_{00}}\lesssim \Vert P_{<0}h\Vert _{L^\infty }\Vert P_{\geqq 0}\nabla \phi \Vert _{Y_{00}}\lesssim \epsilon _0 \Vert P_{\geqq 0}\nabla \phi \Vert _{Y_{00}}. \end{aligned}$$

Finally, we bound the last term by

$$\begin{aligned} \Vert P_{\geqq 0}\nabla ^{-1}(P_{< 0 }h \nabla ^2 P_{[-3,-1]} \phi )\Vert _{Y_{00}}\lesssim \Vert P_{<0}h\Vert _{L^\infty }\Vert P_{[-3,-1]}\nabla \phi \Vert _{Y_{00}}\lesssim \epsilon _0 \Vert P_{[-3,-1]}\nabla \phi \Vert _{Y^{lo}_0}. \end{aligned}$$

This concludes the proof of the $Y_{00}\cap L^\infty$ norm for $P_{\geqq 0}\nabla \phi$. $\square$

The new metric ${{\tilde{h}}}$ expressed in the *x* coordinates has the cubic polynomial form

$$\begin{aligned} {{\tilde{h}}} = P(h,\nabla \phi ). \end{aligned}$$

Using the algebra property (3.4) for $$Y^{lo}_0 $$ and (4.19), we conclude that

$$\begin{aligned} \Vert {{\tilde{h}}}\Vert _{{{\tilde{Y}}}^x_0} \lesssim \epsilon _0 \end{aligned}$$

It remains to switch this bound to the *y* coordinates, i.e. show that

4.20 $$\begin{aligned} \Vert {{\tilde{h}}}\Vert _{{{\tilde{Y}}}^x_0} \approx \Vert {{\tilde{h}}}\Vert _{{{\tilde{Y}}}^y_0} \end{aligned}$$

where the difficulty is that we need to use a Littlewood–Paley decomposition. We will circumvent this by using the following representation of $Y_0^{lo}$ functions:

#### Lemma 4.5

A function *f* is in $Y_0^{lo}$ iff it admits a representation

$$\begin{aligned} f = \sum _{j \leqq 0} f_j, \qquad f_j \in Y_{0j} \end{aligned}$$

so that the following norm is finite:

$$\begin{aligned} |\!|\!|(f_j)|\!|\!|^2 = \Vert f_0\Vert _{Y_{00} \cap L^\infty } + \sum _{j < 0} 2^{2(1-\delta )j} \left( \Vert f_j\Vert _{Y_{0j}}^2 + 2^{-2j} \Vert \nabla f_j\Vert _{Y_{0j}}^2+ 2^{-4 j} \Vert \nabla ^2 f_j\Vert _{Y_{0j}}^2\right) \end{aligned}$$

Further, we have

$$\begin{aligned} \Vert f\Vert _{Y_0^{lo}} \approx \inf \{ |\!|\!|(f_j)||\!|\!|; f =\sum _{j \leqq 0} f_j \} \end{aligned}$$

Since by Sobolev embeddings $\phi$ is small in $C^2$, the triple norms are easily seen to be equivalent in the *x* and the *y* coordinates, therefore the relation (4.20) follows. It remains to prove the Lemma.

#### Proof

In one direction, we directly see that the decomposition

$$\begin{aligned} f_0 = P_{\geqq 0} f, \qquad f_j = P_j f, \quad j < 0 \end{aligned}$$

yields

$$\begin{aligned} \Vert f\Vert _{{{\tilde{Y}}}_0} \approx |\!|\!|(f_j)|\!|\!|\end{aligned}$$

Conversely, if $f = \sum f_j$, then we need to show that

4.21 $$\begin{aligned} \Vert f\Vert _{{{\tilde{Y}}}_0} \lesssim |\!|\!|(f_j)|\!|\!|\end{aligned}$$

For this we estimate for $k < 0$

$$\begin{aligned} \begin{aligned} \Vert P_k f\Vert _{Y_{0k}}&\lesssim \ \Vert P_k f_0\Vert _{Y_{0k}} + \sum _{j< 0} \Vert P_k f_j\Vert _{Y_{0k}} \\&\lesssim \ 2^k \Vert f_0\Vert _{Y_{00}}+ \sum _{j < 0} 2^{-|j-k|} (\Vert f_j\Vert _{Y_j} + 2^{-2j} \Vert \nabla ^2 f_j\Vert _{Y_j}) \end{aligned} \end{aligned}$$

Due to the off-diagonal decay, this implies (4.21). $\square$

### The Initial Data $(\lambda _0,h_0,A_0)$

These are determined by the initial manifold $\Sigma _0$ given a gauge choice, which consists of choosing (i) a good set of coordinates on $\Sigma _0$, namely the harmonic coordinates, and (ii) a good orthonormal frame in $N \Sigma _0$, where we will use the Coulomb gauge.

In the previous subsection we have discussed the construction of harmonic coordinates and proved the Sobolev bound (4.17) for $h_0$. Here we begin by constructing a Coulomb frame in the normal bundle. Then we can define $\lambda _0$ and $A_0$ and directly prove $H^s$ bounds for them.

However, it turns out that the $H^s$ bounds tell only part of the story for $h_0$ and $A_0$, by treating them as linear objects. Instead, in our chosen gauge both $h_0$ and $A_0$ should be seen as quadratic objects, via the equations (2.23), respectively (2.18). In the last part of the section we use these equations to improve the bounds for both $h_0$ and $A_0$.

*Step 1: The Coulomb frame in* $N \Sigma _0$ *and the* $H^s$ *bound for* $\lambda$ *and* *A*. To obtain the Coulomb gauge, we choose ${\tilde{\nu }}$ constant uniformly transversal to $T \Sigma _0$; such a ${{\tilde{\nu }}}$ exists because, by Sobolev embeddings, $\partial _x F$ has a small variation in $L^\infty$. Projecting ${\tilde{\nu }}$ on the normal bundle $N \Sigma _0$ and normalizing we obtain a normalized section ${\tilde{\nu }}_1$ of the normal bundle with the same regularity as $\partial F$. Then we choose ${\tilde{\nu }}_2$ in $N \Sigma _0$ perpendicular to ${\tilde{\nu }}_1$. We obtain the orthonormal frame $({\tilde{\nu }}_1,{\tilde{\nu }}_2)$ in $N \Sigma _0$, which again has the same regularity and bounds as $\partial _x F$, namely (see Lemma 4.4)

4.22 $$\begin{aligned} \Vert \partial {{\tilde{\nu }}}_j\Vert _{H^s} \lesssim \epsilon _0. \end{aligned}$$

This in particular implies that the associated connection ${{\tilde{A}}}$ also satisfies

4.23 $$\begin{aligned} \Vert {{\tilde{A}}}\Vert _{H^s} \lesssim \epsilon _0. \end{aligned}$$

Then we rotate the frame to get a Coulomb frame $(\nu _1,\nu _2)$, i.e. where the Coulomb gauge condition $\nabla ^\alpha A_\alpha =0$ is satisfied. In our complex notation, this corresponds to

$$\begin{aligned} \nu _1 + i \nu _2 = e^{ib} ({{\tilde{\nu }}}_1+ i{{\tilde{\nu }}}_2), \qquad A_j = {{\tilde{A}}}_j -\partial _j b, \end{aligned}$$

where the rotation angle *b* must solve

$$\begin{aligned} \Delta _g b = \nabla ^\alpha {{\tilde{A}}}_\alpha . \end{aligned}$$

This is an elliptic equation, where the metric $g_0= I_d+h_0$ satisfies (4.17). Using the variational formulation at the $H^1$ level and then perturbative analysis at higher regularity, the solution is easily seen to satisfy

$$\begin{aligned} \Vert \partial b\Vert _{H^s} \lesssim \Vert A\Vert _{H^s} \end{aligned}$$

It directly follows that $\nu _1, \nu _2$ and *A* also satisfy the bounds in (4.22), (4.23),

4.24 $$\begin{aligned} \Vert \partial \nu _j\Vert _{H^s} + \Vert A\Vert _{H^s} \lesssim \epsilon _0. \end{aligned}$$

Projecting the second fundamental form ${\textbf{h}}$ and the mean curvature ${\textbf{H}}$ on the Coulomb frame as in Section 2.2 we obtain the complex second fundamental form $\lambda$ and the complex mean curvature $\psi$. In view of (4.1), (4.5) and (4.18) both of them have the same regularity,

$$\begin{aligned} \Vert \lambda \Vert _{H^s} + \Vert \psi \Vert _{H^s} \lesssim \epsilon _0. \end{aligned}$$

*Step 2: Prove the bounds in* (4.4) *and* (4.8) *for the metric* *h*. For this we rely on the equation (2.24). The main result is as follows:

#### Lemma 4.6

Let $d\geqq 2$, $s>\frac{d}{2}$ and $\sigma _d$ be as in (3.1). Assume that *h* is a solution of (2.24) satisfying

$$\begin{aligned} \Vert |D|^{\sigma _d}h\Vert _{H^{s+1-\sigma _d}}\leqq \epsilon _0, \qquad \Vert \lambda \Vert _{H^s}\leqq \epsilon _0. \end{aligned}$$

Then for $d\geqq 3$ we have

4.25 $$\begin{aligned} \Vert \partial h\Vert _{H^{s+1}}+\Vert h\Vert _{Y_0^{s+2}}\lesssim \epsilon _0. \end{aligned}$$

Under the additional assumption

4.26 $$\begin{aligned} \Vert h\Vert _{Y^{lo}_0}\leqq \epsilon _0, \end{aligned}$$

in dimension $d=2$ we have

4.27 $$\begin{aligned} \Vert |D|^{\sigma _d}h\Vert _{H^{s+2-\sigma _d}}+\Vert P_{\geqq 0} h\Vert _{Y_0^{s+2}}\lesssim \epsilon _0. \end{aligned}$$

Here we remark on the key difference between dimensions two and higher. In higher dimensions $d \geqq 3$, *h* may be seen as the unique small solution for the equation (2.24). But in two dimensions, we merely use (2.24) to improve the high frequency bound for *h*. At low frequency this no longer works, and instead we use the low frequency bounds on the initial metric $h_0$ as an assumption in our main result. We note that the assumption (4.26) in the two dimensional case could be avoided, at the expense of a considerably longer proof.

#### Proof

By (2.24), it suffices to write the equation for *h* in the shorter form

$$\begin{aligned} \Delta h=h\nabla ^2 h+\nabla h\nabla h+\lambda ^2. \end{aligned}$$

From this and Sobolev embedding, we easily have

$$\begin{aligned} \Vert P_{\geqq 0}\Delta h\Vert _{H^s}&\lesssim \ \Vert |D|^{\sigma _d}h\Vert _{H^{s+1-\sigma _d}}\Vert |D|^{\sigma _d}h\Vert _{H^{s+2-\sigma _d}}+\Vert \partial _x h\Vert _{H^{s}}^2+\Vert \lambda \Vert _{H^s}^2\\&\lesssim \ \epsilon _0 \Vert P_{\geqq 0}|D|^{\sigma _d}h\Vert _{H^{s+2-\sigma _d}}+\epsilon _0^2. \end{aligned}$$

This implies that

4.28 $$\begin{aligned} \Vert |D|^{\sigma _d}h\Vert _{H^{s+2-\sigma _d}}\lesssim \epsilon _0. \end{aligned}$$

In dimension three and higher, a similar argument applies in order to improve the low frequency bound. This argument is already in [9], and we do not repeat it here.

Next, we bound the $Y^{s+2}_0$ norm of *h*. For the low-frequency part, we only need to consider the higher dimensional case $d \geqq 3$, as in the case $d = 2$ the low frequency bound is assumed in Theorem 1.3. We bound the high-low or low-high interactions by

$$\begin{aligned} \begin{aligned} \Vert \Delta ^{-1}P_j (P_{\leqq j-3}h \nabla ^2 h)\Vert _{Y_j}&\lesssim \ 2^{-2j}\sum _{l\geqq |j|}2^{l-|j|}\Vert P_j (P_{\leqq j-3}h \nabla ^2 h_{j,l})\Vert _{l^1_{l}L^2}\\&\lesssim \ \Vert h\Vert _{L^\infty }\Vert P_j h\Vert _{Y_j}\\&\lesssim \ \Vert |D|^{\sigma _d} h\Vert _{H^{s-\sigma _d}}\Vert P_j h\Vert _{Y_j}, \end{aligned} \end{aligned}$$

For the high-high interactions $P_j (\nabla P_l h \nabla P_l h)$, we have

$$\begin{aligned}&\Vert \sum _{l\geqq j}\Delta ^{-1}P_j (\nabla P_{l}h \nabla P_l h)\Vert _{Y_j}\\&\quad \lesssim \ 2^{-2j}\Big (\sum _{|j|\geqq l\geqq j}\Vert P_j (\nabla P_{l}h \nabla P_l h)\Vert _{l^1_{|j|}L^2} +\sum _{l> |j|}2^{l-|j|}\Vert P_j (\nabla P_{l}h \nabla P_l h)\Vert _{l^1_{l}L^2}\Big )\\&\quad \lesssim \ \sum _{|j|\geqq l\geqq j}2^{(d/2-2)j}\Vert \nabla P_{l}h \Vert _{L^2} \Vert \nabla P_l h\Vert _{L^2} +\sum _{l> |j|}2^{(d/2-2)j}2^{l-|j|}\Vert \nabla P_{l}h\Vert _{L^2}\Vert \nabla P_l h\Vert _{L^2}, \end{aligned}$$

From these two bounds, for $d\geqq 3$ we obtain

4.29 $$\begin{aligned} \Vert \Delta ^{-1}(h\nabla ^2 h+\nabla h\nabla h)\Vert _{Y^{s+2}_0}\lesssim \Vert |D|^{\sigma _d}h\Vert _{H^{s+2-\sigma _d}}\Vert h\Vert _{Y^{s+2}_0}+\Vert |D|^{\sigma _d}h\Vert _{H^{s+2-\sigma _d}}^2, \end{aligned}$$

and for $d=2$ we obtain

4.30 $$\begin{aligned} \Vert P_{\geqq 0}\Delta ^{-1}(h\nabla ^2 h+\nabla h\nabla h)\Vert _{Y^{s+2}_0}\lesssim \Vert |D|^{\sigma _d}h\Vert _{H^{s+2-\sigma _d}}\Vert h\Vert _{Y^{s+2}_0}+\Vert |D|^{\sigma _d}h\Vert _{H^{s+2-\sigma _d}}^2. \end{aligned}$$

We then bound the contribution of the $\lambda ^2$ source term. For the high-low or low-high interactions we have

$$\begin{aligned} \Vert \Delta ^{-1} P_j(P_{<j}\lambda P_j \lambda )\Vert _{Y_j}&\lesssim \ 2^{-2j}\Vert P_j ( P_{<j}\lambda P_j \lambda )\Vert _{l^1_{|j|}L^2}\\&\lesssim \ 2^{dj^-/2-2j} \Vert P_{<j}\lambda \Vert _{H^s}\Vert P_j \lambda \Vert _{L^2}, \end{aligned}$$

For the high-high interactions we have

$$\begin{aligned} \Vert \sum _{l\geqq j} \Delta ^{-1} P_j(P_{l}\lambda P_l \lambda )\Vert _{Y_j} \lesssim \ \sum _{|j|\geqq l\geqq j}2^{(d/2-2)j}\Vert P_{l}\lambda \Vert _{L^2}^2 +\sum _{l> |j|}2^{(d/2-2)j}2^{l-|j|}\Vert P_{l}\lambda \Vert _{L^2}^2. \end{aligned}$$

These two bounds also imply for $d\geqq 3$

4.31 $$\begin{aligned} \Vert \Delta ^{-1}(\lambda ^2)\Vert _{Y^{s+2}_0}\lesssim \Vert \lambda \Vert _{H^{s}}^2, \end{aligned}$$

and for $$d=2$$

4.32 $$\begin{aligned} \Vert P_{\geqq 0}\Delta ^{-1}(\lambda ^2)\Vert _{Y^{s+2}_0}\lesssim \Vert \lambda \Vert _{H^{s}}^2. \end{aligned}$$

Using (4.28), $\Vert h\Vert _{Y^{lo}_0}\leqq \epsilon _0$ and $\Vert \lambda \Vert _{H^s}\leqq \epsilon _0$, by *h*-equation, (4.29) and (4.31) we obtain for $d\geqq 3$

$$\begin{aligned} \Vert h\Vert _{Y^{s+2}_0}\lesssim \epsilon _0 \Vert h\Vert _{Y^{s+2}_0}+\epsilon _0^2. \end{aligned}$$

and by (4.30) and (4.32) we obtain for $d=2$

$$\begin{aligned} \Vert P_{\geqq 0}h\Vert _{Y^{s+2}_0}\lesssim \epsilon _0 \Vert h\Vert _{Y^{s+2}_0}+\epsilon _0^2\lesssim \epsilon _0 \Vert P_{\geqq 0}h\Vert _{Y^{s+2}_0}+\epsilon _0^2. \end{aligned}$$

This concludes the proof of the bounds (4.25) and (4.27). $\square$

*Step 3: Prove the bound* (4.8) *for* *A*. This is obtained by (4.24) and the following proposition. Here we solve the initial data $A_0$ from the elliptic div-curl system (2.19).

#### Proposition 4.7

(Initial data $A_0$) Let $d\geqq 2$, $s>d/2$ and $\delta _d=\delta$ if $d=2$ and $\delta _d=0$ if $d\geqq 3$. Assume that

4.33 $$\begin{aligned} \Vert \lambda \Vert _{H^s}\leqq \epsilon _0, \qquad \Vert |D|^{\sigma _d} h\Vert _{H^{s+1-\sigma _d}}\leqq \epsilon _0 \end{aligned}$$

Then the elliptic system (2.19) for *A* admits a unique small solution with

4.34 $$\begin{aligned} \Vert |D|^{\delta _d} A\Vert _{H^{s+1-\delta _d}}\lesssim \Vert \lambda \Vert _{H^s}. \end{aligned}$$

Moreover, assume that $p_{0k}$ is an admissible frequency envelope for $\lambda \in H^s$. Then we have the frequency envelope bounds

4.35 $$\begin{aligned} \Vert S_k |D|^{\delta _d} A\Vert _{H^{s+1-\delta _d}}\lesssim \epsilon _0 p_{0k}. \end{aligned}$$

In addition, for the linearization of the solution map above we also have the bound:

4.36 $$\begin{aligned} \Vert |D|^{\delta _d} {{\varvec{\delta }}} A\Vert _{H^{\sigma +1-\delta _d}}\lesssim \epsilon _0(\Vert |D|^{\sigma _d}{{\varvec{\delta }}} h\Vert _{H^{\sigma +1-\sigma _d}}+ \Vert {{\varvec{\delta }}} \lambda \Vert _{H^\sigma }),\qquad \sigma \in (d/2-2,s].\nonumber \\ \end{aligned}$$

#### Proof

Using the definition of covariant derivatives and the harmonic coordinate condition (2.23) we can rewrite the div-curl system (2.19) for *A* as

$$\begin{aligned} \partial ^\alpha A_\alpha = 0, \qquad \partial _\alpha A_{\beta }-\partial _{\beta }A_\alpha =\mathop {\textrm{Im}}\nolimits (\lambda _{\alpha \gamma }{\bar{\lambda }}^\gamma _{\beta }). \end{aligned}$$

Using these equations we derive a second order elliptic equation for *A*, namely

$$\begin{aligned} \begin{aligned} \partial _\gamma \partial ^\gamma A_\alpha&= \partial _\gamma g^{\gamma \beta } \partial _\alpha A_\beta - \partial _\gamma g^{\gamma \beta } (\partial _\alpha A_\beta -\partial _\beta A_\alpha ) \\&= (\partial _\gamma g^{\gamma \beta } \partial _\alpha - \partial _\alpha g^{\gamma \beta } \partial _\gamma ) A_\beta - \partial _\gamma \mathop {\textrm{Im}}\nolimits (\lambda _{\alpha \sigma }{\bar{\lambda }}^{\gamma \sigma }) \end{aligned} \end{aligned}$$

Here we have a leading order cancellation in the first term on the right, but we prefer to keep the divergence structure and rewrite this equation schematically in the form

$$\begin{aligned} \Delta A = \partial (\lambda ^2) + \partial ( h \partial A). \end{aligned}$$

This will be well suited in order to solve this equation via the contraction principle.

Precisely, we define the map $A\rightarrow {\mathcal {T}}(A)$ with ${\mathcal {T}}(A)$ satisfying

$$\begin{aligned} \Delta {\mathcal {T}}(A) = \partial (\lambda ^2) +\partial ( h\partial A). \end{aligned}$$

so that the solution *A* may be seen as a fixed point for ${\mathcal {T}}$. To use the contraction principle, it suffices to show that, under the assumption (4.33), this map is Lipschitz in the ball $\{ A:\Vert |D|^{\delta _d} A\Vert _{H^{s+1-\delta _d}}\leqq C\Vert \lambda \Vert _{H^s} \}$ with a small Lipschitz constant. This would yield the existence and uniqueness of solutions for *A*-equations and the bound (4.34).

To establish the contraction property, we consider the linearization of ${\mathcal {T}}$,

$$\begin{aligned} \Delta {\varvec{\delta }}{\mathcal {T}}(A)=\nabla (\lambda {{\varvec{\delta }}} \lambda )+\nabla ({{\varvec{\delta }}} h\nabla A+h\nabla {{\varvec{\delta }}} A), \end{aligned}$$

under the assumptions

$$\begin{aligned} \Vert |D|^{\delta _d} A\Vert _{H^{s+1-\delta _d}} + \Vert |D|^{\sigma _d}h\Vert _{H^{s+1-\sigma _d}}+ \Vert \lambda \Vert _{H^s}\lesssim \epsilon _0. \end{aligned}$$

Here we denote by ${{{\tilde{a}}}}_{0k}$, ${{{\tilde{c}}}}_{0k}$ and ${{{\tilde{p}}}}_{0k}$ are admissible frequency envelopes for $|D|^{\delta _d} {{\varvec{\delta }}} A\in H^{\sigma +1-\delta _d}$, $|D|^{\sigma _d}{{\varvec{\delta }}} h\in H^{\sigma +1-\sigma _d}$ and ${{\varvec{\delta }}} \lambda \in H^\sigma$ respectively. Under these assumptions we will prove that the above linearization satisfies the bound

4.37 $$\begin{aligned} \Vert S_k |D|^{\delta _d} {\varvec{\delta }}{\mathcal {T}}(A)\Vert _{H^{\sigma +1-\delta _d}}\lesssim \epsilon _0 ( {{{\tilde{a}}}}_{0k}+{{{\tilde{c}}}}_{0k}+{{{\tilde{p}}}}_{0k}), \end{aligned}$$

If the bound (4.37) is true, then by the contraction principle we immediately get a unique small solution *A* to our equations, as well as the linearized bound (4.36).

We can also use (4.37) in order to prove the frequency envelope bounds (4.35). Indeed, by (3.3) and (4.34) we have

$$\begin{aligned} a_{0k}&= 2^{-\delta k}\Vert |D|^{\delta _d} A\Vert _{H^{s+1-\delta _d}}+\max _{j}2^{-\delta |j-k|}\Vert S_j|D|^{\delta _d} A\Vert _{H^{s+1-\delta _d}}\\&\lesssim 2^{-\delta k} (\epsilon _0\Vert |D|^{\delta _d} A\Vert _{H^{s+1-\delta _d}}+\epsilon _0\Vert \lambda \Vert _{H^s})+\max _{j}2^{-\delta |j-k|}(\epsilon _0 a_{0j}+\epsilon _0 p_{0j})\\&\lesssim \epsilon _0 a_{0k}+\epsilon _0 p_{0k}. \end{aligned}$$

This implies (4.35) for $\epsilon _0$ sufficiently small.

It remains to prove the bound (4.37). We have

$$\begin{aligned} \Vert S_k |D|^{\delta _d} {\varvec{\delta }}{\mathcal {T}}(A) \Vert _{H^{\sigma +1-\delta _d}}\lesssim \Vert S_k |D|^{-1+\delta _d}({{\varvec{\delta }}} h\nabla A+h\nabla {{\varvec{\delta }}} A+\lambda {{\varvec{\delta }}} \lambda )\Vert _{H^{\sigma +1-\delta _d}}. \end{aligned}$$

Here we only estimate the term $\lambda {{\varvec{\delta }}} \lambda$; the others are similar. Precisely, when $k=0$, using a Littlewood–Paley decomposition, Bernstein’s inequality and (3.2) we obtain

$$\begin{aligned} \Vert |D|^{-1+\delta _d} S_0(\lambda {{\varvec{\delta }}} \lambda )\Vert _{L^2}&\lesssim \Vert \lambda \Vert _{L^2}\Vert S_0{{\varvec{\delta }}} \lambda \Vert _{L^2}+\sum _{j\geqq 0} 2^{-\sigma j}\Vert \lambda _j\Vert _{L^2} 2^{\sigma j}\Vert {{\varvec{\delta }}} \lambda _j\Vert _{L^2}\\&\lesssim \ \Vert \lambda \Vert _{L^2}{{{\tilde{p}}}}_{00}+ \sum _{j\geqq 0} 2^{(\delta -\sigma )j}\Vert \lambda _j\Vert _{L^2} {{{\tilde{p}}}}_{00}\\&\lesssim \ \Vert \lambda \Vert _{H^s}{{{\tilde{p}}}}_{00}. \end{aligned}$$

When $k>0$, we use Bernstein’s inequality to bound the high-low and low-high interactions by $\Vert \lambda \Vert _{H^s}{{{\tilde{p}}}}_{0k}$. For the high-high interaction we have

$$\begin{aligned}&\Vert |D|^{-1+\delta _d} S_k(\sum _{l>k}\lambda _l {{\varvec{\delta }}} \lambda _l)\Vert _{H^{\sigma +1-\delta _d}}\\&\quad \lesssim \ \sum _{l>k} 2^{(\sigma +\frac{d}{2})k}\Vert \lambda _l\Vert _{L^2}\Vert {{\varvec{\delta }}} \lambda _l\Vert _{L^2}\\&\quad \lesssim \ {{\textbf {1}}}_{\leqq -d/2}(\sigma )\sum _{l>k} 2^{(\sigma +\frac{d}{2}-\delta )k}2^{(\delta -\sigma )l}\Vert \lambda _l\Vert _{L^2}2^{\sigma l}\Vert {{\varvec{\delta }}} \lambda _l\Vert _{L^2}\\&\qquad +{{\textbf {1}}}_{> -d/2}(\sigma )\sum _{l>k} 2^{(\sigma +\frac{d}{2}+\delta )(k-l)}2^{(\frac{d}{2}+\delta )l}\Vert \lambda _l\Vert _{L^2}2^{\sigma l}\Vert {{\varvec{\delta }}} \lambda _l\Vert _{L^2}\\&\quad \lesssim \ \Vert \lambda \Vert _{H^s}{{{\tilde{p}}}}_{0k}. \end{aligned}$$

This concludes the proof of bound (4.37), and completes the proof of the lemma. $\square$

## Estimates for the Parabolic Equations

Here we consider the solvability of the parabolic system (2.29). For this purpose we view $\lambda \in l^2X^s$ as a parameter, and show that the solution $(h,A) \in {{\varvec{ {\mathcal {E}}}}}^s$ exists, it is small and has a Lipschitz dependence on both the initial data and on $\lambda$.

### Theorem 5.1

- Let $d \geqq 2$, $s > d/2$. Assume that $\Vert h_0\Vert _{{\textbf{Y}}_0^{s+2}}\leqq \epsilon$, $\Vert |D|^{\delta _d} A_0\Vert _{H^{s+1-\delta _d}}\leqq \epsilon$ and $\Vert \lambda \Vert _{l^2Z^s}\leqq \epsilon$. Then the parabolic system (2.29)–(2.30) admits a unique small solution ${{\mathcal {S}}}= (h,A)$ in ${{\varvec{ {\mathcal {E}}}}}^s$, with
  5.1 $$\begin{aligned} \Vert {{\mathcal {S}}}\Vert _{{{\varvec{ {\mathcal {E}}}}}^s} \lesssim \Vert {{\mathcal {S}}}_0\Vert _{{{\varvec{ {\mathcal {E}}}}}^s_0} + \Vert \lambda \Vert _{l^2Z^s}. \end{aligned}$$
  In addition this solution has a Lipschitz dependence on both ${{\mathcal {S}}}_0$ in ${{\varvec{ {\mathcal {E}}}}}^s_0$ and $\lambda$ in $l^2Z^s$. Moreover, assume that $s_{0k}$ and $p_k$ are admissible frequency envelopes for $(h_0, A_0)\in {{\varvec{ {\mathcal {E}}}}}_0^{s}$, $\lambda \in l^2Z^s$ respectively, we have the frequency envelope version
  5.2 $$\begin{aligned} \Vert {{\mathcal {S}}}_k \Vert _{{{\varvec{ {\mathcal {E}}}}}^s} \lesssim s_{0k} +\epsilon p_k. \end{aligned}$$
- In addition, for the linearization of the parabolic system (2.29) we have the bounds
  5.3 $$\begin{aligned} \Vert {{\varvec{\delta }}} {{\mathcal {S}}}\Vert _{{{\varvec{ {\mathcal {E}}}}}^s} \lesssim \Vert {{\varvec{\delta }}} {{\mathcal {S}}}_0\Vert _{{{\varvec{ {\mathcal {E}}}}}_0^{s}}+ \epsilon \Vert {{\varvec{\delta }}} \lambda \Vert _{l^2Z^s}, \end{aligned}$$
  and
  5.4 $$\begin{aligned} \Vert {{\varvec{\delta }}} {{\mathcal {S}}}\Vert _{{{\mathcal {E}}}^\sigma } \lesssim \Vert {{\varvec{\delta }}} {{\mathcal {S}}}_0\Vert _{{{\mathcal {H}}}^{\sigma }}+ \epsilon \Vert {{\varvec{\delta }}} \lambda \Vert _{Z^\sigma }, \end{aligned}$$
  for $$\sigma \in (\frac{d}{2}-2,s]$$.

We will do this in two steps. First we prove that this system is solvable in the larger space ${{\mathcal {E}}}^s$. Then we improve the space-time bounds for the metric *h* to the stronger norm ${\textbf{Y}}^{s+2}$; the latter will be needed in the study of the Schrödinger evolution (2.28).

### Lemma 5.2

Let $g=I_d+h$. Assume that $\Vert h\Vert _{ Z^{\sigma _d,s+1}}\leqq \epsilon$ for $s>d/2$ and $d\geqq 2$. Let $c_k$ and $a_k$ be admissible frequency envelopes for $h\in Z^{\sigma _d,s+1}$, respectively $A\in Z^{s}$. Then for any $d/2-2< \sigma \leqq s$ and a linearization operator ${\varvec{\delta }}$ we have

5.5 $$\begin{aligned} \Vert {\varvec{\delta }}(h g)\Vert _{Z^{\sigma _d,\sigma +1}}\lesssim & {} \Vert {{\varvec{\delta }}} h\Vert _{Z^{\sigma _d,\sigma +1}}, \end{aligned}$$

5.6 $$\begin{aligned} \Vert {\varvec{\delta }}(A g)\Vert _{Z^{\sigma }}\lesssim & {} \Vert {{\varvec{\delta }}} A\Vert _{Z^{\sigma }}+ \Vert A\Vert _{ Z^{s}} \Vert {{\varvec{\delta }}} h\Vert _{Z^{\sigma _d,\sigma }}, \end{aligned}$$

and hence we have

5.7 $$\begin{aligned} \Vert S_k(hg)\Vert _{Z^{\sigma _d, s+1}}&\lesssim c_k, \end{aligned}$$

5.8 $$\begin{aligned} \Vert S_k (A g)\Vert _{Z^{s}}&\lesssim a_k, \end{aligned}$$

### Proof

Assume that ${{{\tilde{c}}}}_k(\sigma )$ and ${{{\tilde{a}}}}_k$ are admissible frequency envelopes for ${{\varvec{\delta }}} h\in Z^{\sigma _d,\sigma }$ and ${{\varvec{\delta }}} A\in Z^{\sigma }$. Using a Littlewood–Paley decomposition, Bernstein’s inequality and the smallness of *h* we obtain

$$\begin{aligned} \Vert S_k({{\varvec{\delta }}} hh)\Vert _{ Z^{\sigma _d,\sigma +1}}\lesssim \epsilon {{{\tilde{c}}}}_k(\sigma +1)+\Vert {{\varvec{\delta }}} h\Vert _{ Z^{\sigma _d,\sigma +1}}c_k. \end{aligned}$$

This implies (5.5) and (5.7) immediately. For *A* we have

$$\begin{aligned} \Vert S_k({{\varvec{\delta }}} Ah)\Vert _{ Z^{\sigma }}\lesssim \epsilon {{{\tilde{a}}}}_k, \end{aligned}$$

and

$$\begin{aligned} \Vert S_k(A {{\varvec{\delta }}} h)\Vert _{Z^{\sigma }}\lesssim a_k \Vert {{\varvec{\delta }}} h\Vert _{ Z^{\sigma _d,\sigma }} +\Vert A\Vert _{ Z^{s}} {{{\tilde{c}}}}_k(\sigma ). \end{aligned}$$

These give (5.6) and (5.8). $\square$

Now, we begin to solve the parabolic system (2.29) with initial data (2.30) as follows:

### Proposition 5.3

- Assume that $\Vert (h_0,A_0)\Vert _{{{\mathcal {H}}}^s}\leqq \epsilon$ and $\Vert \lambda \Vert _{Z^s}\leqq \epsilon$ for $s > d/2$ and $d \geqq 2$. Then the parabolic system (2.29)–(2.30) admits a unique small solution ${{\mathcal {S}}}= (h,A)$ in ${\mathcal {E}}^s$, with
  5.9 $$\begin{aligned} \Vert {{\mathcal {S}}}\Vert _{{\mathcal {E}}^s} \lesssim \Vert {{\mathcal {S}}}_0\Vert _{{{\mathcal {H}}}^{s}}+ \Vert \lambda \Vert _{Z^s}. \end{aligned}$$
  In addition this solution has a Lipschitz dependence on ${{\mathcal {S}}}_0$ in ${{\mathcal {H}}}^{s}$ and $\lambda$ in $Z^s$. Moreover, assume that $s_{0k}$ and $p_k$ are admissible frequency envelopes for ${{\mathcal {S}}}_0\in {{\mathcal {H}}}^{s}$, $\lambda \in Z^s$ respectively, then we have the frequency envelope version
  5.10 $$\begin{aligned} \Vert {{\mathcal {S}}}_k \Vert _{{\mathcal {E}}^s} \lesssim s_{0k} +\epsilon p_k. \end{aligned}$$
- In addition, for the linearization of the parabolic system (2.29) we have the bounds
  5.11 $$\begin{aligned} \Vert {{\varvec{\delta }}} {{\mathcal {S}}}\Vert _{{\mathcal {E}}^\sigma } \lesssim \Vert {{\varvec{\delta }}} {{\mathcal {S}}}(0)\Vert _{{{\mathcal {H}}}^{\sigma }}+ \epsilon \Vert {{\varvec{\delta }}} \lambda \Vert _{Z^\sigma }, \end{aligned}$$
  for $$\sigma \in (\frac{d}{2}-2,s]$$.

### Proof

First, we consider a linear equation and prove a linear estimate. Precisely, assume that the frequency localized function $u_k$ is solution of the linear equation

$$\begin{aligned} \partial _t u_k-\Delta u_k=f_k,\qquad u_k(0)=u_{0k}. \end{aligned}$$

Then by Bernstein’s inequality we have the linear estimates

$$\begin{aligned} \frac{1}{2}\frac{\textrm{d}}{\textrm{d}t}\Vert u_k\Vert _{L^2}^2 \leqq&- c 2^{2k} \Vert u_k\Vert _{L^2}^2+\Vert u_k\Vert _{L^2}\Vert f_k\Vert _{L^2}. \end{aligned}$$

We cancel one $\Vert u_k\Vert _{L^2}$, then multiply both sides by $e^{c 2^{2k}t}$ and integrate in time to obtain

5.12 $$\begin{aligned} \begin{aligned} \Vert u_k(t)\Vert _{L^2}&\lesssim e^{-c 2^{2k}t}\Vert u_{0k}\Vert _{L^2}+2^{-2k} \Vert f_k(s)\Vert _{L^\infty L^2}. \end{aligned} \end{aligned}$$

In order to solve (2.29) with small initial data, it suffices to consider the following linearized equations

$$\begin{aligned}&\partial _t {{\varvec{\delta }}} h_k -\Delta {{\varvec{\delta }}} h_k={\mathcal {N}}_1,\qquad \partial _t {{\varvec{\delta }}} A_k -\Delta {{\varvec{\delta }}} A_k={\mathcal {N}}_2, \end{aligned}$$

where the nonlinearities ${\mathcal {N}}_1$ and ${\mathcal {N}}_2$ are

$$\begin{aligned} {\mathcal {N}}_1&=S_k({{\varvec{\delta }}} h\nabla ^2 h+h\nabla ^2{{\varvec{\delta }}} h+{{\varvec{\delta }}} h\nabla h\nabla h+g\nabla h \nabla {{\varvec{\delta }}} h+\lambda {{\varvec{\delta }}} \lambda ),\\ {\mathcal {N}}_2&=S_k(h\nabla ^2 {{\varvec{\delta }}} A+{{\varvec{\delta }}} h\nabla ^2 A+\nabla h\nabla {{\varvec{\delta }}} A+\nabla {{\varvec{\delta }}} h\nabla A\\&\quad +\nabla ^2 h\ {{\varvec{\delta }}} A+\nabla ^2{{\varvec{\delta }}} h\ A +\nabla h\nabla h {{\varvec{\delta }}} A+\nabla h\nabla {{\varvec{\delta }}} hA +\lambda \nabla {{\varvec{\delta }}} \lambda \\&\quad +{{\varvec{\delta }}} \lambda \nabla \lambda +\lambda {{\varvec{\delta }}} \lambda (\nabla h+A)+\lambda ^2(\nabla {{\varvec{\delta }}} h+{{\varvec{\delta }}} A)), \end{aligned}$$

with *h*, *A* and $\lambda$ satisfying $\Vert (h,A)\Vert _{{{\mathcal {E}}}^s}\lesssim \epsilon$, $\Vert \lambda \Vert _{Z^s}\leqq \epsilon$. Then we will prove the bound

5.13 $$\begin{aligned} \begin{aligned} \Vert S_k{{\varvec{\delta }}} {{\mathcal {S}}}\Vert _{{{\mathcal {E}}}^{\sigma }} \lesssim {{{\tilde{s}}}}_{0k}+\epsilon ({{{\tilde{s}}}}_{k}+{{{\tilde{p}}}}_k)+(s_k+p_k)(\Vert {{\varvec{\delta }}} {{\mathcal {S}}}\Vert _{{{\mathcal {E}}}^{\sigma }}+\Vert {{\varvec{\delta }}} \lambda \Vert _{Z^\sigma }), \end{aligned} \end{aligned}$$

where ${{{\tilde{s}}}}_{0k}$, ${{{\tilde{s}}}}_k$, ${{{\tilde{p}}}}_k$ and $s_k$ are admissible frequency envelopes for ${{\varvec{\delta }}} {{\mathcal {S}}}(0)\in {{\mathcal {H}}}^\sigma$, ${{\varvec{\delta }}} {{\mathcal {S}}}\in {{\mathcal {E}}}^\sigma$, ${{\varvec{\delta }}} \lambda \in Z^\sigma$ and ${{\mathcal {S}}}\in {{\mathcal {E}}}^s$ respectively.

Assuming the bound (5.13) is true, then we can use the contraction mapping principle to solve the parabolic system (2.29) in the space

$$\begin{aligned} \big \{ {{\mathcal {S}}}=(h,A)\in {{\mathcal {E}}}^s:\Vert {{\mathcal {S}}}\Vert _{{{\mathcal {E}}}^{s}}\leqq C(\Vert {{\mathcal {S}}}_0\Vert _{{{\mathcal {H}}}^{s}}+ \Vert \lambda \Vert _{Z^s})\leqq 2C\epsilon \big \}, \end{aligned}$$

which also implies the bound (5.9).

By the definition of frequency envelopes (3.3) and (5.9), the bound (5.13) with $\sigma =s$ and ${{\varvec{\delta }}}=Id$ implies

$$\begin{aligned} s_k \lesssim s_{0k}+\epsilon (s_k+p_k). \end{aligned}$$

Thus the bound (5.10) follows. By (5.9), the bound (5.13) also gives (5.11).

We now return to the proof of (5.13). By the energy estimates in (5.12) we have

$$\begin{aligned} \Vert S_k{{\varvec{\delta }}} {{\mathcal {S}}}(t)\Vert _{{{\mathcal {E}}}^{\sigma }} \lesssim \Vert S_k{{\varvec{\delta }}} {{\mathcal {S}}}_0\Vert _{{{\mathcal {H}}}^{\sigma }} +\Vert |D|^{\sigma _d} S_k{\mathcal {N}}_1\Vert _{L^\infty H^{\sigma -\sigma _d}} +\Vert S_k{\mathcal {N}}_2\Vert _{L^\infty H^{\sigma -1}}. \end{aligned}$$

The estimates for the nonlinearities are similar, here we only estimate the following terms.

*A. The estimate for the terms* $h\nabla ^2{{\varvec{\delta }}} h$ *and* $\lambda {{\varvec{\delta }}} \lambda$ *in* ${\mathcal {N}}_1$. Using a Littlewood–Paley decomposition we have

$$\begin{aligned} \Vert S_k(h\nabla ^2{{\varvec{\delta }}} h)\Vert _{L^\infty H^\sigma }&\lesssim \ 2^{\sigma k} \Vert h_{\leqq k}\Vert _{L^\infty L^\infty }\Vert \nabla ^2{{\varvec{\delta }}} h_k\Vert _{L^\infty L^2}\\&\quad +\sum _{l\leqq k} 2^{\sigma k+(d/2+2)l}\Vert h_{k}\Vert _{L^\infty L^2}\Vert {{\varvec{\delta }}} h_l\Vert _{L^\infty L^2}\\&\quad +\sum _{l> k} 2^{(\sigma +d/2) (k-l)+(\sigma +d/2+2)l}\Vert h_{l}\Vert _{L^\infty L^2}\Vert {{\varvec{\delta }}} h_l\Vert _{L^\infty L^2}\\&\lesssim \ \Vert |D|^{\sigma _d} h\Vert _{L^\infty H^{s-\sigma _d}}{{{\tilde{c}}}}_k+2^{sk+2\delta k} \Vert h_k\Vert _{L^\infty L^2}{{{\tilde{c}}}}_k\\&\quad +{{{\tilde{c}}}}_k \Vert |D|^{\sigma _d}h\Vert _{L^\infty H^{s+1-\sigma _d}}\\&\lesssim \ {{{\tilde{c}}}}_k \Vert |D|^{\sigma _d}h\Vert _{L^\infty H^{s+1-\sigma _d}}, \end{aligned}$$

and

$$\begin{aligned} \Vert S_k(\lambda {{\varvec{\delta }}} \lambda )\Vert _{L^\infty H^\sigma }&\lesssim \ 2^{\sigma k} \Vert \lambda _{\leqq k}\Vert _{L^\infty L^\infty }\Vert {{\varvec{\delta }}} \lambda _k\Vert _{L^\infty L^2}+\sum _{l\leqq k} 2^{\sigma k+dl/2}\Vert \lambda _{k}\Vert _{L^\infty L^2}\Vert {{\varvec{\delta }}} \lambda _l\Vert _{L^\infty L^2}\\&\quad +\sum _{l> k} 2^{(\sigma +d/2) (k-l)+(\sigma +d/2)l}\Vert \lambda _{l}\Vert _{L^\infty L^2}\Vert {{\varvec{\delta }}} \lambda _l\Vert _{L^\infty L^2}\\&\lesssim \ \Vert \lambda \Vert _{L^\infty H^s}{{{\tilde{p}}}}_k+p_k \Vert {{\varvec{\delta }}} \lambda \Vert _{L^\infty H^\sigma }+\sum _{l> k} 2^{(\sigma +d/2-\delta ) (k-l)}\Vert \lambda _{l}\Vert _{L^\infty H^{d/2}}{{{\tilde{p}}}}_k\\&\lesssim \ {{{\tilde{p}}}}_k\Vert \lambda \Vert _{L^\infty H^s}+p_k \Vert {{\varvec{\delta }}} \lambda \Vert _{L^\infty H^\sigma }. \end{aligned}$$

*B. The estimate for the terms* $\nabla ^2 h{{\varvec{\delta }}} A$ *and* $\lambda \nabla {{\varvec{\delta }}} \lambda$ *in* ${\mathcal {N}}_2$. The second term is estimated in the same manner as the above bound for $\lambda {{\varvec{\delta }}} \lambda$ in ${\mathcal {N}}_1$. For the first term $\nabla ^2 h{{\varvec{\delta }}} A$ we have

$$\begin{aligned}&\Vert S_k(\nabla ^2 h{{\varvec{\delta }}} A)\Vert _{L^\infty H^{\sigma -1}}\\&\quad \lesssim \ \Vert |D|^{\sigma _d} h\Vert _{L^\infty H^{s+1-\sigma _d}}\Vert {{\varvec{\delta }}} A_k\Vert _{L^\infty H^{\sigma }}+\sum _{l\leqq k} 2^{\sigma k+k}\Vert h_{k}\Vert _{L^\infty L^2}\Vert {{\varvec{\delta }}} A_l\Vert _{L^\infty H^{d/2}}\\&\qquad +\sum _{l> k} 2^{(\sigma +d/2-1) (k-l)+(\sigma +d/2+1)l}\Vert h_{l}\Vert _{L^\infty L^2}\Vert {{\varvec{\delta }}} A_l\Vert _{L^\infty L^2}\\&\quad \lesssim \ \epsilon {{{\tilde{a}}}}_k+c_k\Vert {{\varvec{\delta }}} A\Vert _{L^\infty H^{\sigma +1}}+\sum _{l> k} 2^{(\sigma +d/2-1) (k-l)}\Vert h_{l}\Vert _{L^\infty H^{d/2+\delta }}{{{\tilde{a}}}}_k\\&\quad \lesssim \ \epsilon {{{\tilde{a}}}}_k+c_k\Vert {{\varvec{\delta }}} A\Vert _{L^\infty H^{\sigma +1}}. \end{aligned}$$

This concludes the proof of the bound (5.13), and completes the proof of the theorem. $\square$

We continue with the bound for the $l^2Z^{\sigma _d,s+2}$-norm of the metric *h*.

### Proposition 5.4

Assume that $\Vert (h_0,A_0)\Vert _{{{\mathcal {H}}}^s}\leqq \epsilon$ and $\Vert \lambda \Vert _{l^2Z^s}\leqq \epsilon$ for $s>d/2$ and $d\geqq 2$. Then the solution *h* also belongs to $l^2Z^{s+2}$ and satisfies the bounds

5.14 $$\begin{aligned} \Vert h\Vert _{l^2Z^{\sigma _d,s+2}} \lesssim \Vert |D|^{\sigma _d}h_0\Vert _{H^{s+2-\sigma _d}}+ \Vert \lambda \Vert _{l^2 Z^s}. \end{aligned}$$

Assume that $c_{0k}$ and $p_k$ are admissible frequency envelopes for $|D|^{\sigma _d}h_0\in H^{s+2-\sigma _d}$, $\lambda \in l^2Z^s$ respectively. Then we have the frequency envelope bounds

5.15 $$\begin{aligned} \Vert S_k h\Vert _{l^2 Z^{\sigma _d,s+2}} \lesssim c_{0k}+\epsilon p_k. \end{aligned}$$

Finally, for the linearization of the *h*-equations we have the bounds

5.16 $$\begin{aligned} \Vert {{\varvec{\delta }}} h\Vert _{l^2Z^{\sigma _d,s+2}} \lesssim \Vert |D|^{\sigma _d}{{\varvec{\delta }}} h_0\Vert _{H^{s+2-\sigma _d}}+\epsilon \Vert {{\varvec{\delta }}} \lambda \Vert _{l^2Z^{s}}. \end{aligned}$$

### Proof of Proposition 5.4

We split the proof into two steps, where we first prove the appropriate bound for the linear constant coefficient heat flow and then we apply that bound to solve the nonlinear problem perturbatively.

Step 1. Here we consider the linear equations

5.17 $$\begin{aligned} \partial _t P_k u-\Delta P_k u=P_k f, \end{aligned}$$

with $P_k u$ localized at frequency $2^k$ for $k\in {\mathbb {Z}}$, and prove that

5.18 $$\begin{aligned} \Vert P_k u\Vert _{l_{|k|}^2L^\infty L^2}&\lesssim \Vert P_k u(0)\Vert _{L^2}+2^{-2k^+} \Vert P_k f\Vert _{l_{|k|}^2L^\infty L^2}. \end{aligned}$$

By Duhamel’s formula, we have

$$\begin{aligned} \Vert P_k u\Vert _{l^2_{|k|}L^\infty L^2}\lesssim \Vert e^{t\Delta } P_k u_0\Vert _{l^2_{|k|}L^\infty L^2}+\Vert \int _0^t e^{(t-s)\Delta } P_k f \textrm{d}s\Vert _{l^2_{|k|}L^\infty L^2}. \end{aligned}$$

Then we use (3.5) and (3.6) to bound the above two terms respectively, then we obtain (5.18).

*Step 2.* Here it suffices to write the linearized *h* equation in the form

$$\begin{aligned} \partial _t {{\varvec{\delta }}} h-\Delta {{\varvec{\delta }}} h={{\varvec{\delta }}} h\nabla ^2 h+h\nabla ^2{{\varvec{\delta }}} h+{{\varvec{\delta }}} h\nabla h\nabla h+g\nabla h \nabla {{\varvec{\delta }}} h+\lambda {{\varvec{\delta }}} \lambda :={\mathcal {N}}, \end{aligned}$$

and to prove that

5.19 $$\begin{aligned} \begin{aligned} \Vert S_k{{\varvec{\delta }}} h\Vert _{l^2 Z^{\sigma _d,s+2}}&\lesssim \Vert |D|^{\sigma _d}S_k{{\varvec{\delta }}} h_0\Vert _{H^{s+2-\sigma _d}}+\epsilon ({{{\tilde{c}}}}_k+{{{\tilde{p}}}}_k) \\&\quad + (c_k+p_k)( \Vert {{\varvec{\delta }}} h\Vert _{l^2Z^{\sigma _d,s+2}}+\Vert {{\varvec{\delta }}} \lambda \Vert _{l^2Z^{s}}), \end{aligned} \end{aligned}$$

where ${{{\tilde{c}}}}_k,\ {{{\tilde{p}}}}_k$ and $c_k$ are admissible frequency envelopes for ${{\varvec{\delta }}} h\in l^2 Z^{\sigma _d,s+2}$, ${{\varvec{\delta }}} \lambda \in l^2Z^{s}$ and $h\in l^2 Z^{\sigma _d,s+2}$ respectively.

If the bound (5.19) is true, then we choose the operator ${{\varvec{\delta }}}=Id$ to obtain (5.14). Then by (5.19) and (3.3) we also obtain (5.15). The bound (5.19) combined with (5.14) also implies (5.16).

We now continue with the proof of (5.19). By (5.18) we have

$$\begin{aligned} \Vert S_k {{\varvec{\delta }}} h\Vert _{l^2Z^{\sigma _d,s+2}}&\lesssim \Vert |D|^{\sigma _d}{{\varvec{\delta }}} h_{0k}\Vert _{H^{s+2-\sigma _d}}+\Vert S_k {\mathcal {N}}\Vert _{l^2Z^{\sigma _d,s}}. \end{aligned}$$

For the nonlinearities, we only estimate $h\nabla ^2 {{\varvec{\delta }}} h$ and $\lambda {{\varvec{\delta }}} \lambda$, the others are estimated similiarly. Indeed, using a Littlewood–Paley decomposition we have

$$\begin{aligned} \Vert P_k(h\nabla ^2{{\varvec{\delta }}} h)\Vert _{l^2_{|k|}L^\infty L^2}&\lesssim \ \Vert h\Vert _{L^\infty L^\infty }2^{2k}\Vert P_k{{\varvec{\delta }}} h\Vert _{l^2_{|k|}L^\infty L^2} +\Vert P_k h\Vert _{l^2_{|k|}L^\infty L^2}\Vert \nabla ^2{{\varvec{\delta }}} h\Vert _{L^\infty L^\infty }\\&\quad +\sum _{|k|\geqq l>k} 2^{\frac{d}{2}k+2l} \Vert P_l h\Vert _{L^\infty L^2}\Vert P_l{{\varvec{\delta }}} h\Vert _{l^2_lL^\infty L^2}\\&\quad +\sum _{l>|k|} 2^{(\frac{d}{2}+2)l} \Vert P_l h\Vert _{L^\infty L^2}\Vert P_l{{\varvec{\delta }}} h\Vert _{l^2_lL^\infty L^2}. \end{aligned}$$

By this estimate and Sobolev embeddings we obtain

$$\begin{aligned} \Vert S_k(h\nabla ^2{{\varvec{\delta }}} h)\Vert _{l^2 Z^{\sigma _d,s}}&\lesssim \ \Vert |D|^{\sigma _d}h\Vert _{L^\infty H^{s-\sigma _d}} {{{\tilde{c}}}}_k+c_k \Vert |D|^{\sigma _d}h\Vert _{L^\infty H^{s+2-\sigma _d}}. \end{aligned}$$

For the term $\lambda {{\varvec{\delta }}} \lambda$, we also have

$$\begin{aligned} 2^{s k}\Vert S_k(\lambda {{\varvec{\delta }}} \lambda )\Vert _{l^2_kL^\infty L^2}&\lesssim \ \Vert \lambda \Vert _{l^2Z^s}\Vert {{\varvec{\delta }}} \lambda _k\Vert _{L^\infty H^{s}}+ \Vert \lambda _k\Vert _{l^2_kL^\infty H^s}\Vert {{\varvec{\delta }}} \lambda \Vert _{Z^{s}}\\&\quad +\sum _{l>k} 2^{s k} 2^{\frac{d}{2}l} \Vert \lambda _l\Vert _{L^\infty L^2}\Vert {{\varvec{\delta }}} \lambda _l\Vert _{l^2_lL^\infty L^2}\\&\lesssim \ \epsilon {{{\tilde{p}}}}_k + p_k \Vert {{\varvec{\delta }}} \lambda \Vert _{l^2Z^{s}}. \end{aligned}$$

This completes the proof of Proposition 5.4. $\square$

Finally, we carry out the last step in the proof of Theorem 5.1, and establish bounds for the solutions *h* in the $Y^{s+2}$ spaces:

### Proposition 5.5

Let $d\geqq 2$, $s>d/2$. Assume that $\Vert (h_0,A_0)\Vert _{{{\mathcal {H}}}^s}\leqq \epsilon$ and $\Vert \lambda \Vert _{l^2Z^s}\leqq \epsilon$. Then we have the bound

5.20 $$\begin{aligned} \Vert h\Vert _{Y^{s+2}} \lesssim \Vert h_0\Vert _{{\textbf{Y}}_0^{s+2}}+\Vert \lambda \Vert _{l^2Z^s}. \end{aligned}$$

with Lipschitz dependence on the initial data in these topologies. Moreover, assume that $c_{0k}$ and $p_k$ are admissible frequency envelope for $h(0)\in {\textbf{Y}}_0^{s+2}$ and $\lambda \in l^2 Z^{s}$, then we have the frequency envelope version

5.21 $$\begin{aligned} \Vert S_k h \Vert _{Y^{s+2}} \lesssim c_{0k}+ \epsilon p_k . \end{aligned}$$

In addition, for the linearization of the elliptic system (2.29) we have the bounds

5.22 $$\begin{aligned} \Vert {{\varvec{\delta }}} h\Vert _{Y^{s+2}} \lesssim \Vert {{\varvec{\delta }}} h_0 \Vert _{{\textbf{Y}}_0^{s+2}} +\epsilon \Vert {{\varvec{\delta }}} \lambda \Vert _{l^2Z^{s}}. \end{aligned}$$

### Proof

Again it suffices to write the *h* equation in the form:

$$\begin{aligned} \partial _t {{\varvec{\delta }}} h-\Delta {{\varvec{\delta }}} h={{\varvec{\delta }}} h\nabla ^2 h+h\nabla ^2{{\varvec{\delta }}} h+{{\varvec{\delta }}} h\nabla h\nabla h+g\nabla h \nabla {{\varvec{\delta }}} h+\lambda {{\varvec{\delta }}} \lambda :={\mathcal {N}}, \end{aligned}$$

and to prove that

5.23 $$\begin{aligned} \Vert {{\varvec{\delta }}} h_k\Vert _{Y^{s+2}}&\lesssim \Vert {{\varvec{\delta }}} h_{0k}\Vert _{Y_0^{s+2}} +\epsilon ({{{\tilde{c}}}}_k+{{{\tilde{p}}}}_k)+(c_k+p_k)(\Vert {{\varvec{\delta }}} h\Vert _{{\textbf{Y}}^{s+2}}+\Vert {{\varvec{\delta }}} \lambda \Vert _{l^2Z^s}) ,\nonumber \\ \end{aligned}$$

where ${{{\tilde{c}}}}_k$ and ${{{\tilde{p}}}}_k$ are admissible frequency envelopes for ${{\varvec{\delta }}} h\in {\textbf{Y}}^{s+2}$ and ${{\varvec{\delta }}} \lambda \in l^2Z^{s}$ respectively.

If (5.23) is true, then the bound (5.20) is obtained by (5.23) with the operator ${{\varvec{\delta }}}=Id$ and the bound (5.14). We also obtain (5.21) by (5.23) and (5.15). The bound (5.23) combined with (5.16) also implies (5.22).

We now return to prove the bound (5.23). By Duhamel’s formula, (3.5) and (3.6), we have

$$\begin{aligned} \Vert S_k{{\varvec{\delta }}} h\Vert _{Y^{s+2}}\lesssim \Vert e^{t\Delta }{{\varvec{\delta }}} h_{0k}\Vert _{Y^{ s+2}}+\Vert \int _0^t e^{(t-s)\Delta } S_k{\mathcal {N}}\ \textrm{d}s\Vert _{Y^{ s+2}}. \end{aligned}$$

We estimate the first term in the right hand side. For any decomposition $P_j {{\varvec{\delta }}} h(0)=\sum _{l\geqq |j|} {{\varvec{\delta }}} h_{j,l}(0)$, by (3.5) we have

$$\begin{aligned} \Vert e^{t\Delta } P_j {{\varvec{\delta }}} h(0)\Vert _{Y_j}&\lesssim \inf _{P_j {{\varvec{\delta }}} h(0)=\sum _{l\geqq |j|} h_{j,l}(0)}\sum _{l\geqq |j|} 2^{l-|j|}\Vert e^{t\Delta } {{\varvec{\delta }}} h_{j,l}(0)\Vert _{l^1_{|l|}L^\infty L^2}\\&\lesssim \inf _{P_j {{\varvec{\delta }}} h(0)=\sum _{l\geqq |j|} {{\varvec{\delta }}} h_{j,l}(0)}\sum _{l\geqq |j|} 2^{l-|j|}\Vert {{\varvec{\delta }}} h_{j,l}(0)\Vert _{l^1_{|l|} L^2}\\&= \Vert {{\varvec{\delta }}} h(0)\Vert _{Y_{0j}}. \end{aligned}$$

This gives the bound for the first term.

Next, for the nonlinearities, we only estimate the Duhamel contributions of $h\nabla ^2{{\varvec{\delta }}} h$ and $\lambda {{\varvec{\delta }}} \lambda$ in detail. In order to bound the contribution of term $h\nabla ^2{{\varvec{\delta }}} h$, we use the Littlewood–Paley trichotomy to decompose it into three cases:

*a) Low-high interactions:* $P_j(P_{<j}h\nabla ^2 P_{j}{{\varvec{\delta }}} h)$. By (3.6), for any decomposition $P_j {{\varvec{\delta }}} h=\sum _{l\geqq |j|} {{\varvec{\delta }}} h_{j,l}$ we have

$$\begin{aligned} \Vert \int _0^t e^{(t-s)\Delta }P_j(P_{<j}h\nabla ^2 P_j{{\varvec{\delta }}} h) \textrm{d}s\Vert _{Y_j}&=\ \sum _{l\geqq |j|} 2^{l-|j|} 2^{-2j^+}\Vert P_j(P_{<j}h\nabla ^2 {{\varvec{\delta }}} h_{j,l}) \Vert _{l^1_{l}L^\infty L^2}\\&\lesssim \ 2^{2j-2j^+} \Vert P_{<j}h\Vert _{L^\infty L^\infty }\sum _{l\geqq |j|} 2^{l-|j|}\Vert {{\varvec{\delta }}} h_{j,l} \Vert _{l^1_l L^\infty L^2}\\&\lesssim \ 2^{2j-2j^+}\Vert |D|^{\sigma _d}h\Vert _{L^\infty H^{s-\sigma _d}}\Vert P_j{{\varvec{\delta }}} h\Vert _{Y_j}. \end{aligned}$$

This implies both the low-frequency part bound

$$\begin{aligned} \Vert \int _0^t e^{(t-s)\Delta } \sum _{j\leqq 0} P_j(P_{<j}h\nabla ^2 P_j{{\varvec{\delta }}} h) \textrm{d}s\Vert _{Y^{ s+2}}\lesssim \epsilon \Vert S_0{{\varvec{\delta }}} h\Vert _{Y^{ s+2}}, \end{aligned}$$

and the high frequency part bound

$$\begin{aligned} \Vert \int _0^t e^{(t-s)\Delta }S_j(P_{<j}h\nabla ^2 P_j{{\varvec{\delta }}} h) \textrm{d}s\Vert _{Y^{ s+2}}\lesssim \epsilon \Vert {{\varvec{\delta }}} h_j\Vert _{Y^{ s+2}}. \end{aligned}$$

*b) The high-low interactions* $P_j(P_j h\nabla ^2 P_{<j+O(1)}{{\varvec{\delta }}} h)$ are estimated in the same manner as the above *low-high* case, so we omit the computations.

*c) High-high interactions:* $\sum _{l>j}P_j(P_lh\nabla ^2 P_l {{\varvec{\delta }}} h)$. This sum can be further decomposed as $\sum _{l>j}=\sum _{|j|>l>j}+\sum _{l\geqq |j|}$. Then by (3.6) we bound the contribution of the first term by

$$\begin{aligned}&2^{(\frac{d}{2}-\delta )j^-}\Vert \int _0^t e^{(t-s)\Delta }\sum _{|j|>l>j}P_j(P_lh \nabla ^2 P_l {{\varvec{\delta }}} h) \textrm{d}s\Vert _{Y_j}\\&\quad \lesssim 2^{(\frac{d}{2}-\delta )j^-} \sum _{|j|>l>j} \Vert P_j(P_lh\nabla ^2 P_l {{\varvec{\delta }}} h) \Vert _{l^1_{|j|}L^\infty L^2}\\&\quad \lesssim \ \sum _{|j|>l>j} 2^{(d-\delta )(j-l)+(d+2-\delta )l} \Vert P_lh\Vert _{l^2_{|j|}L^\infty L^2}\Vert P_l{{\varvec{\delta }}} h\Vert _{l^2_{|j|}L^\infty L^2}\\&\quad \lesssim \ \sum _{|j|>l>j} 2^{(d-\delta )(j-l)} \Vert P_lh\Vert _{l^2 Z^{\sigma _d,s}}{{{\tilde{c}}}}_0. \end{aligned}$$

Also by (3.6) we bound the contribution of second term by

5.24 $$\begin{aligned}&2^{(\frac{d}{2}-\delta )j^-+( s+2)j^+}\Vert \int _0^t e^{(t-s)\Delta }\sum _{l>|j|}P_j(h_{l}\nabla ^2 {{\varvec{\delta }}} h_l) \textrm{d}s\Vert _{Y_j}\nonumber \\&\quad \lesssim \ 2^{(\frac{d}{2}-\delta )j^-+( s+2)j^+} \sum _{l>|j|}2^{l-|j|}\Vert \int _0^t e^{(t-s)\Delta } P_j(h_{l}\nabla ^2 {{\varvec{\delta }}} h_{l}) \textrm{d}s\Vert _{l^1_{l}L^\infty L^2}\nonumber \\&\quad \lesssim \ 2^{(\frac{d}{2}-\delta )j^-+( s+2)j^+}\sum _{l>|j|}2^{l-|j|+dj/2-2j^+ +2l} \Vert h_{l} \Vert _{l^2_{|j|}L^\infty L^2}\Vert {{\varvec{\delta }}} h_{l} \Vert _{l^2_{|j|}L^\infty L^2} \end{aligned}$$

This term is further controlled by

$$\begin{aligned} \text {LHS}(\text {5.24})&\lesssim \ 2^{(d+1-\delta )j^-+( s+\frac{d}{2}-1)j^+} \sum _{l>|j|}2^{3l}\Vert h_{l}\Vert _{l^2_{l}L^\infty L^2}\Vert {{\varvec{\delta }}} h_{l}\Vert _{l^2_{l}L^\infty L^2}\\&\lesssim \ 2^{(d+1-\delta )j^-} \Vert h \Vert _{l^2 Z^{\sigma _d,s+1}} {{{\tilde{c}}}}_0+ {\textbf{1}}_{>0}(j)\Vert h\Vert _{l^2Z^{\sigma _d,s+1}} {{{\tilde{c}}}}_j. \end{aligned}$$

This concludes the proof of the bound for the contribution of $h\nabla ^2{{\varvec{\delta }}} h$. Next we consider the term $\lambda {{\varvec{\delta }}} \lambda$. We also split its analysis into three cases:

*a) Low-high interactions:* $P_j(P_{<j}\lambda P_j{{\varvec{\delta }}} \lambda )$ *and high-low interactions:* $P_j(P_j\lambda P_{<j}{{\varvec{\delta }}} \lambda )$. These two cases are similar, we only estimate the first term. By (3.6), we have

$$\begin{aligned} \Vert \int _0^t e^{(t-s)\Delta }P_j(P_{<j}\lambda P_j{{\varvec{\delta }}} \lambda ) \textrm{d}s\Vert _{Y_j}&\lesssim \ 2^{-2j^+} \Vert P_j(P_{<j}\lambda P_j{{\varvec{\delta }}} \lambda ) \Vert _{l^1_{|j|}L^\infty L^2}\\&\lesssim \ 2^{-2j^+}\Vert \lambda \Vert _{l^2Z^s} \Vert P_j{{\varvec{\delta }}} \lambda \Vert _{l^2_{|j|}L^\infty L^2}, \end{aligned}$$

which is acceptable.

*b) High-high interactions:* $\sum _{l>j}P_j(P_l\lambda \cdot P_l{{\varvec{\delta }}} \lambda )$. This sum can be further decomposed as $\sum _{l>j}=\sum _{|j|>l>j}+\sum _{l\geqq |j|}$. By (3.6) we bound the contribution of the first sum by

$$\begin{aligned}&2^{(\frac{d}{2}-\delta )j^-}\Vert \int _0^t e^{(t-s)\Delta }\sum _{|j|>l>j}P_j(P_l\lambda \cdot P_l{{\varvec{\delta }}} \lambda ) \textrm{d}s\Vert _{Y_j}\\&\quad \lesssim \ 2^{(\frac{d}{2}-\delta )j^-} \sum _{|j|>l>j} \Vert P_j(P_l\lambda \cdot P_l{{\varvec{\delta }}} \lambda ) \Vert _{l^1_{|j|}L^\infty L^2}\\&\quad \lesssim \ 2^{(d-\delta )j^-} \sum _{|j|>l>j} \Vert \lambda _{l}\Vert _{l^2_{|j|}L^\infty L^2}\Vert {{\varvec{\delta }}} \lambda _{l}\Vert _{l^2_{|j|}L^\infty L^2}\\&\quad \lesssim \ 2^{(d-2\delta )j^-} \Vert \lambda \Vert _{l^2Z^{s}}{{{\tilde{p}}}}_0. \end{aligned}$$

Next we bound the contribution of the second sum by

$$\begin{aligned} \begin{aligned}&2^{(\frac{d}{2}-\delta )j^-+( s+2)j^+}\Vert \int _0^t e^{(t-s)\Delta }\sum _{l>|j|}P_j(\lambda _{l}{{\varvec{\delta }}} \lambda _l) \textrm{d}s\Vert _{Y_j}\\&\quad \lesssim \ 2^{(\frac{d}{2}-\delta )j^-+( s+2)j^+} \sum _{l>|j|}2^{l-|j|}\Vert \int _0^t e^{(t-s)\Delta } P_j(\lambda _{l}{{\varvec{\delta }}} \lambda _l) \textrm{d}s\Vert _{l^1_{l}L^\infty L^2}\\&\quad \lesssim \ 2^{(\frac{d}{2}-\delta )j^-+( s+2)j^+}\sum _{l>|j|}2^{l-|j|+dj/2-2j^+} \Vert \lambda _{l} \Vert _{l^2_{l}L^\infty L^2}\Vert {{\varvec{\delta }}} \lambda _{l} \Vert _{l^2_{l}L^\infty L^2}\\&\quad \lesssim \ 2^{(d+1-\delta )j^-+( s+\frac{d}{2}-1)j^+} \sum _{l>|j|}2^{l}\Vert \lambda _{l}\Vert _{l^2_{l}L^\infty L^2}\Vert {{\varvec{\delta }}} \lambda _{l}\Vert _{l^2_{l}L^\infty L^2}\\&\quad \lesssim \ 2^{(d+1-\delta )j^-}\Vert \lambda \Vert _{l^2Z^s}{{{\tilde{p}}}}_0+{\textbf{1}}_{>0}(j) \Vert \lambda \Vert _{l^2Z^s}{{{\tilde{p}}}}_j. \end{aligned} \end{aligned}$$

This concludes the proof of the bound (5.23), and completes the proof of the proposition. $\square$

## Multilinear and Nonlinear Estimates

This section contains our main multilinear estimates which are needed for the analysis of the Schrödinger equation in (2.28). We begin with the following low-high bilinear estimates of $\nabla h\nabla \lambda$.

### Proposition 6.1

Let $s>\frac{d}{2}$, $d\geqq 2$ and $k\in {{\mathbb {N}}}$. Suppose that $\nabla a(x)\lesssim \langle x\rangle ^{-1}$, $h\in Y^{s+2}$ and $\lambda _k\in l^2X^s$. Then for $-s\leqq \sigma \leqq s$ we have

6.1 $$\begin{aligned}&\Vert \nabla h_{\leqq k}\cdot \nabla \lambda _k\Vert _{l^2N^{\sigma }}\lesssim \min \{ \Vert h\Vert _{Y^{\sigma +2}}\Vert \lambda _k\Vert _{l^2X^s},\Vert h\Vert _{Y^{s+2}}\Vert \lambda _k\Vert _{l^2X^{\sigma }}\}, \end{aligned}$$

6.2 $$\begin{aligned}&\Vert h_{\leqq k}\nabla a\nabla \lambda _k\Vert _{l^2N^{\sigma }}\lesssim \min \{ \Vert h\Vert _{Y^{\sigma +2}}\Vert \lambda _k\Vert _{l^2X^s},\Vert h\Vert _{Y^{s+2}}\Vert \lambda _k\Vert _{l^2X^{\sigma }}\}. \end{aligned}$$

In addition, if $d/2-2<\sigma \leqq s-1$ then we have

6.3 $$\begin{aligned} \Vert \nabla h_{\leqq k}\cdot \nabla \lambda _k\Vert _{l^2N^{\sigma }}&\lesssim \min \{ \Vert h\Vert _{Z^{\sigma _d,\sigma +2}}\Vert \lambda _k\Vert _{Z^s},\Vert h\Vert _{Z^{\sigma _d,s+2}}\Vert \lambda _k\Vert _{Z^{\sigma }}\}, \end{aligned}$$

and if $d/2-2<\sigma \leqq s-2$ then we have

6.4 $$\begin{aligned} \Vert h_{\leqq k}\nabla ^2\lambda _k\Vert _{l^2N^{\sigma }}&\lesssim \Vert h\Vert _{Z^{\sigma _d,\sigma +2}}\Vert \lambda _k\Vert _{l^2X^s}. \end{aligned}$$

### Proof

*(a) The estimate* (6.1). This is obtained by a Littlewood–Paley decomposition and the following estimate

$$\begin{aligned} \Vert \nabla P_j h\nabla \lambda _k\Vert _{l^2_kN_k}\lesssim 2^{\frac{d}{2}j+2j^+}\Vert P_j h\Vert _{Y_j}\Vert \lambda _k\Vert _{X_k},\qquad j\leqq k,\ j\in {{\mathbb {Z}}},\ k\in {\mathbb {N}}, \end{aligned}$$

which has been proved in [9, Lemma 5.1].

*(b) The estimate* (6.2). Compared to [9, (5.2)], the estimate (6.2) is improved by decomposing physical space dyadically. By duality, it suffices to prove that

6.5 $$\begin{aligned} II_j= & {} \int _0^1\langle P_jh\nabla a\nabla \lambda _k,z_k\rangle \textrm{d}t\nonumber \\\lesssim & {} 2^{dj/2}\log (2+ |j|)\Vert P_j h\Vert _{Y_j}\Vert \lambda _k\Vert _{X_k},\ \ j\leqq k,\ j\in {{\mathbb {Z}}}, \end{aligned}$$

for any $z_k\in l^2_k X_k$ with $\Vert z_k\Vert _{l^2_k X_k}\leqq 1$. For any decomposition $P_jh=\sum _{l\geqq |j|}h_{j,l}$, using the bound $|\nabla a(x)|\lesssim \langle x\rangle ^{-1}$, we consider the two cases $|x|\geqq 2^{l}$ and $|x|<2^{l}$ respectively and then obtain

$$\begin{aligned} II_j&\lesssim \ \sum _{l\geqq |j|} \sup _{\Vert z_k\Vert _{l^2_k X_k}\leqq 1}\sum _{0\leqq l_1\leqq l}\int _0^1\langle h_{j,l}\langle x\rangle ^{-1}{\textbf{1}}_{ [2^{l_1-1},2^{l_1}]}(x)\nabla \lambda _k,z_k\rangle \textrm{d}t\\&\quad +\sum _{l\geqq |j|} \sup _{\Vert z_k\Vert _{l^2_k X_k}\leqq 1}\int _0^1\langle h_{j,l}\langle x\rangle ^{-1}{\textbf{1}}_{>2^{l}}(x)\nabla \lambda _k,z_k\rangle \textrm{d}t\\&= \ II_{j1}+II_{j2}. \end{aligned}$$

By Bernstein’s inequality we bound the first term by

$$\begin{aligned} II_{j1}&\lesssim \ \sum _{l\geqq |j|} \sup _{\Vert z_k\Vert _{l^2_k X_k}\leqq 1}\sum _{0\leqq l_1\leqq l}2^{-l_1}\Vert h_{j,l}\Vert _{L^{\infty }L^{\infty }} \Vert \nabla \lambda _k\Vert _{l^{\infty }_{l_1}L^2L^2}\Vert z_k\Vert _{l^{\infty }_{l_1}L^2L^2}\\&\lesssim \ \sum _{l\geqq |j|} \sum _{0\leqq l_1\leqq l}2^{\frac{dj}{2}}\Vert h_{j,l}\Vert _{L^{\infty }L^{2}} \Vert \lambda _k\Vert _{X_k}\\&\lesssim \ 2^{dj/2}\log (2+ |j|) \sum _{l\geqq |j|} \frac{|l|}{|j|} \Vert h_{j,l}\Vert _{l^1_l L^{\infty }L^2} \Vert \lambda _k\Vert _{X_k}. \end{aligned}$$

The second term is bounded by

$$\begin{aligned} II_{j2}&\lesssim \ \sum _{l\geqq |j|} 2^{-l}\sup _{\Vert z_k\Vert _{l^2_k X_k}\leqq 1}\Vert h_{j,l}\Vert _{l^1_l L^{\infty }L^{\infty }} \Vert \nabla \lambda _k\Vert _{l^{\infty }_{l}L^2L^2}\Vert z_k\Vert _{l^{\infty }_{l}L^2L^2}\\&\lesssim \ \sum _{l\geqq |j|} \Vert h_{j,l}\Vert _{l^1_l L^{\infty }L^{\infty }} \Vert \lambda _k\Vert _{X_k}\\&\lesssim \ 2^{dj/2}\sum _{l\geqq |j|} \Vert h_{j,l}\Vert _{l^1_l L^{\infty }L^2} \Vert \lambda _k\Vert _{X_k}. \end{aligned}$$

Finally we take the infimum over the decompositions of $P_j h$ to get the bound (6.5), which in turn implies the estimate (6.2).

*c) The estimates* (6.3) *and* (6.4). By duality and Sobolev embedding, for any $j<k$ we have

$$\begin{aligned} \Vert P_j h \nabla ^2 \lambda _k\Vert _{l^2 N^\sigma } \lesssim 2^{\sigma k} \Vert P_j h \nabla ^2 \lambda _k\Vert _{L^2L^2} \lesssim 2^{(\sigma +2) k} \Vert |D|^{\sigma _d }P_j h\Vert _{L^\infty H^{d/2-\sigma _d}} \Vert \lambda _k\Vert _{L^2L^2}, \end{aligned}$$

which gives the bound (6.4). We can also obtain the bound (6.3) similarly. This completes the proof of the lemma. $\square$

We next prove the remaining bilinear estimates and trilinear estimates.

### Proposition 6.2

(Nonlinear estimates) Let $s>\frac{d}{2}$ and $d\geqq 2$. Assume that $p_k,\ {{{\tilde{p}}}}_k$, $s_k$ and ${{{\tilde{s}}}}_k$ are admissible frequency envelopes for $\lambda \in Z^s$, $\lambda \in Z^\sigma$, ${{\mathcal {S}}}\in {{\mathcal {E}}}^{s}$ and ${{\mathcal {S}}}\in {{\mathcal {E}}}^{\sigma }$ respectively. Then we have

6.6 $$\begin{aligned} \Vert S_k(B\lambda )\Vert _{l^2N^{s}}\lesssim & {} s_k\Vert \lambda \Vert _{Z^{s}}+p_k\Vert B\Vert _{L^2 H^{s}} , \end{aligned}$$

6.7 $$\begin{aligned} \Vert S_k(A^2\lambda )\Vert _{l^2N^{s}}\lesssim & {} s_k\Vert A\Vert _{Z^{s}}\Vert \lambda \Vert _{Z^{s}}+ p_k\Vert A\Vert _{Z^{s}}^2, \end{aligned}$$

6.8 $$\begin{aligned} \Vert S_k(\lambda ^3)\Vert _{l^2N^{s}}\lesssim & {} p_k \Vert \lambda \Vert _{Z^{s}}^2. \end{aligned}$$

For $-s\leqq \sigma \leqq s$ we have

6.9 $$\begin{aligned} \Vert S_k\nabla (h_{\geqq k-4}\nabla \lambda )\Vert _{l^2N^{\sigma }}\lesssim & {} \min \{{{{\tilde{s}}}}_k\Vert \lambda \Vert _{Z^s},\ {{{\tilde{p}}}}_k\Vert h\Vert _{Z^{\sigma _d,s+2}}\}, \end{aligned}$$

6.10 $$\begin{aligned} \Vert S_k(A_{\geqq k-4}\nabla \lambda )\Vert _{l^2N^{\sigma }}\lesssim & {} \min \{{{{\tilde{s}}}}_k\Vert \lambda \Vert _{Z^{s}},\ {{{\tilde{p}}}}_k\Vert A\Vert _{Z^{s+1}}\}, \end{aligned}$$

and for $-s\leqq \sigma \leqq s-\delta$ we have

6.11 $$\begin{aligned} \Vert S_k(B\lambda )\Vert _{l^2N^{\sigma }}\lesssim & {} \min \{{{{\tilde{s}}}}_k\Vert \lambda \Vert _{Z^{s}},\ {{{\tilde{p}}}}_k\Vert B\Vert _{L^2 H^{s}}\}, \end{aligned}$$

6.12 $$\begin{aligned} \Vert S_k(A^2\lambda )\Vert _{l^2N^{\sigma }}\lesssim & {} \min \{{{{\tilde{s}}}}_k\Vert A\Vert _{Z^{s}}\Vert \lambda \Vert _{Z^{s}},\ {{{\tilde{p}}}}_k\Vert A\Vert _{Z^{s}}^2\}, \end{aligned}$$

6.13 $$\begin{aligned} \Vert S_k(\lambda ^3)\Vert _{l^2N^{\sigma }}\lesssim & {} {{{\tilde{s}}}}_k \Vert \lambda \Vert _{Z^{s}}^2. \end{aligned}$$

If $$-s\leqq \sigma \leqq s-1$$, then

6.14 $$\begin{aligned} \Vert S_k(A_{< k-4}\nabla \lambda )\Vert _{l^2N^{\sigma }}\lesssim p_k\Vert A\Vert _{Z^{\sigma +1}}. \end{aligned}$$

### Proof

We first prove (6.9) and (6.10). These two bounds are proved by Hölder’s inequality and Bernstein’s inequality, here we only prove the first bound in detail. For the high-low case, by duality we have

$$\begin{aligned} \begin{aligned} \sum _{j\leqq k+C}\Vert S_k \nabla ( h_{k}\nabla \lambda _{j})\Vert _{l^2N^{\sigma }}&\lesssim \sum _{j\leqq k+C}2^{\sigma k}\Vert S_k \nabla ( h_{k}\nabla \lambda _{j})\Vert _{L^2L^2}\\&\lesssim \sum _{j\leqq k+C} 2^{(\sigma +1) k}\Vert h_{k}\Vert _{L^2L^2}\Vert \nabla \lambda _{j}\Vert _{L^{\infty }L^{\infty }}\\&\lesssim \sum _{j\leqq k+C} 2^{(\sigma +1) k+(d/2+1)j}\Vert h_k\Vert _{L^2L^2}\Vert \lambda _{j}\Vert _{L^{\infty }L^2}. \end{aligned} \end{aligned}$$

Then by $-s\leqq \sigma \leqq s$ and (3.2), we can bound this by $\min \{{{{\tilde{s}}}}_k\Vert \lambda \Vert _{Z^s},\ {{{\tilde{p}}}}_k\Vert h\Vert _{L^2 H^{s+2}}\}$. For the high-high case, when $\sigma +d/2+1>0$ we have

$$\begin{aligned}&\sum _{j>k}\Vert S_k \nabla ( h_{j}\nabla \lambda _{j})\Vert _{l^2N^{\sigma }}\\&\quad \lesssim \sum _{j_1= j_2+O(1),j_1>k}2^{(\sigma +1) k+dk/2}\Vert S_k ( h_{j_1}\nabla \lambda _{j_2})\Vert _{L^2L^1}\\&\quad \lesssim \sum _{j_1= j_2+O(1),j_1>k}2^{(\sigma +1+d/2+\delta )(k-j_1)+(\sigma +2+d/2+\delta )j_1}\Vert h_{j_1}\Vert _{L^2L^2}\Vert \lambda _{j_2}\Vert _{L^{\infty }L^2}\\&\quad \lesssim \min \{{{{\tilde{s}}}}_k\Vert \lambda \Vert _{Z^s},{{{\tilde{p}}}}_k\Vert h\Vert _{Z^{1,s+2}}\}, \end{aligned}$$

and when $\sigma +d/2+1\leqq 0$ we have

$$\begin{aligned}&\sum _{j_1= j_2+O(1),j_1>k}\Vert S_k \nabla ( h_{j_1}\nabla \lambda _{j_2})\Vert _{l^2N^{\sigma }}\\&\quad \lesssim \sum _{j_1= j_2+O(1),j_1>k}2^{(\sigma +1+d/2-\delta )k+(\delta +1) j_1}\Vert h_{j_1}\Vert _{L^2L^2}\Vert \lambda _{j_2}\Vert _{L^{\infty }L^2}\\&\quad \lesssim \min \{{{{\tilde{s}}}}_k\Vert \lambda \Vert _{Z^s},{{{\tilde{p}}}}_k\Vert h\Vert _{Z^{1,s+2}}\}. \end{aligned}$$

Next, we prove the bounds (6.6)–(6.8) and (6.11)–(6.13). These are all similar, so we only prove (6.6) and (6.11) in detail. Indeed, by duality we have

$$\begin{aligned} \Vert S_k(B\lambda )\Vert _{l^2N^{\sigma }}\lesssim 2^{\sigma k}\Vert S_k(B\lambda )\Vert _{L^2L^2}. \end{aligned}$$

Then using the Littlewood–Paley trichotomy we divide this into *low-high*, *high-low* and *high-high* cases. For the low-high interactions, by Sobolev embeddings we have for $-s\leqq \sigma \leqq s$

$$\begin{aligned} 2^{\sigma k}\Vert S_k(B_{<k}\lambda _k)\Vert _{L^2L^2}&\lesssim \Vert B_{<k}\Vert _{L^2L^{\infty }}2^{\sigma k}\Vert \lambda _k\Vert _{L^{\infty }L^2}\lesssim {{{\tilde{p}}}}_k\Vert B\Vert _{L^2H^{s}}. \end{aligned}$$

If $-s\leqq \sigma \leqq s-\delta$, we use $L^2 H^\sigma$ for $B_l=S_l B$. Then by $\Vert B_l\Vert _{L^2H^\sigma }\lesssim 2^{\delta (k-l)}{{{\tilde{s}}}}_k$ we also have

$$\begin{aligned} 2^{\sigma k}\Vert S_k(B_{<k}\lambda _k)\Vert _{L^2L^2}\lesssim {{{\tilde{s}}}}_k \Vert \lambda \Vert _{Z^{s}}. \end{aligned}$$

The high-low interactions can be estimated similarly. For the high-high interactions, by Sobolev embeddings when $-d/2-\delta \leqq \sigma \leqq s$ we have

$$\begin{aligned} 2^{\sigma k}\Vert \sum _{l>k} S_k(B_l\lambda _l)\Vert _{L^2L^2}&\lesssim \sum _{l>k} 2^{(\sigma +d/2+2\delta )(k-l)} 2^{(\sigma +d/2+2\delta )l} \Vert B_l\Vert _{L^2L^2}\Vert \lambda _l\Vert _{L^{\infty }L^2}\\&\lesssim \min \{{{{\tilde{s}}}}_k\Vert \lambda \Vert _{Z^s},{{{\tilde{p}}}}_k\Vert B\Vert _{L^2 H^s} \}, \end{aligned}$$

and when $-s\leqq \sigma <-d/2-\delta$ we have

$$\begin{aligned} 2^{\sigma k}\Vert \sum _{l>k} S_k(B_l\lambda _l)\Vert _{L^2L^2}&\lesssim \sum _{l>k} 2^{(\sigma +d/2)k} \Vert B_l\Vert _{L^2L^2}\Vert \lambda _l\Vert _{L^{\infty }L^2}\\&\lesssim 2^{-\delta k} \min \{\Vert B\Vert _{L^2H^\sigma }\Vert \lambda \Vert _{Z^{-\sigma }},\Vert B\Vert _{L^2H^{-\sigma }}\Vert \lambda \Vert _{Z^{\sigma }}\}\\&\lesssim \min \{{{{\tilde{s}}}}_k\Vert \lambda \Vert _{Z^s},{{{\tilde{p}}}}_k\Vert B\Vert _{L^2 H^s} \}, \end{aligned}$$

These imply the bounds (6.6) and (6.11).

Finally, we prove the bound (6.14). If $d/2-1+\delta \leqq \sigma \leqq s-1$, by duality and Sobolev embeddings, we have

$$\begin{aligned} \Vert A_{<k}\nabla \lambda _k\Vert _{l^2N^\sigma }\lesssim 2^{(\sigma +1) k}\Vert A_{<k} \Vert _{L^2L^\infty } \Vert \lambda _k\Vert _{L^\infty L^2}\lesssim p_k \Vert A\Vert _{Z^{\sigma +1}}. \end{aligned}$$

If $\sigma < d/2-1+\delta$, we have

$$\begin{aligned} 2^{\sigma k}\Vert A_{<k}\nabla \lambda _k\Vert _{L^2L^2}&\lesssim \sum _{0\leqq l<k} 2^{(d/2-1-\sigma +2\delta )(l-k)}\Vert A\Vert _{L^2 H^{\sigma +1}} 2^{(d/2+2\delta )k} \Vert \lambda _k\Vert _{L^\infty L^2}\\&\lesssim \ p_k\Vert A\Vert _{Z^{\sigma +1}}. \end{aligned}$$

Then the bound (6.14) follows. Hence this completes the proof of the lemma. $\square$

We shall also require the following bounds for commutators.

### Proposition 6.3

(Commutator bounds) Let $d\geqq 2$ and $s>\frac{d}{2}$. Let *m*(*D*) be a multiplier with symbol $m\in S^0$. Assume $h\in Y^{s+2}$, $\partial _x A\in L^2 H^s$ and $\lambda _k \in l^2X^s$, frequency localized at frequency $2^k$. If $-s\leqq \sigma \leqq s$ then we have

6.15 $$\begin{aligned} \Vert \nabla [S_{<k-4}h,m(D)]\nabla \lambda _k\Vert _{l^2N^{\sigma }}\lesssim & {} \min \{ \Vert h\Vert _{Y^{\sigma +2}}\Vert \lambda _k\Vert _{l^2X^s},\Vert h\Vert _{Y^{s+2}}\Vert \lambda _k\Vert _{l^2X^{\sigma }}\},\nonumber \\ \end{aligned}$$

6.16 $$\begin{aligned} \Vert [S_k,A_{<k-4}]\nabla \lambda _k\Vert _{l^2N^{\sigma }}\lesssim & {} \min \{\Vert \partial _x A\Vert _{L^2H^{s}}\Vert \lambda _k\Vert _{L^\infty H^\sigma },\nonumber \\{} & {} \Vert \partial _x A\Vert _{L^2 H^{\sigma }}\Vert \lambda _k\Vert _{L^\infty H^s}\}. \end{aligned}$$

### Proof

This is similar to Proposition 5.3 in [9]. First we estimate (6.15). In [20, Proposition 3.2], it was shown that

$$\begin{aligned} \nabla [S_{<k-4}g,m(D)]\nabla S_k \lambda =L(\nabla S_{<k-4}g,\nabla S_k \lambda ), \end{aligned}$$

where *L* is a translation invariant operator satisfying

$$\begin{aligned} L(f,g)(x)=\int f(x+y)g(x+z){\tilde{m}}(y+z)\ dydz,\ \ \ {\tilde{m}}\in L^1. \end{aligned}$$

Given this representation, as we are working in translation-invariant spaces, by (6.1) the bound (6.15) follows.

Next, for the bound (6.16). Since

$$\begin{aligned}{}[S_k,A_{<k}]\nabla \lambda =\int _0^1 \int 2^{kd}\check{\varphi }(2^k y)2^k y \nabla A_{<k}(x-sy) 2^{-k}\nabla \lambda _{[k-3,k+3]}(x-y)\ dyds, \end{aligned}$$

By translation-invariance and the similar argument to (6.11), the bound (6.16) follows. This completes the proof of the lemma. $\square$

## Local Energy Decay and the Linearized Problem

In this section, we consider a linear Schrödinger equation

7.1 $$\begin{aligned} \left\{ \begin{aligned}&i\partial _t \lambda +\partial _{\alpha }(g^{\alpha {\beta }}\partial _{{\beta }}\lambda )+2i A^{\alpha }\partial _{\alpha }\lambda =F,\\&\lambda (0)=\lambda _0, \end{aligned}\right. \end{aligned}$$

and, under suitable assumptions on the coefficients, we prove that the solution satisfies suitable energy and local energy bounds.

### The Linear Paradifferential Schrödinger Flow

As an intermediate step, here we prove energy and local energy bounds for a frequency localized linear paradifferential Schrödinger equation

7.2 $$\begin{aligned} i\partial _t\lambda _k+\partial _{\alpha }(g^{\alpha {\beta }}_{<k-4}\partial _{{\beta }}\lambda _k)+2iA^{\alpha }_{<k-4}\partial _{\alpha }\lambda _k=f_k. \end{aligned}$$

We begin with the energy estimates, which are fairly standard.

#### Lemma 7.1

(Energy-type estimate) Let $d\geqq 2$. Assume that $\lambda _k$ solves the equation (7.2) with initial data $\lambda _k(0)$ in the time interval [0, 1]. For a fixed $s>\frac{d}{2}$, assume that $\partial _x A\in L^2 H^{s}$, $\lambda _k\in l^2_kX_k$, and $f_k=f_{1k}+f_{2k}$ with $f_{1k}\in N$ and $f_{2k}\in L^1L^2$. Then we have

7.3 $$\begin{aligned} \begin{aligned} \Vert \lambda _k\Vert _{L^{\infty }_tL^2_x}^2&\lesssim \ \Vert \lambda _k(0)\Vert _{L^2}^2+\Vert \partial _x A\Vert _{L^2 H^{s}}\Vert \lambda _k\Vert _{X_k}^2 \\&\quad +\Vert \lambda _k\Vert _{X_k}\Vert f_{1k}\Vert _{N_k} +\Vert \lambda _k\Vert _{L^{\infty }L^2}\Vert f_{2k}\Vert _{L^1L^2}. \end{aligned} \end{aligned}$$

#### Proof

For the proof, we refer the readers to Lemma 6.1 in [9]. Here we just replace the condition $A\in Z^{1,s+1}$ in [9] by the assumption $\partial _x A\in L^2H^s$. $\square$

Next, we prove the main result of this section, namely the local energy estimates for solutions to (7.2).

#### Proposition 7.2

(Local energy decay) Let $d\geqq 2$. Assume that the coefficients $h=g-I_d$ and *A* in (7.2) satisfy

7.4 $$\begin{aligned} \Vert h\Vert _{{\textbf{Y}}^{s+2}}+ \Vert A\Vert _{Z^{s+1}}\lesssim \epsilon \end{aligned}$$

for some $s>\frac{d}{2}$ and $\epsilon >0$ small. Let $\lambda _k$ be a solution to (7.2) which is localized at frequency $2^k$. Then the following estimate holds:

7.5 $$\begin{aligned} \Vert \lambda _k\Vert _{l^2_k X_k}\lesssim \Vert \lambda _{0k}\Vert _{L^2}+\Vert f_k\Vert _{l^2_kN_k}. \end{aligned}$$

#### Proof

The proof is closely related to that given in [9, 20, 21]. However, here the metric $g=I_d+h$ and magnetic potential *A* will satisfy some parabolic equations, so we need to modify the assumptions both on *h* and *A* to match our main results.

As an intermediate step in the proof, we will establish a local energy decay bound in a cube $Q\in {{\mathcal {Q}}}_l$ with $0\leqq l\leqq k$:

7.6 $$\begin{aligned} \begin{aligned} 2^{k-l}\Vert \lambda _k\Vert _{L^2L^2([0,1]\times Q)}^2&\lesssim \ \Vert \lambda _k\Vert _{L^{\infty }L^2}^2+ \Vert f_k\Vert _{N_k} \Vert \lambda _k \Vert _{X_k} \\&\quad +(2^{-k}+\Vert A\Vert _{Z^{1-\delta ,s+1}}+\Vert h\Vert _{{\textbf{Y}}^{s+2}})\Vert \lambda _k\Vert _{l^2_kX_k}^2. \end{aligned} \end{aligned}$$

The proof of this bound is based on a positive commutator argument using a well chosen multiplier ${{\mathcal {M}}}$. This will be first-order differential operator with smooth coefficients which are localized at frequency $\lesssim 1$. Precisely, we will use a multiplier ${{\mathcal {M}}}$ which is a self-adjoint differential operator having the form

7.7 $$\begin{aligned} i2^k{{\mathcal {M}}}=a^{\alpha }(x)\partial _{\alpha }+\partial _{\alpha }a^{\alpha }(x), \end{aligned}$$

with uniform bounds on *a* and its derivatives.

Before proving (7.5), we need the following lemma which is used to dismiss the $(g-I)$ contribution to the commutator $[\partial _{\alpha }g^{\alpha {\beta }}\partial _{{\beta }},{{\mathcal {M}}}]$:

#### Lemma 7.3

Let $d\geqq 2$ and $s>\frac{d}{2}$. Assume that $h\in {\textbf{Y}}^{s+2}$, $A\in Z^{1-\delta ,s+1}$ and $\lambda \in l^2_kX_k$, and let ${{\mathcal {M}}}$ be as (7.7). Then we have

7.8 $$\begin{aligned}{} & {} \int _0^1\langle [\partial _{\alpha }h^{\alpha {\beta }}_{\leqq k}\partial _{{\beta }},{{\mathcal {M}}}]\lambda _k,\lambda _k\rangle \textrm{d}t\lesssim \Vert h\Vert _{{\textbf{Y}}^{s+2}}\Vert \lambda _k\Vert _{l^2_kX_k}^2, \end{aligned}$$

7.9 $$\begin{aligned}{} & {} \int _0^1\mathop {\textrm{Re}}\nolimits \langle A^{\alpha }_{<k-4}\partial _{\alpha }\lambda _k,{{\mathcal {M}}}\lambda _k \rangle \textrm{d}t\lesssim \Vert A\Vert _{Z^{1-\delta ,s+1}}\Vert \lambda _k\Vert _{X_k}^2. \end{aligned}$$

#### Proof of Lemma 7.3

By (7.7) and direct computations, we get

$$\begin{aligned} {[}\partial _{\alpha }h^{\alpha {\beta }}\partial _{{\beta }},{{\mathcal {M}}}]\approx 2^{-k}[\nabla (h\nabla a+a\nabla h)\nabla +\nabla h\nabla ^2 a+h\nabla ^3 a]. \end{aligned}$$

Then it suffices to estimate

$$\begin{aligned} 2^{-k} \int _0^1\langle (h_{\leqq k}\nabla a+a\nabla h_{\leqq k})\nabla \lambda _k,\nabla \lambda _k\rangle \textrm{d}t+2^{-k}\int _0^1\langle (\nabla h_{\leqq k}\nabla ^2 a+h_{\leqq k}\nabla ^3 a)\lambda _k,\lambda _k\rangle \textrm{d}t. \end{aligned}$$

The first integral is estimated by (6.1) and (6.2), while the second integral is bounded by Sobolev embeddings. Hence, the bound (7.8) follows.

For the second bound (7.9), by (7.7) and integration by parts we rewrite the left-hand side of (7.9) and bound it by

$$\begin{aligned} \mathop {\textrm{Re}}\nolimits \int _0^1\langle A^{\alpha }_{<k-4}\partial _{\alpha }\lambda _k,{{\mathcal {M}}}\lambda _k \rangle \textrm{d}t&\lesssim 2^{-k} \int _0^1\int _{{{\mathbb {R}}}^d} |\langle \nabla \rangle A_{<k} \lambda _k \nabla \lambda _k |\textrm{d}x \textrm{d}t\\&\lesssim \Vert |\nabla |^{1-\delta } A\Vert _{L^2H^{s+\delta }}\Vert \lambda _k\Vert _{L^\infty L^2}^2. \end{aligned}$$

This implies the bound (7.9), and hence completes the proof of the lemma. $\square$

Returning to the proof of (7.6), for the self-adjoint multiplier ${{\mathcal {M}}}$ we compute

$$\begin{aligned} \frac{\textrm{d}}{\textrm{d}t}\langle \lambda _k,{{\mathcal {M}}}\lambda _k\rangle&=2\mathop {\textrm{Re}}\nolimits \langle \partial _t\lambda _k,{{\mathcal {M}}}\lambda _k\rangle \\&=2\mathop {\textrm{Re}}\nolimits \langle i\partial _{\alpha }(g^{\alpha {\beta }}_{<k-4}\partial _{{\beta }}\lambda _k)-2A^{\alpha }_{<k-4}\partial _{\alpha }\lambda _k-if_k,{{\mathcal {M}}}\lambda _k \rangle \\&=i\langle [-\partial _{\alpha }g^{\alpha {\beta }}_{<k-4}\partial _{{\beta }},{{\mathcal {M}}}]\lambda _k,\lambda _k\rangle +2\mathop {\textrm{Re}}\nolimits \langle -2A^{\alpha }_{<k-4}\partial _{\alpha }\lambda _k-if_k,{{\mathcal {M}}}\lambda _k \rangle \end{aligned}$$

We then use the multiplier ${{\mathcal {M}}}$ as in [20, 21] so that the following three properties hold:

1. Boundedness on frequency $2^k$ localized functions,
  $$\begin{aligned} \Vert {{\mathcal {M}}}u\Vert _{L^2_x}\lesssim \Vert u\Vert _{L^2_x}. \end{aligned}$$
2. Boundedness in *X*,
  $$\begin{aligned} \Vert {{\mathcal {M}}}u\Vert _{X}\lesssim \Vert u\Vert _{X}. \end{aligned}$$
3. Positive commutator,
  $$\begin{aligned} i\langle [-\partial _{\alpha }g^{\alpha {\beta }}_{<k-4}\partial _{{\beta }},{{\mathcal {M}}}]u,u\rangle \gtrsim 2^{k-l}\Vert u\Vert ^2_{L^2_{t,x}([0,1]\times Q)}\!-\!O(2^{-k}\!+\!\Vert h\Vert _{{\textbf{Y}}^{s+2}})\Vert u\Vert _{l^2_kX_k}^2. \end{aligned}$$

If these three properties hold for $u=\lambda _k$, then by (7.9) and (7.4) the bound (7.6) follows.

We first do this when the Fourier transform of the solution $\lambda _k$ is restricted to a small angle

7.10 $$\begin{aligned} \text {supp}\ {\widehat{\lambda }}_k\subset \{|\xi |\lesssim \xi _1\}. \end{aligned}$$

Without loss of generality due to translation invariance, $Q=\{|x_j|\leqq 2^l:j=1,\ldots ,d\}$, and we set *m* to be a smooth, bounded, increasing function such that $m'(s)=\varphi ^2(s)$ where $\varphi$ is a Schwartz function localized at frequencies $\lesssim 1$, and $\varphi \approx 1$ for $|s|\leqq 1$. We rescale *m* and set $m_l(s)=m(2^{-l}s)$. Then, we fix

$$\begin{aligned} {{\mathcal {M}}}=\frac{1}{i2^k} (m_l(x_1)\partial _1+\partial _1 m_l(x_1)). \end{aligned}$$

The properties (1) and (2) are immediate due to the frequency localization of $u=\lambda _k$ and $m_l$ as well as the boundedness of $m_l$. By (7.8) it suffices to consider the property (3) for the operator

$$\begin{aligned} -\Delta =-\partial _{\alpha }g^{\alpha {\beta }}_{<k-4}\partial _{{\beta }}+\partial _{\alpha }h^{\alpha {\beta }}_{<k-4}\partial _{{\beta }}. \end{aligned}$$

This yields

$$\begin{aligned} i2^k[-\Delta ,{{\mathcal {M}}}]=-2^{-l+2}\partial _1 \varphi ^2(2^{-l}x_1)\partial _1+O(1), \end{aligned}$$

and hence

$$\begin{aligned} i2^k\langle [-\Delta ,{{\mathcal {M}}}]\lambda _k,\lambda _k\rangle =2^{-l+2}\Vert \varphi (2^{-l}x_1)\partial _1\lambda _k\Vert _{L^2L^2}^2+O(\Vert \lambda _k\Vert _{L^2L^2}^2) \end{aligned}$$

Utilizing our assumption (7.10), it follows that

$$\begin{aligned} 2^{k-l}\Vert \varphi (2^{-l}x_1)\lambda _k\Vert _{L^2L^2}^2\lesssim i\langle [-\Delta ,{{\mathcal {M}}}]\lambda _k,\lambda _k\rangle +2^{-k}O(\Vert \lambda _k\Vert _{L^2L^2}^2) \end{aligned}$$

which yields (3) when combined with (7.8).

We proceed to reduce the problem to the case when (7.10) holds. We let $\{ \theta _j (\omega ) \}_{j=1}^d$ be a partition of unity,

$$\begin{aligned} \sum _{j}\theta _j(\omega )=1,\ \ \ \ \omega \in {{\mathbb {S}}}^{d-1}, \end{aligned}$$

where $\theta _j(\omega )$ is supported in a small angle about the *j*-th coordinate axis. Then, we can set $\lambda _{k,j}=\Theta _{k,j}\lambda _k$ where

$$\begin{aligned} {{\mathcal {F}}}\Theta _{k,j}\lambda =\theta _j(\frac{\xi }{|\xi |})\sum _{k-1\leqq l\leqq k+1}\varphi _l(\xi ){\widehat{\lambda }}(t,\xi ). \end{aligned}$$

We see that

$$\begin{aligned}&(i\partial _t+\partial _{\alpha }g^{\alpha {\beta }}_{<k-4}\partial _{{\beta }})\lambda _{k,j}+2iA^{\alpha }_{<k-4}\partial _{\alpha }\lambda _{k,j}\\&\quad =\Theta _{k,j}f_k-\partial _{\alpha }[\Theta _{k,j},g^{\alpha {\beta }}_{<k-4}]\partial _{{\beta }}\lambda _k-2i[\Theta _{k,j},A^{\alpha }_{\leqq k-4}]\partial _{\alpha }\lambda _k. \end{aligned}$$

By applying ${{\mathcal {M}}}$, suitably adapted to the correct coordinate axis, to $\lambda _{k,j}$ and summing over *j*, we obtain

$$\begin{aligned}&2^{k-l}\Vert \lambda _k\Vert _{L^2L^2([0,1]\times Q)}^2\\&\quad \lesssim \ \Vert \lambda _k\Vert _{L^{\infty }L^2}^2+\sum _{j=1}^d\int _0^1\langle -\Theta _{k,j}f_k,{{\mathcal {M}}}\lambda _{k,j}\rangle \textrm{d}s\\&\qquad +\sum _{j=1}^d\int \langle [\Theta _{k,j},\partial _{\alpha }g^{\alpha {\beta }}_{<k-4}\partial _{{\beta }}]\lambda _k+[\Theta _{k,j},2iA^{\alpha }_{<k-4}]\partial _{\alpha }\lambda _k,{{\mathcal {M}}}\lambda _{k,j}\rangle \textrm{d}s\\&\qquad +(2^{-k}+\Vert |D|^{1-\delta } A\Vert _{L^2 H^{s+\delta }}+\Vert h\Vert _{{\textbf{Y}}^{s+2}})\Vert \lambda _k\Vert _{l^2_kX_k}^2\\&\quad \lesssim \ \Vert \lambda _k\Vert _{L^{\infty }L^2}^2+\Vert f_k\Vert _{N_k}\Vert \lambda _k\Vert _{X_k} +(2^{-k}+\Vert |D|^{1-\delta } A\Vert _{L^2 H^{s+\delta }}+\Vert h\Vert _{{\textbf{Y}}^{s+2}})\Vert \lambda _k\Vert _{l^2_kX_k}^2. \end{aligned}$$

The commutator is done via (6.15) and (6.16). Then (7.6) follows.

Next we use the bound (7.6) to complete the proof of Proposition 7.2. Taking the supremum in (7.6) over $Q\in {{\mathcal {Q}}}_l$ and over *l*, we obtain

$$\begin{aligned} \begin{aligned} 2^k\Vert \lambda _k\Vert _{X}^2&\lesssim \ \Vert \lambda _k\Vert _{L^{\infty }L^2}^2+\Vert f_{1k}\Vert _{N_k}\Vert \lambda _k\Vert _{X_k}+\Vert f_{2k}\Vert _{L^1L^2}\Vert \lambda _k\Vert _{L^{\infty }L^2}\\&\quad +(2^{-k}+\Vert |D|^{1-\delta } A\Vert _{L^2 H^{s+\delta }}+\Vert h\Vert _{{\textbf{Y}}^{s+2}})\Vert \lambda _k\Vert _{l^2_kX_k}^2\\&\lesssim \ \Vert \lambda _k\Vert _{L^{\infty }L^2}^2+\Vert f_{1k}\Vert _{N_k}\Vert \lambda _k\Vert _{X_k}+\Vert f_{2k}\Vert _{L^1L^2}^2\\&\quad +(2^{-k}+\Vert |D|^{1-\delta } A\Vert _{L^2 H^{s+\delta }}+\Vert h\Vert _{{\textbf{Y}}^{s+2}})\Vert \lambda _k\Vert _{l^2_kX_k}^2. \end{aligned} \end{aligned}$$

Combined with (7.3), we get

7.11 $$\begin{aligned} \begin{aligned} \Vert \lambda _k\Vert _{X_k}^2&\lesssim \ \Vert \lambda _k(0)\Vert _{L^2}^2 +\Vert f_{1k}\Vert _{N_k}^2+\Vert f_{2k}\Vert _{L^1L^2}^2\\&\quad +(2^{-k}+\Vert |D|^{1-\delta } A\Vert _{L^2 H^{s+\delta }}+\Vert h\Vert _{{\textbf{Y}}^{s+2}})\Vert \lambda _k\Vert _{l^2_kX_k}^2. \end{aligned} \end{aligned}$$

We now finish the proof by incorporating the summation over cubes. We let $\{\chi _Q\}$ denote a partition via functions which are localized to frequencies $\lesssim 1$ which are associated to cubes *Q* of scale $M2^k$. We also assume that $|\nabla ^l\chi _Q|\lesssim (2^k M)^{-l}$, $l=1,2$. Thus,

$$\begin{aligned}&(i\partial _t+\partial _{\alpha }g^{\alpha {\beta }}_{<k-4}\partial _{{\beta }})\chi _Q \lambda _k+2iA^{\alpha }_{<k-4}\partial _{\alpha }\chi _Q \lambda _k\\&\quad = \chi _Q f_k+[\partial _{\alpha }g^{\alpha {\beta }}_{<k-4}\partial _{{\beta }},\chi _Q]\lambda _k+2iA^{\alpha }_{<k-4}\partial _{\alpha }\chi _Q\cdot \lambda _k \end{aligned}$$

Applying (7.3) to $\chi _Q\lambda _k$, we obtain

$$\begin{aligned}&\sum _Q \Vert \chi _Q\lambda _k\Vert _{L^{\infty }L^2}^2\\&\quad \lesssim \sum _Q \Vert \chi _Q\lambda _k(0)\Vert _{L^2}^2 +\Vert \partial _x A\Vert _{L^2 H^{s}}\sum _Q\Vert \chi _Q\lambda _k\Vert _{X_k}^2\\&\qquad +\Big (\sum _Q\Vert \chi _Qf_k\Vert _{N_k}^2\Big )^{1/2}\Big (\sum _Q\Vert \chi _Q\lambda _k\Vert _{X_k}^2\Big )^{1/2}\\&\qquad +\sum _Q\Vert [\partial _{\alpha }g^{\alpha {\beta }}_{<k-4}\partial _{{\beta }},\chi _Q]\lambda _k+2iA^{\alpha }_{<k-4}\partial _{\alpha }\chi _Q\cdot \lambda _k\Vert _{L^1L^2}^2. \end{aligned}$$

But by (7.4) we have

7.12 $$\begin{aligned} \begin{aligned} \sum _Q\Vert [\nabla g\nabla ,\chi _Q]\lambda _k \Vert _{L^1L^2}^2&\lesssim \sum _Q\Vert \nabla g\cdot \nabla \chi _Q\cdot \lambda _k+g\nabla (\nabla \chi _Q\cdot \lambda _k)\Vert _{L^1L^2}^2\\&\lesssim \ (1+\Vert |D|^{\sigma _d} h\Vert _{L^\infty H^{s+1-\sigma _d}}) M^{-2}\sum _Q \Vert \chi _Q\lambda _k\Vert _{L^{\infty }L^2}^2, \end{aligned} \end{aligned}$$

and also

7.13 $$\begin{aligned} \sum _Q\Vert 2iA^{\alpha }_{<k-4}\partial _{\alpha }\chi _Q\cdot \lambda _k\Vert _{L^1L^2}^2\lesssim (1+\Vert |D|^{1-\delta } A\Vert _{L^2 H^{s+\delta }}) M^{-2}\sum _Q \Vert \chi _Q\lambda _k\Vert _{L^{\infty }L^2}^2. \end{aligned}$$

For *M* sufficiently large, we can bootstrap the commutator terms, and, after a straightforward transition to cubes of scale $2^k$ rather than $M2^k$, we observe that

7.14 $$\begin{aligned} \Vert \lambda _k\Vert _{l^2_kL^{\infty }L^2}^2 \lesssim \Vert \lambda _k(0)\Vert _{L^2}^2 +\Vert |D|^{1-\delta } A\Vert _{L^2 H^{s+\delta }}\Vert \lambda _k\Vert _{l_k^2X_k}^2 +\Vert f_k\Vert _{l^2_kN_k}\Vert \lambda _k\Vert _{l^2_kX_k}.\nonumber \\ \end{aligned}$$

We now apply (7.11) to $\chi _Q\lambda _k$, and then by (7.12) and (7.13) we see that

$$\begin{aligned} \sum _Q \Vert \chi _Q \lambda _k\Vert _{X_k}^2&\lesssim \Vert \lambda _k(0)\Vert _{L^2}^2+\sum _Q\Vert \chi _Q f_k\Vert _{N_k}^2+M^{-2}\sum _Q\Vert \chi _Q\lambda _k\Vert _{X_k}^2\\&\quad +(2^{-k}+\Vert h\Vert _{{\textbf{Y}}^{s+2}}+\Vert |D|^{1-\delta } A\Vert _{L^2 H^{s+\delta }})\sum _Q\Vert \chi _Q\lambda _k\Vert _{l^2_kX_k}^2. \end{aligned}$$

For $M\gg 1$, we have

$$\begin{aligned} M^{-d}\Vert \lambda _k\Vert _{l^2_kX_k}^2\lesssim \Vert \lambda _k(0)\Vert _{L^2}^2+\Vert f_k\Vert _{l^2_kN_k}^2 +(2^{-k}+\Vert h\Vert _{{\textbf{Y}}^{s+2}}+\Vert |D|^{1-\delta } A\Vert _{L^2 H^{s+\delta }})\Vert \lambda _k\Vert _{l^2_kX_k}^2. \end{aligned}$$

By (7.4), for *k* sufficiently large (depending on *M*), we may absorb the the last term in the right-hand side into the left, i.e

$$\begin{aligned} \Vert \lambda _k\Vert _{l^2_kX_k}^2\lesssim \Vert \lambda _k(0)\Vert _{L^2}^2+\Vert f_k\Vert _{l^2_kN_k}^2. \end{aligned}$$

On the other hand, for the remaining bounded range of *k*, we have

$$\begin{aligned} \Vert \lambda \Vert _{X_k}\lesssim \Vert \lambda \Vert _{L^{\infty }L^2}, \end{aligned}$$

and then (7.14) and (7.4) give

$$\begin{aligned} \Vert \lambda _k\Vert _{l^2_kX_k}^2&\lesssim \ \Vert \lambda _k(0)\Vert _{L^2}^2 +\Vert |D|^{1-\delta }A\Vert _{L^2H^{s+\delta }}\Vert \lambda _k\Vert _{l_k^2X_k}^2 +\Vert f_k\Vert _{l^2_kN_k}\Vert \lambda _k\Vert _{l^2_kX_k}\\&\lesssim \ \Vert \lambda _k(0)\Vert _{L^2}^2 +\Vert f_k\Vert _{l^2_kN_k}^2, \end{aligned}$$

which finishes the proof of (7.5). $\square$

### The Full Linear Problem

Here we use the bounds for the paradifferential equation in the previous subsection in order to prove similar bounds for the full equation (7.1).

#### Proposition 7.4

(Well-posedness) Let $s>\frac{d}{2}$, $d\geqq 2$ and $h=g-I_d$. Assume that the metric *g* and the magnetic potential *A* satisfy

$$\begin{aligned} \Vert h\Vert _{{\textbf{Y}}^{s+2}},\ \Vert |D|^{1-\delta } A\Vert _{L^2 H^{s+\delta }}\ll 1. \end{aligned}$$

Then the equation (7.1) is well-posed for initial data $\lambda _0\in H^{\sigma }$ with $-s\leqq \sigma \leqq s$, and we have the estimate

7.15 $$\begin{aligned} \Vert \lambda \Vert _{l^2X^{\sigma }}\lesssim \Vert \lambda _0\Vert _{H^{\sigma }}+\Vert F\Vert _{l^2N^{\sigma }}. \end{aligned}$$

#### Proof

The well-posedness follows in a standard fashion from a similar energy estimate for the adjoint equation. Since the adjoint equation has a similar form, with similar bounds on the coefficients, such an estimate follows directly from (7.15). Thus, we now focus on the proof of the bound (7.15). For $\lambda$ solving (7.1), we see that $\lambda _k$ solves

$$\begin{aligned} \left\{ \begin{aligned}&i\partial _t \lambda _k+\partial _{\alpha }(g^{\alpha {\beta }}_{<k-4}\partial _{{\beta }}\lambda _k)+2iA^{\alpha }_{<k-4}\partial _{\alpha }\lambda _k=F_k+H_k,\\&\lambda _k(0)=\lambda _{0k}, \end{aligned}\right. \end{aligned}$$

where $$F_k:=S_k F$$ and

$$\begin{aligned} H_k:&=-S_k\partial _{\alpha }(g^{\alpha {\beta }}_{\geqq k-4}\partial _{{\beta }}\lambda )-\partial _{\alpha }[S_k,g^{\alpha {\beta }}_{< k-4}]\partial _{{\beta }}\lambda -2i[S_k,A^{\alpha }_{<k-4}]\partial _{\alpha }\lambda \\&\quad -2iS_k( A^{\alpha }_{\geqq k-4}\partial _{\alpha }\lambda ). \end{aligned}$$

If we apply Proposition 7.2 to each of these equations, we see that

$$\begin{aligned} \Vert \lambda _k\Vert _{l^2X^{\sigma }}^2\lesssim \Vert \lambda _{0k}\Vert _{H^{\sigma }}^2+\Vert F_k\Vert _{l^2N^{\sigma }}^2+\Vert H_k\Vert _{l^2N^{\sigma }}^2. \end{aligned}$$

We claim that

7.16 $$\begin{aligned} \sum _{k}\Vert H_k\Vert _{l^2N^{\sigma }}^2\lesssim (\Vert h\Vert _{{\textbf{Y}}^{s+2}}+\Vert \partial _x A\Vert _{L^2 H^{s}})^2\Vert \lambda \Vert _{l^2X^{\sigma }}^2,\ \text {for }-s\leqq \sigma \leqq s.\qquad \end{aligned}$$

Indeed, the bound for the terms in $H_k$ follows from (6.9), (6.15), (6.16) and (6.10), respectively. Then by the above two bounds and the smallness of *h* and *A*, we obtain the estimate (7.15). $\square$

### The Linearized Problem

Here we consider the linearized equation

7.17 $$\begin{aligned} \left\{ \begin{aligned}&i\partial _t \Lambda +\partial _{\alpha }(g^{\alpha {\beta }}\partial _{{\beta }}\Lambda )+2iA^{\alpha }\partial _{\alpha }\Lambda =F+G,\\&\Lambda (0)=\Lambda _0, \end{aligned}\right. \end{aligned}$$

where

$$\begin{aligned} G=-\nabla ({\mathcal {G}}\nabla \lambda )-2i{\mathcal {A}}^{\alpha }\partial _{\alpha } \lambda , \end{aligned}$$

and we prove the following:

#### Proposition 7.5

Let $s>\frac{d}{2}$, $\frac{d}{2}-2<\sigma \leqq s-2$, $d\geqq 2$ and $h=g-I_d\in {\textbf{Y}}^{s+2}$, assume that $\Lambda$ is a solution of (7.17), the metric *g* and *A* satisfy

$$\begin{aligned} \Vert h\Vert _{{\textbf{Y}}^{s+2}},\ \Vert |D|^{1-\delta }A\Vert _{L^2 H^{s+\delta }}\ll 1. \end{aligned}$$

Then we have the estimate

7.18 $$\begin{aligned} \Vert \Lambda \Vert _{l^2 X^{\sigma }}\lesssim \Vert \Lambda _0\Vert _{H^{\sigma }}+\Vert F\Vert _{l^2N^{\sigma }}+(\Vert {\mathcal {G}}\Vert _{Z^{\sigma _d,\sigma +2}}+\Vert {\mathcal {A}}\Vert _{Z^{\delta _d,\sigma +1}})\Vert \lambda \Vert _{l^2X^{s}}.\qquad \end{aligned}$$

#### Proof

For $\Lambda$ solving (7.17), we see that $\Lambda _k$ solves

$$\begin{aligned} \left\{ \begin{aligned}&i\partial _t \Lambda _k+\partial _{\alpha }(g^{\alpha {\beta }}_{<k-4}\partial _{{\beta }}\Lambda _k)+2iA^{\alpha }_{<k-4}\partial _{\alpha }\Lambda _k=F_k+G_k+H_k,\\&\Lambda _k(0)=\Lambda _{0k}, \end{aligned}\right. \end{aligned}$$

where

$$\begin{aligned} G_k=-S_k(\nabla ({\mathcal {G}}\nabla \lambda )-2i{\mathcal {A}}^{\alpha }\partial _{\alpha } \lambda ), \end{aligned}$$

$$\begin{aligned} H_k&=-S_k\partial _{\alpha }(g^{\alpha {\beta }}_{\geqq k-4}\partial _{{\beta }}\Lambda )-\partial _{\alpha }[S_k,g^{\alpha {\beta }}_{< k-4}]\partial _{{\beta }}\Lambda -2i[S_k,A^{\alpha }_{<k-4}]\partial _{\alpha }\Lambda \\&\quad -2iS_k(A^{\alpha }_{\geqq k-4}\partial _{\alpha }\Lambda ). \end{aligned}$$

The proof of (7.18) is similar to that of (7.16). Here it suffices to prove

$$\begin{aligned} \sum _{k}\Vert G_k\Vert _{l^2N^{\sigma }}^2\lesssim \Vert {\mathcal {G}}\Vert _{Z^{\sigma _d,\sigma +2}}^2\Vert \lambda \Vert _{l^2X^{s}}^2+\Vert {\mathcal {A}}\Vert _{ Z^{\delta _d,\sigma +1}}^2\Vert \lambda \Vert _{l^2X^{s}}^2. \end{aligned}$$

Indeed, the bound for the terms in $G_k$ follows from (6.9), (6.4), (6.10) and (6.14). This completes the proof of the Lemma. $\square$

## Well-Posedness in the Good Gauge

In this section we use the parabolic results in Section 5, the multilinear estimates in Section 6 and the linear local energy decay bounds in Section 7 in order to prove the good gauge formulation of our main result, namely Theorem 2.5.

### The Iteration Scheme: Uniform Bounds

Here we seek to construct solutions to (2.28) iteratively, based on the scheme

8.1 $$\begin{aligned} \left\{ \begin{aligned}&i\partial _t\lambda ^{(n+1)}+\partial _{\alpha }(g^{(n)\alpha {\beta }}\partial _{{\beta }}\lambda ^{(n+1)})+2iA^{(n)\alpha }\partial _{\alpha }\lambda ^{(n+1)}=F^{(n)},\\&\lambda ^{(n+1)}(0)=\lambda _0, \end{aligned}\right. \end{aligned}$$

with the trivial initialization

$$\begin{aligned} \lambda ^{(0)}=0, \end{aligned}$$

where the nonlinearities $F^{(n)}$ are the following *F* with $(\lambda ,h,A)=(\lambda ^{(n)},h^{(n)},A^{(n)})$

8.2 $$\begin{aligned} F= & {} \partial _\mu (g^{\mu \nu }\partial _\nu \lambda _{\alpha {\beta }})-\nabla ^\sigma \nabla _\sigma \lambda _{\alpha {\beta }}+iV^\sigma \nabla _\sigma \lambda _{\alpha {\beta }}-i\nabla _\sigma A^\sigma \lambda _{\alpha {\beta }}+i\lambda ^{\gamma }_{\alpha }\nabla _{{\beta }} V_{\gamma } +i\lambda _{\beta }^\gamma \nabla _\alpha V_\gamma \nonumber \\{} & {} +(B+A_\sigma A^\sigma -V_\sigma A^\sigma )\lambda _{\alpha {\beta }} +\psi \mathop {\textrm{Re}}\nolimits (\lambda _{\alpha \delta }{\bar{\lambda }}^\delta _{\beta }) -R_{\alpha \sigma {\beta }\delta }\lambda ^{\sigma \delta } -\lambda _{\alpha \mu }{\bar{\lambda }}^\mu _\sigma \lambda ^\sigma _{\beta }, \end{aligned}$$

and ${{\mathcal {S}}}^{(n)}=(h^{(n)},A^{(n)})$ are the solutions of parabolic system (2.29) with $\lambda =\lambda ^{(n)}$ and initial data

8.3 $$\begin{aligned} h^{(n)}(0,x)=h_0(x), \quad A^{(n)}(0,x)=A_0(x). \end{aligned}$$

We assume that $(\lambda _0,h_0)$ is small in $H^s\times {\textbf{Y}}^{s+2}$. Due to the above trivial initialization for $\lambda ^{(0)}$, we also inductively assume that

8.4 $$\begin{aligned} \Vert \lambda ^{(n)}\Vert _{l^2 X^s}\leqq C\Vert \lambda _0\Vert _{H^s}, \end{aligned}$$

where *C* is a large constant.

Applying the parabolic estimates (5.1) to (2.29) with $\lambda =\lambda ^{(n)}$ and initial data (8.3) at each step, we obtain

8.5 $$\begin{aligned} \Vert {{\mathcal {S}}}^{(n)}\Vert _{{{\varvec{ {\mathcal {E}}}}}^{s}}\lesssim \Vert (h_0,A_0)\Vert _{{{\varvec{ {\mathcal {E}}}}}_0^s} + \Vert \lambda ^{(n)}\Vert _{l^2Z^s}\lesssim \Vert (h_0,A_0)\Vert _{{{\varvec{ {\mathcal {E}}}}}_0^s}+\Vert \lambda _0\Vert _{H^s}\lesssim \epsilon _0. \end{aligned}$$

In order to estimate $\lambda ^{(n+1)}$, we bound the nonlinear terms in $F^{(n)}$ first. In the computations we would omit the superscript (*n*). More precisely, for the first three terms in (8.2), by covariant derivatives (2.2) and $V^\gamma =g^{\alpha {\beta }}\Gamma ^\gamma _{\alpha {\beta }}$ we have the form

$$\begin{aligned} \partial _\mu (g^{\mu \nu }\partial _\nu \lambda _{\alpha {\beta }})-\nabla ^\sigma \nabla _\sigma \lambda _{\alpha {\beta }}+iV^\sigma \nabla _\sigma \lambda _{\alpha {\beta }}\approx \nabla h \nabla \lambda +\nabla h\nabla h \lambda . \end{aligned}$$

Then the first term $\nabla h\nabla \lambda$ is estimated using (6.1) and (6.9), the second term $\nabla h\nabla h\lambda$ is estimated using (6.7) with its $A=\nabla h$. We obtain

$$\begin{aligned} \Vert \nabla h \nabla \lambda +\nabla h\nabla h \lambda \Vert _{l^2 N^s}\lesssim \Vert h\Vert _{{\textbf{Y}}^{s+2}}\Vert \lambda \Vert _{l^2 X^s}+\Vert h\Vert _{Z^{s+1}}^2\Vert \lambda \Vert _{Z^s}. \end{aligned}$$

For the fourth to seventh terms in (8.2), we have the expression

$$\begin{aligned}&-i\nabla _\sigma A^\sigma \lambda _{\alpha {\beta }}+i\lambda ^{\gamma }_{\alpha }\nabla _{{\beta }} V_{\gamma } +i\lambda _{\beta }^\gamma \nabla _\alpha V_\gamma +(B+A_\sigma A^\sigma -V_\sigma A^\sigma )\lambda _{\alpha {\beta }}\\&\quad \approx (\nabla ^2 h+\nabla A)\lambda +(\nabla h+A)^2 \lambda . \end{aligned}$$

Then these two terms are estimated using (6.6) and (6.7) respectively. We obtain

$$\begin{aligned} \Vert (\nabla ^2 h+\nabla A)\lambda +(\nabla h+A)^2 \lambda \Vert _{l^2N^s}\lesssim (1+\Vert {{\mathcal {S}}}\Vert _{{{\mathcal {E}}}^s})\Vert {{\mathcal {S}}}\Vert _{{{\mathcal {E}}}^s}\Vert \lambda \Vert _{Z^s}. \end{aligned}$$

For the last three terms in (8.2), by (2.8) we have

$$\begin{aligned} \psi \mathop {\textrm{Re}}\nolimits (\lambda _{\alpha \delta }{\bar{\lambda }}^\delta _{\beta }) -R_{\alpha \sigma {\beta }\delta }\lambda ^{\sigma \delta } -\lambda _{\alpha \mu }{\bar{\lambda }}^\mu _\sigma \lambda ^\sigma _{\beta }\approx \lambda ^3. \end{aligned}$$

Using (6.13) we obtain

$$\begin{aligned} \Vert \lambda ^3\Vert _{l^2N^s}\lesssim \Vert \lambda \Vert _{Z^s}^3. \end{aligned}$$

Hence, by the above estimates, (8.5) and (8.4) we bound the $F^{(n)}$ by

$$\begin{aligned} \Vert F^{(n)}\Vert _{l^2N^s}\lesssim \ (1+\Vert {{\mathcal {S}}}^{(n)}\Vert _{{{\varvec{ {\mathcal {E}}}}}^s})\Vert {{\mathcal {S}}}^{(n)}\Vert _{{{\varvec{ {\mathcal {E}}}}}^s}\Vert \lambda ^{(n)}\Vert _{l^2X^s} \lesssim \ \epsilon _0 \Vert \lambda _0\Vert _{H^s}. \end{aligned}$$

Now applying at each step the local energy bound (7.15) with $\sigma =s$ we obtain the estimate

8.6 $$\begin{aligned} \Vert \lambda ^{(n+1)}\Vert _{l^2 X^s}\lesssim \Vert \lambda _0\Vert _{H^s}+\Vert F^{(n)}\Vert _{l^2N^s} \lesssim \Vert \lambda _0\Vert _{H^s}+C\epsilon _0 \Vert \lambda _0\Vert _{H^s} \leqq C\Vert \lambda _0\Vert _{H^s}, \end{aligned}$$

which closes our induction.

### The Iteration Scheme: Weak Convergence

Here we prove that our iteration scheme converges in the weaker $H^{s-2}$ topology. We denote the differences by

$$\begin{aligned} \Lambda ^{(n+1)}= & {} \lambda ^{(n+1)}-\lambda ^{(n)},\\ {{\varvec{\delta }}} {{\mathcal {S}}}^{(n+1)}= & {} ({{\mathcal {G}}}^{(n+1)},{{\mathcal {A}}}^{(n+1)},{{\mathcal {B}}}^{(n+1)})={{\mathcal {S}}}^{(n+1)}-{{\mathcal {S}}}^{(n)} \end{aligned}$$

Then from (8.1) we obtain the system

$$\begin{aligned} \left\{ \begin{aligned}&i\partial _t \Lambda ^{(n+1)}+\partial _\alpha ( g^{(n)\alpha {\beta }}\partial _{{\beta }}\Lambda ^{(n+1)})+2iA^{(n)\alpha }\partial _{\alpha }\Lambda ^{(n+1)}=F^{(n)}-F^{(n-1)}+G^{(n)},\\&\Lambda ^{(n+1)}(0,x)=0, \end{aligned}\right. \end{aligned}$$

where the nonlinearities $G^{(n)}$ have the form

$$\begin{aligned} G^{(n)}&=-\partial _\alpha ({{\mathcal {G}}}^{(n)}\partial _{{\beta }}\lambda ^{(n)})-2i{{\mathcal {A}}}^{(n)\alpha }\partial _{\alpha }\lambda ^{(n)}, \end{aligned}$$

By (5.4) we obtain

8.7 $$\begin{aligned} \Vert {{\varvec{\delta }}} {{\mathcal {S}}}^{(n)}\Vert _{{{\mathcal {E}}}^{s-2}}\lesssim \Vert \Lambda ^{(n)}\Vert _{l^2 X^{s-2}}. \end{aligned}$$

Applying (7.18) with $\sigma =s-2$ for the $\Lambda ^{(n+1)}$ equation we have

$$\begin{aligned} \Vert \Lambda ^{(n+1)}\Vert _{l^2X^{s-2}}\lesssim \Vert F^{(n)}-F^{(n-1)}\Vert _{l^2N^{s-2}}+(\Vert {{\mathcal {G}}}^{(n)}\Vert _{Z^{\sigma _d,s}}+\Vert {{\mathcal {A}}}^{(n)}\Vert _{ Z^{\delta _d,s-1}})\Vert \lambda ^{(n)}\Vert _{l^2X^{s}}. \end{aligned}$$

For the nonlinear terms $F^{(n)}-F^{(n-1)}$, using (6.3), (6.9), (6.12), (6.11) and (6.13) we have

$$\begin{aligned} \Vert F^{(n)}-F^{(n-1)}\Vert _{l^2N^{s-2}}&\lesssim \ (1+\Vert ({{\mathcal {S}}}^{(n)},{{\mathcal {S}}}^{(n-1)})\Vert _{{{\mathcal {E}}}^s})^N\big (\Vert {{\varvec{\delta }}} {{\mathcal {S}}}^{(n)}\Vert _{{{\mathcal {E}}}^{s-2}}\Vert (\lambda ^{(n)},\lambda ^{(n-1)})\Vert _{l^2X^s}\\&\quad +\Vert ({{\mathcal {S}}}^{(n)},{{\mathcal {S}}}^{(n-1)})\Vert _{{{\mathcal {E}}}^s}\Vert \Lambda ^{(n)}\Vert _{l^2X^{s-2}}\big ). \end{aligned}$$

Then by (8.7) and the uniform bounds (8.5), (8.6) we bound the right hand side above by

$$\begin{aligned} \Vert \Lambda ^{(n+1)}\Vert _{l^2X^{s-2}}&\lesssim \ (1+\Vert {{\mathcal {S}}}_0\Vert _{{{\varvec{ {\mathcal {E}}}}}_0^s}+\Vert \lambda _0\Vert _{H^s})^N \\&\quad \cdot \big [ \Vert \Lambda ^{(n)}\Vert _{l^2X^{s-2}} \Vert \lambda _0\Vert _{H^s}+(\Vert {{\mathcal {S}}}_0\Vert _{{{\varvec{ {\mathcal {E}}}}}_0^s}+\Vert \lambda _0\Vert _{H^s})\Vert \Lambda ^{(n)}\Vert _{l^2 X^{s-2}} \big ] \\&\ll \Vert \Lambda ^{(n)}\Vert _{l^2X^{s-2}}. \end{aligned}$$

This implies that our iterations $\lambda ^{(n)}$ converge in $l^2X^{s-2}$ to some function $\lambda$. Furthermore, by the uniform bound (8.6) it follows that

8.8 $$\begin{aligned} \Vert \lambda \Vert _{l^2X^s}\lesssim \Vert \lambda _0\Vert _{H^s}. \end{aligned}$$

Interpolating, it follows that $\lambda ^{(n)}$ converges to $\lambda$ in $l^2X^{s-\epsilon }$ for all $\epsilon > 0$. This allows us to conclude that the auxiliary functions ${{\mathcal {S}}}^{(n)}$ associated to $\lambda ^{(n)}$ converge to the functions ${{\mathcal {S}}}$ associated to $\lambda$, and also to pass to the limit and conclude that $\lambda$ solves the (SMCF) equation (2.28). Moreover, we have the bound for ${{\mathcal {S}}}$

8.9 $$\begin{aligned} \Vert {{\mathcal {S}}}\Vert _{{{\varvec{ {\mathcal {E}}}}}^{s}}\lesssim \Vert {{\mathcal {S}}}_0\Vert _{{\textbf{Y}}_0^{s+2}}+\Vert \lambda _0\Vert _{H^s}. \end{aligned}$$

Thus we have established the existence part of our main theorem.

### Uniqueness via Weak Lipschitz Dependence

Consider the difference of two solutions

$$\begin{aligned} (\Lambda ,{{\varvec{\delta }}} {{\mathcal {S}}})=(\lambda ^{(1)}-\lambda ^{(2)},{{\mathcal {S}}}^{(1)}-{{\mathcal {S}}}^{(2)}). \end{aligned}$$

The $\Lambda$ solves an equation of this form

$$\begin{aligned} \left\{ \begin{aligned}&i\partial _t \Lambda +\partial _{\alpha }(g^{(1)\alpha {\beta }}\partial _{{\beta }}\Lambda )+2iA^{(1)\alpha }\partial _{\alpha }\Lambda =F^{(1)}-F^{(2)}+G,\\&\Lambda (0,x)=\lambda ^{(1)}_0(x)-\lambda ^{(2)}_0(x), \end{aligned}\right. \end{aligned}$$

where the nonlinearity *G* is

$$\begin{aligned} G&=-\partial _{\alpha }({{\mathcal {G}}}\partial _{{\beta }}\lambda ^{(2)})-2i{{\mathcal {A}}}^{\alpha }\partial _{\alpha }\lambda ^{(2)}. \end{aligned}$$

By (5.4) we have

$$\begin{aligned} \Vert {{\varvec{\delta }}} {{\mathcal {S}}}\Vert _{{{\mathcal {E}}}^{s-2}}\lesssim \Vert {{\varvec{\delta }}} {{\mathcal {S}}}_0\Vert _{{{\mathcal {H}}}^{s-2}}+ \Vert \Lambda \Vert _{l^2 X^{s-2}}. \end{aligned}$$

Applying (7.18) with $\sigma =s-2$ to the $\Lambda$ equation, we obtain the estimate

$$\begin{aligned} \Vert \Lambda \Vert _{l^2 X^{s-2}}&\lesssim \ \Vert \Lambda _0\Vert _{H^{s-2}}+\Vert F^{(1)}-F^{(2)}\Vert _{l^2N^{s-2}}+(\Vert {{\mathcal {G}}}\Vert _{Z^{\sigma _d,s}}+\Vert {{\mathcal {A}}}\Vert _{Z^{\delta _d,s-1}})\Vert \lambda ^{(2)}\Vert _{l^2X^{s}}\\&\lesssim \ \Vert \Lambda _0\Vert _{H^{s-2}}+C\Vert (\lambda ^{(1)}_0,\lambda ^{(2)}_0)\Vert _{H^{s}}\Vert (\Lambda ,{{\varvec{\delta }}} {{\mathcal {S}}})\Vert _{l^2 X^{s-2}\times {{\mathcal {E}}}^{s-2}}. \end{aligned}$$

Then, by the above bound for ${{\varvec{\delta }}} {{\mathcal {S}}}$, we further have

$$\begin{aligned} \Vert \Lambda \Vert _{l^2X^{s-2}}\lesssim \Vert \Lambda _0\Vert _{H^{s-2}}+C\Vert (\lambda ^{(1)}_0,\lambda ^{(2)}_0)\Vert _{H^{s}}(\Vert {{\varvec{\delta }}} {{\mathcal {S}}}_0\Vert _{{{\mathcal {H}}}^{s-2}}+ \Vert \Lambda \Vert _{l^2 X^{s-2}}) \end{aligned}$$

Since the initial data $\lambda ^{(1)}_0$ and $\lambda ^{(2)}_0$ are sufficiently small, we obtain

8.10 $$\begin{aligned} \Vert \Lambda \Vert _{l^2X^{s-2}}\lesssim \Vert \Lambda _0\Vert _{H^{s-2}}+\Vert {{\varvec{\delta }}} {{\mathcal {S}}}_0\Vert _{{{\mathcal {H}}}^{s-2}}. \end{aligned}$$

This gives the weak Lipschitz dependence, as well as the uniqueness of solutions for (2.28).

### Frequency Envelope Bounds

Here we prove a stronger frequency envelope version of estimate (8.8).

#### Proposition 8.1

Let $\lambda \in l^2X^s$, ${{\mathcal {S}}}\in {{\varvec{ {\mathcal {E}}}}}^{s}$ be small data solution to (2.28)–(2.29), which satisfies (8.8) and (8.9). Let $\{p_{0k}\}$, $\{s_{0k}\}$ be admissible frequency envelopes for the initial data $\lambda _0\in H^s$ and ${{\mathcal {S}}}_0\in {{\varvec{ {\mathcal {E}}}}}_0^{s}$. Then $\{p_{0k}+s_{0k}\}$ is also frequency envelope for $(\lambda ,{{\mathcal {S}}})$ in $l^2X^s\times {{\varvec{ {\mathcal {E}}}}}^{s}$.

#### Proof

Let $p_k$ and $s_k$ be the admissible frequency envelopes for solution $(\lambda ,{{\mathcal {S}}})\in l^2X^s\times {{\varvec{ {\mathcal {E}}}}}^s$. Applying $S_k$ to the modified Schrödinger equation in (2.28), we obtain the paradifferential equation

$$\begin{aligned} \left\{ \begin{aligned}&i\partial _t\lambda _k+\partial _{\alpha }(g_{<k-4}^{\alpha {\beta }}\partial _{{\beta }}\lambda _k)+2iA_{<k-4}^{\alpha }\partial _{\alpha }\lambda _k=F_k+J_k,\\&\lambda (0,x)=\lambda _0(x), \end{aligned}\right. \end{aligned}$$

where

$$\begin{aligned} J_k&=-S_k\partial _{\alpha }(g^{\alpha {\beta }}_{\geqq k-4}\partial _{{\beta }}\lambda )-[S_k,\partial _{\alpha }g^{\alpha {\beta }}_{< k-4}\partial _{{\beta }}]\lambda \\&\quad -2i[S_k,A^{\alpha }_{<k-4}]\partial _{\alpha }\lambda -2iS_k[ A^{\alpha }_{\geqq k-4}\partial _{\alpha }\lambda _k], \end{aligned}$$

and ${{\mathcal {S}}}=(h,A)$ is the solution to the parabolic system (2.29). We estimate $\lambda _k=S_k\lambda$ using Proposition 7.4,

$$\begin{aligned} \Vert \lambda _k\Vert _{l^2X^s}&\lesssim \ p_{0k}+\Vert F_k \Vert _{l^2N^s}+\Vert J_k \Vert _{l^2N^s}. \end{aligned}$$

By Proposition 6.2, Lemma 6.1 and Lemma 6.3 we bound the nonlinear terms by

$$\begin{aligned} \begin{aligned} \Vert F_k \Vert _{l^2N^s}+\Vert J_k\Vert _{l^2N^s}&\lesssim (1+\Vert {{\mathcal {S}}}\Vert _{{{\varvec{ {\mathcal {E}}}}}^s}+\Vert \lambda \Vert _{l^2X^s})^N(\Vert {{\mathcal {S}}}\Vert _{{{\varvec{ {\mathcal {E}}}}}^s}p_k+s_k\Vert \lambda \Vert _{l^2X^s}). \end{aligned} \end{aligned}$$

Then by (8.9), (8.8), (5.10) and the smallness of initial data we obtain

$$\begin{aligned} \Vert \lambda _k\Vert _{l^2X^s}\lesssim p_{0k}+\epsilon p_k+\epsilon (s_{0k}+p_k)\lesssim p_{0k}+s_{0k}+\epsilon p_k. \end{aligned}$$

For metric $g=I_d+h$, by (5.2) we also have

$$\begin{aligned} \Vert {{\mathcal {S}}}_k\Vert _{{{\varvec{ {\mathcal {E}}}}}^{s}}\lesssim s_{0k}+\epsilon p_k. \end{aligned}$$

From the definition of frequency envelope (3.3), these two bounds imply

$$\begin{aligned} p_k+s_k\lesssim p_{0k}+s_{0k}. \end{aligned}$$

and conclude the proof. $\square$

### Continuous Dependence on the Initial Data

Here we show that the map $(\lambda _0,{{\mathcal {S}}}_0)\rightarrow (\lambda ,{{\mathcal {S}}})$ is continuous from $H^s\times {{\varvec{ {\mathcal {E}}}}}_0^{s}$ into $l^2X^s\times {{\varvec{ {\mathcal {E}}}}}^s$. By (5.3), it suffices to prove $(\lambda _0,{{\mathcal {S}}}_0)\rightarrow \lambda$ is continuous from $H^s\times {{\varvec{ {\mathcal {E}}}}}_0^{s}$ to $l^2 X^s$.

Suppose that $(\lambda _0^{(n)},{{\mathcal {S}}}_0^{(n)})\rightarrow (\lambda _0,{{\mathcal {S}}}_0)$ in $H^s\times {{\varvec{ {\mathcal {E}}}}}_0^{s}$. Denote by $(p_{0k}^{(n)},s_{0k}^{(n)})$, respectively $(p_{0k},s_{0k})$ the frequency envelopes associated to $(\lambda _0^{(n)},{{\mathcal {S}}}_0^{(n)})$, respectively $(\lambda _0,{{\mathcal {S}}}_0)$, given by (3.3). If $(\lambda _0^{(n)},{{\mathcal {S}}}_0^{(n)})\rightarrow (\lambda _0,{{\mathcal {S}}}_0)$ in $H^s\times {{\varvec{ {\mathcal {E}}}}}_0^{s}$ then $(p_{0k}^{(n)},s_{0k}^{(n)})\rightarrow (p_{0k},s_{0k})$ in $l^2$. Then for each $\epsilon >0$ we can find some $N_{\epsilon }$ so that

$$\begin{aligned} \Vert p_{0,>N_{\epsilon }}^{(n)}\Vert _{l^2}+\Vert s_{0,>N_{\epsilon }}^{(n)}\Vert _{l^2}\leqq \epsilon ,\ \text {for all }n. \end{aligned}$$

By Proposition 8.1 we obtain that

8.11 $$\begin{aligned} \Vert \lambda _{>N_{\epsilon }}^{(n)}\Vert _{l^2X^s}\leqq \epsilon ,\ \text {for all }n. \end{aligned}$$

To compare $\lambda ^{(n)}$ with $\lambda$ we use (8.10) for low frequencies and (8.11) for the high frequencies,

$$\begin{aligned} \Vert \lambda ^{(n)}-\lambda \Vert _{l^2X^s}&\lesssim \ \Vert S_{<N_{\epsilon }}(\lambda ^{(n)}-\lambda )\Vert _{l^2X^s}+\Vert S_{>N_{\epsilon }}\lambda ^{(n)}\Vert _{l^2X^s}+\Vert S_{>N_{\epsilon }}\lambda \Vert _{l^2X^s}\\&\lesssim \ 2^{2N_{\epsilon }}\Vert S_{<N_{\epsilon }}(\lambda ^{(n)}-\lambda )\Vert _{l^2X^{s-2}}+2\epsilon \\&\lesssim \ 2^{2N_{\epsilon }}(\Vert S_{<N_{\epsilon }}(\lambda ^{(n)}_0-\lambda _0)\Vert _{H^{s-2}}+\Vert S_{<N_\epsilon }({{\mathcal {S}}}^{(n)}_0-{{\mathcal {S}}}_0)\Vert _{{{\mathcal {H}}}^{s-2}})+2\epsilon . \end{aligned}$$

Letting $n\rightarrow \infty$ we obtain

$$\begin{aligned} \limsup _{n\rightarrow \infty }\Vert \lambda ^{(n)}-\lambda \Vert _{l^2X^s}\lesssim \epsilon . \end{aligned}$$

Letting $\epsilon \rightarrow 0$ we obtain

$$\begin{aligned} \lim _{n\rightarrow 0}\Vert \lambda ^{(n)}-\lambda \Vert _{l^2X^s}=0, \end{aligned}$$

which completes the desired result.

### Higher Regularity

Here we prove that the solution $(\lambda ,{{\mathcal {S}}})$ satisfies the bound

8.12 $$\begin{aligned} \Vert (\lambda ,{{\mathcal {S}}})\Vert _{l^2X^{\sigma }\times {{\varvec{ {\mathcal {E}}}}}^{\sigma }}\lesssim \Vert \lambda _0\Vert _{H^{\sigma }}+\Vert {{\mathcal {S}}}_0\Vert _{{{\varvec{ {\mathcal {E}}}}}_0^{\sigma }},\quad \sigma \geqq s, \end{aligned}$$

whenever the right hand side is finite.

The proof of (8.12) is similar to that in [9, Section 7.6]. Here we simply repeat this process. Differentiating the original Schrödinger equation (2.28), and then using Proposition 7.4, Lemma 6.1 and Proposition 6.2 we easily obtain

$$\begin{aligned} \Vert \nabla \lambda \Vert _{l^2 X^s} \lesssim \Vert \nabla \lambda _0\Vert _{H^s}+\Vert (\nabla \lambda ,\nabla {{\mathcal {S}}})\Vert _{l^2X^s\times {{\varvec{ {\mathcal {E}}}}}^s}\Vert (\lambda ,{{\mathcal {S}}})\Vert _{l^2X^s\times {{\varvec{ {\mathcal {E}}}}}^s}(1+\Vert (\lambda ,{{\mathcal {S}}})\Vert _{l^2X^s\times {{\varvec{ {\mathcal {E}}}}}^s})^N. \end{aligned}$$

For the parabolic equations, by (5.3) we obtain

$$\begin{aligned} \Vert \nabla {{\mathcal {S}}}\Vert _{{{\varvec{ {\mathcal {E}}}}}^s}\lesssim \Vert \nabla {{\mathcal {S}}}_0\Vert _{{{\varvec{ {\mathcal {E}}}}}_0^{s}} + \Vert \lambda \Vert _{l^2X^s} \Vert \nabla \lambda \Vert _{l^2X^s}. \end{aligned}$$

Hence, by (8.8) and (8.9), these imply (8.12) with $\sigma =s+1$. Inductively, we can further obtain (8.12) for any $\sigma \geqq s$.

### The Compatibilities Conditions

As part of our derivation of the (SMCF) equations (2.28) for the second fundamental form $\lambda$ in the good gauge, coupled with the parabolic system (2.29), we have seen that the compatibility conditions are described by the equations (2.9), (2.8), (2.10), (2.12), (2.20) and (2.17). However, our proof of the well-posedness result for the Schrödinger evolution (2.28) does not apriori guarantee that these constraints hold. Here we rectify this omission:

#### Lemma 8.2

(Constraint conditions) Assume that $\lambda \in C[0,T;H^s]$ solves the SMCF equation (2.28) coupled with the parabolic system (2.29). Then the relations (2.9), (2.8), (2.10), (2.12), (2.20) and (2.17) hold.

#### Proof

To shorten the notations, we define

$$\begin{aligned} T^1_{\alpha {\beta }}&={\text {Ric}}_{\alpha {\beta }}-{\widetilde{\text {Ric}}}_{\alpha {\beta }},\ \quad \qquad {\widetilde{\text {Ric}}}_{\alpha {\beta }}:=\mathop {\textrm{Re}}\nolimits (\lambda _{\alpha {\beta }}{\bar{\psi }}-\lambda _{\alpha \sigma }{\bar{\lambda }}^\sigma _{\ {\beta }}),\\ T^2_{\sigma \gamma \alpha {\beta }}&=R_{\sigma \gamma \alpha {\beta }}-{{\tilde{R}}}_{\sigma \gamma \alpha {\beta }},\qquad {{\tilde{R}}}_{\sigma \gamma \alpha {\beta }}:= \mathop {\textrm{Re}}\nolimits (\lambda _{\gamma {\beta }}{\bar{\lambda }}_{\sigma \alpha }-\lambda _{\gamma \alpha }{\bar{\lambda }}_{\sigma {\beta }}), \\ T^3_{\alpha {\beta },\gamma }&=\nabla ^A_\alpha \lambda _{{\beta }\gamma }-\nabla ^A_{\beta }\lambda _{\alpha \gamma },\\ T^4_{\alpha {\beta }}&={\textbf{F}}_{\alpha {\beta }}-\tilde{{\textbf{F}}}_{\alpha {\beta }},\qquad {\textbf{F}}_{\alpha {\beta }}:=\nabla _\alpha A_{\beta }-\nabla _{\beta }A_\alpha ,\ \tilde{{\textbf{F}}}_{\alpha {\beta }}:=\mathop {\textrm{Im}}\nolimits (\lambda _\alpha ^\gamma {\bar{\lambda }}_{\gamma {\beta }}),\\ T^5_{\alpha }&= {\textbf{F}}_{0\alpha } - \tilde{{\textbf{F}}}_{0\alpha },\ \qquad {\textbf{F}}_{0\alpha }:=\partial _t A_\alpha -\nabla _\alpha B,\ \tilde{{\textbf{F}}}_{0\alpha }:= \mathop {\textrm{Re}}\nolimits (\lambda _{\alpha }^{\gamma }{\bar{\partial }}^A_{\gamma }{\bar{\psi }})+\mathop {\textrm{Im}}\nolimits (\lambda ^\gamma _\alpha {\bar{\lambda }}_{\gamma \sigma })V^\sigma . \end{aligned}$$

Here $T^3$ and $T^4$ are antisymmetric, $T^1$ is symmetric and $T^2$ inherits all the linear symmetries of the curvature tensor.

Our goal is to show that all these functions vanish, knowing that they vanish at the initial time. We will prove this by showing that they solve a coupled linear homogeneous evolution system of the form

$$\begin{aligned} \left\{ \begin{aligned}&(\partial _t-\Delta _g)T^{1,{\beta }}_{\alpha }=\lambda ^2 T^4+T^1\nabla V+V\nabla T^1+T^3\nabla \lambda +\lambda \nabla T^3,\\&\nabla _\delta T^2_{\sigma \gamma \alpha {\beta }} + \nabla _{\sigma } T^2_{\gamma \delta \alpha {\beta }} + \nabla _{\gamma } T^2_{\delta \sigma \alpha {\beta }} = T^1\lambda ,\\&\nabla ^{\sigma }T^2_{\sigma \gamma \alpha {\beta }} = \nabla _{\alpha } T^1_{\gamma {\beta }} - \nabla _{{\beta }} T^1_{\gamma \alpha } + T^1\lambda ,\\&\begin{aligned} (i\partial ^B_t -\Delta ^A_g)T^3_{\alpha {\beta },\gamma }=&\ \lambda T^5+T^3(\nabla V+\lambda ^2+R)+(\nabla ^A\lambda +\lambda V)(T^1+T^2+T^4)\\&\ +\lambda \nabla (T^2+T^4)+V\nabla T^3 \end{aligned}\\&(\partial _t-\Delta _g)T^4_{\alpha {\beta }}={\text {Ric}}T^4+\nabla ^A\lambda T^3+V\lambda T^3,\\&T^5_\alpha =\nabla ^\sigma T^4_{\sigma \alpha }+T^1_{\alpha \delta }A^\delta . \end{aligned}\right. \end{aligned}$$

Then standard energy estimates show that zero is the only solution for this system.

The formulas for $T^5$ are obtained directly by the equations for *A* (2.21) and heat gauge $B=\nabla ^\alpha A_\alpha$. It remains to derive the system for $(T^1,\cdots , T^4)$.

#### The equation for $T^1$

 This has the form

$$\begin{aligned} (\partial _t-\Delta _g)T^{1,{\beta }}_{\alpha }=\lambda ^2 T^4+T^1\nabla V+V\nabla T^1+T^3\nabla \lambda +\lambda \nabla T^3. \end{aligned}$$

Using the parabolic equations for *h* we recover the representation of $\partial _t g$ as

8.13 $$\begin{aligned} \partial _t g_{\mu \nu }=2G_{\mu \nu }-2T^1_{\mu \nu },\quad G_{\mu \nu }:=\mathop {\textrm{Im}}\nolimits (\psi {\bar{\lambda }}_{\mu \nu })+\frac{1}{2}\nabla _\mu V_\nu +\frac{1}{2}\nabla _\nu V_\mu , \end{aligned}$$

and obtain

8.14 $$\begin{aligned} \partial _t \Gamma _{\alpha {\beta }}^\gamma =\nabla _\alpha G_{{\beta }}^\gamma +\nabla _{\beta }G_{\alpha }^\gamma -\nabla ^\gamma G_{\alpha {\beta }}-(\nabla _\alpha T_{{\beta }}^{1,\gamma }+\nabla _{\beta }T_{\alpha }^{1,\gamma }-\nabla ^\gamma T^1_{\alpha {\beta }}).\qquad \end{aligned}$$

We then use the two formulas to write

$$\begin{aligned} \partial _t {{\text {Ric}}_\alpha }^{\beta }&= \partial _t g^{\mu \nu } {R_{\mu \alpha \nu }}^{\beta }- g^{\mu \nu }\partial _t {R^{\beta }}_{\mu \nu \alpha }\\&= (-2G^{\mu \nu }+2T^{1,\mu \nu }){R_{\mu \alpha \nu }}^{\beta }+g^{\mu \nu }(\nabla _\alpha \partial _t \Gamma ^{\beta }_{\mu \nu }-\nabla _\nu \partial _t\Gamma ^{\beta }_{\mu \alpha })\\&= 2T^{1,\mu \nu }{R_{\mu \alpha \nu }}^{\beta }-2G^{\mu \nu }{R_{\mu \alpha \nu }}^{\beta }\\&\quad +\nabla _\alpha [2\nabla ^\mu (G_{\mu }^{\beta }-T_{\mu }^{1,{\beta }})-\nabla ^{\beta }(G_{\mu }^\mu -T^{1,\mu }_{\mu })]\\&\quad -\nabla ^\mu [\nabla _\mu (G_{\alpha }^{\beta }-T_{\alpha }^{1,{\beta }})+\nabla _\alpha (G_{\mu }^{\beta }-T_{\mu }^{1,{\beta }})-\nabla ^{\beta }(G_{\mu \alpha }-T^1_{\mu \alpha })]\\&= \nabla ^\mu \nabla _\mu T_{\alpha }^{1,{\beta }}+ 2T^{1,\mu \nu }{R_{\mu \alpha \nu }}^{\beta }+\nabla _\alpha (-2\nabla ^\mu T_{\mu }^{1,{\beta }}+\nabla ^{\beta }T^{1,\mu }_{\mu })\\&\quad +\nabla ^\mu (\nabla _\alpha T_{\mu }^{1,{\beta }}-\nabla ^{\beta }T^1_{\mu \alpha })\\&\quad -2G^{\mu \nu }{R_{\mu \alpha \nu }}^{\beta }+2[\nabla _\alpha ,\nabla ^\mu ] G_{\mu }^{\beta }-\nabla _\alpha \nabla ^{\beta }G_{\mu }^\mu -\nabla ^\mu (\nabla _\mu G_{\alpha }^{\beta }-\nabla _\alpha G_{\mu }^{\beta }-\nabla ^{\beta }G_{\mu \alpha }). \end{aligned}$$

By the relation $\nabla ^\mu T^1_{\mu \nu }=\frac{1}{2}\nabla _\nu T^{1,\mu }_\mu$, the third term in the right hand side vanishes. We can also rewrite the fourth term as

$$\begin{aligned} \nabla ^\mu (\nabla _\alpha T_{\mu }^{1,{\beta }}-\nabla ^{\beta }T^1_{\mu \alpha })&=\ [\nabla ^\mu ,\nabla _\alpha ] T_{\mu }^{1,{\beta }}-[\nabla ^\mu ,\nabla ^{\beta }] T^1_{\mu \alpha }+\nabla _\alpha \nabla ^\mu T_{\mu }^{1,{\beta }}-\nabla ^{\beta }\nabla ^\mu T^1_{\mu \alpha }\\&=\ {R^\mu }_{\alpha \mu \delta }T^{1,\delta {\beta }}+{R^\mu }_{\alpha {\beta }\delta }T^{1,\delta }_\mu -{R^{\mu {\beta }}}_{\mu \delta }T^{1,\delta }_\alpha \\&\quad -{R^{\mu {\beta }}}_{\alpha \delta }T^{1,\delta }_\mu +[\nabla _\alpha ,\nabla ^{\beta }]T^{1,\mu }_\mu , \end{aligned}$$

where the last term vanishes. Commuting we compute the fifth and sixth terms as

$$\begin{aligned} -2G^{\mu \nu }{R_{\mu \alpha \nu }}^{\beta }+2[\nabla _\alpha ,\nabla ^\mu ] G^{{\beta }}_\mu&=-2G^{\mu \nu }{R_{\mu \alpha \nu }}^{\beta }+2 R_{\alpha \mu \mu \nu }G^{{\beta }\nu }+2R_{\alpha \mu {\beta }\nu }G^{\mu \nu }\\&=-2{\text {Ric}}_{\alpha \nu } G^{{\beta }\nu }. \end{aligned}$$

Hence, from the above three formulas and the representation of $G_{\mu \nu }$ (8.13), we rearrange $\partial _t {{\text {Ric}}_\alpha }^{\beta }$ as 
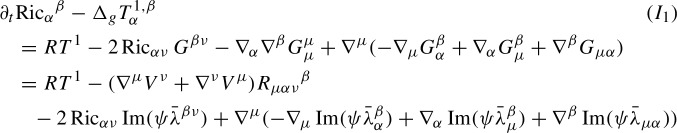

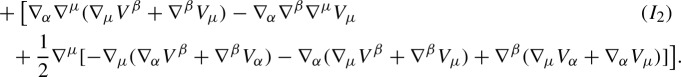
 We write $I_1$ as

$$\begin{aligned} I_1&=\ -2{\text {Ric}}_{\alpha \nu }\mathop {\textrm{Im}}\nolimits (\psi {\bar{\lambda }}^{{\beta }\nu })\\&\quad +\mathop {\textrm{Im}}\nolimits (-\nabla ^{A,\mu }\nabla ^A_{\mu }\psi {\bar{\lambda }}^{\beta }_\alpha -2\nabla ^{A,\mu }\psi \overline{\nabla ^A_\mu \lambda ^{\beta }_\alpha }-\psi \overline{\nabla ^{A,\mu }\nabla ^A_\mu \lambda ^{\beta }_\alpha }\\&\quad +\nabla ^{A,\mu }\nabla ^A_\alpha \psi {\bar{\lambda }}^{\beta }_\mu +\nabla ^{A,\mu }\psi \overline{\nabla ^A_\alpha \lambda ^{\beta }_\mu }+\nabla ^{A}_\alpha \psi \overline{\nabla ^{A,\mu } \lambda ^{\beta }_\mu }+\psi \overline{\nabla ^{A,\mu }\nabla ^A_\alpha \lambda ^{\beta }_\mu }\\&\quad +\nabla ^{A,\mu }\nabla ^{A,{\beta }}\psi {\bar{\lambda }}_{\mu \alpha }+\nabla ^{A,\mu }\psi \overline{\nabla ^{A,{\beta }} \lambda _{\mu \alpha }}+\nabla ^{A,{\beta }}\psi \overline{\nabla ^{A,\mu } \lambda _{\mu \alpha }}+\psi \overline{\nabla ^{A,\mu }\nabla ^{A,{\beta }}\lambda ^{\mu \alpha }})\\&=\ \nabla \psi T^3+\psi \nabla T^3-2{\text {Ric}}_{\alpha \nu }\mathop {\textrm{Im}}\nolimits (\psi {\bar{\lambda }}^{{\beta }\nu })\\&\quad +\mathop {\textrm{Im}}\nolimits (-\nabla ^{A,\mu }\nabla ^A_{\mu }\psi {\bar{\lambda }}^{\beta }_\alpha +\nabla ^{A,\mu }\nabla ^A_\alpha \psi {\bar{\lambda }}^{\beta }_\mu +\nabla ^{A,\mu }\nabla ^{A,{\beta }}\psi {\bar{\lambda }}_{\mu \alpha }+\psi \overline{\nabla ^{A,\mu }\nabla ^{A,{\beta }}\lambda ^{\mu \alpha }}) \end{aligned}$$

Here the $I_1$ term will be cancelled by $J_1,J_2$ later modulo $\{ \psi \nabla T^3,\lambda \lambda T^1,\lambda \lambda T^4\}$. Using commutators we rearrange $I_2$ as

$$\begin{aligned} I_2&=\ [\nabla _\alpha ,\nabla ^\mu ]\nabla _\mu V^{\beta }+\frac{1}{2}\nabla ^\mu [\nabla _\alpha ,\nabla _\mu ]V^{\beta }+\frac{1}{2}\nabla ^\mu [\nabla ^{\beta },\nabla _\mu ]V_\alpha \\&\quad +\nabla _\alpha [\nabla ^\mu ,\nabla ^{\beta }]V_\mu +\frac{1}{2}\nabla ^\mu [\nabla ^{\beta },\nabla _\alpha ]V_\mu . \end{aligned}$$

Then by Riemannian curvature and Bianchi identities we have

$$\begin{aligned} I_2&=\ R_{\alpha \mu \mu \delta }\nabla ^\delta V^{\beta }+R_{\alpha \mu {\beta }\delta }\nabla ^\mu V^\delta \\&\quad +\frac{1}{2}\nabla ^\mu (R_{\alpha \mu {\beta }\delta }V^\delta +R_{{\beta }\mu \alpha \delta }V^\delta +R_{{\beta }\alpha \mu \delta }V^\delta )+\nabla _\alpha (R_{\mu {\beta }\mu \delta }V^\delta )\\&=\ -{\text {Ric}}_{\alpha \delta }\nabla ^\delta V^{\beta }+R_{\alpha \mu {\beta }\delta }\nabla ^\mu V^\delta +\nabla ^\mu (R_{{\beta }\mu \alpha \delta }V^\delta )+\nabla _\alpha ({\text {Ric}}_{{\beta }\delta }V^\delta )\\&=\ -{\text {Ric}}_{\alpha \delta }\nabla ^\delta V^{\beta }+(R_{\alpha \mu {\beta }\delta }+R_{{\beta }\mu \alpha \delta })\nabla ^\mu V^\delta -\nabla _\alpha R_{\delta \mu {\beta }\mu }V^\delta -\nabla _\delta R_{\mu \alpha {\beta }\mu }V^\delta \\&\quad +\nabla _\alpha {\text {Ric}}_{{\beta }\delta }V^\delta +{\text {Ric}}_{{\beta }\delta }\nabla _\alpha V^\delta \\&=\ -{\text {Ric}}_{\alpha \delta }\nabla ^\delta V^{\beta }+R_{\alpha \mu {\beta }\delta }(\nabla ^\mu V^\delta +\nabla ^\delta V_\mu )+\nabla _\delta {\text {Ric}}_{\alpha {\beta }}V^\delta +{\text {Ric}}_{{\beta }\delta }\nabla _\alpha V^\delta , \end{aligned}$$

which gives

$$\begin{aligned} I_2-(\nabla ^\mu V^\nu +\nabla ^\nu V^\mu ){R_{\mu \alpha \nu }}^{\beta }=-{\text {Ric}}_{\alpha \delta }\nabla ^\delta V^{\beta }+V^\delta \nabla _\delta {\text {Ric}}_{\alpha {\beta }} +{\text {Ric}}_{{\beta }\delta }\nabla _\alpha V^\delta . \end{aligned}$$

This term will be cancelled by $J_3$ modulo $\{T^1\nabla V,V\nabla T^1\}$.

Next, we compute the expression for $-\partial _t {{\widetilde{\text {Ric}}}_\alpha }^{\beta }$. From the $\lambda$-equations (2.28) and the formula (8.13) we have the evolution equation for $\lambda ^\sigma _\alpha$

8.15 $$\begin{aligned}{} & {} i\partial _t^B \lambda _\alpha ^\sigma +\frac{1}{2}(\nabla ^A_\alpha \nabla ^{A,\sigma }+\nabla ^{A,\sigma }\nabla ^A_\alpha )\psi +\lambda (T^1+T^2+T^4) +i\lambda ^\gamma _\alpha \left( \frac{3}{2}\mathop {\textrm{Im}}\nolimits (\psi {\bar{\lambda }}^\sigma _\gamma )+\nabla _\gamma V^\sigma \right) \nonumber \\{} & {} \quad -i\lambda ^{\gamma \sigma }\left( \frac{1}{2}\mathop {\textrm{Im}}\nolimits (\psi {\bar{\lambda }}_{\gamma \alpha })+\nabla _\alpha V_\gamma \right) -iV^\gamma \nabla ^A_\gamma \lambda ^\sigma _\alpha =0, \end{aligned}$$

and the evolution equation for the mean curvature $\psi$

$$\begin{aligned} i\partial _t^B \psi +\nabla ^A_\sigma \nabla ^{A,\sigma }\psi +\lambda (T^1+T^2+T^4) +i\lambda ^\gamma _\sigma \mathop {\textrm{Im}}\nolimits (\psi {\bar{\lambda }}^\sigma _\gamma )-iV^\gamma \nabla ^A_\gamma \psi =0. \end{aligned}$$

Then for ${{\widetilde{\text {Ric}}}_\alpha }^{\beta }=\mathop {\textrm{Re}}\nolimits (\lambda _\alpha ^{\beta }{\bar{\psi }}-\lambda _\alpha ^\sigma {\bar{\lambda }}_\sigma ^{\beta })$, by the above two formulas we have

$$\begin{aligned} -\partial _t {{\widetilde{\text {Ric}}}_\alpha }^{\beta }&=\ -\mathop {\textrm{Re}}\nolimits (\partial ^B_t \lambda ^{\beta }_\alpha {\bar{\psi }}+\lambda ^{\beta }_\alpha \overline{\partial ^B_t \psi }-\partial ^B_t\lambda _{\alpha }^\mu {\bar{\lambda }}^{{\beta }}_\mu -\lambda _{\alpha }^\mu \overline{\partial ^B_t\lambda ^{\beta }_\mu })\\&=\ \mathop {\textrm{Im}}\nolimits (-i\partial ^B_t \lambda ^{\beta }_\alpha {\bar{\psi }}-{\bar{\lambda }}^{\beta }_\alpha i\partial ^B_t \psi +i\partial ^B_t\lambda _{\alpha }^\mu {\bar{\lambda }}^{{\beta }}_\mu +{\bar{\lambda }}_{\alpha }^\mu i\partial ^B_t\lambda ^{\beta }_\mu )\\&=\ \lambda ^2(T^1+T^2+T^4)+K_1+K_2+K_3+K_4, \end{aligned}$$

where

$$\begin{aligned} K_1&=\ \mathop {\textrm{Im}}\nolimits \Big [ \Big ( \frac{1}{2}(\nabla ^A_\alpha \nabla ^{A,{\beta }}+\nabla ^{A,{\beta }}\nabla ^A_\alpha )\psi +i\lambda ^\gamma _\alpha \Big (\frac{3}{2}\mathop {\textrm{Im}}\nolimits (\psi {\bar{\lambda }}^{\beta }_\gamma )+\nabla _\gamma V^{\beta }\Big )\\&\quad -i\lambda ^{\gamma {\beta }}\Big (\frac{1}{2}\mathop {\textrm{Im}}\nolimits (\psi {\bar{\lambda }}_{\gamma \alpha })+\nabla _\alpha V_\gamma \Big )-iV^\gamma \nabla ^A_\gamma \lambda ^{\beta }_\alpha \Big ){\bar{\psi }}\Big ],\\ K_2&=\ \mathop {\textrm{Im}}\nolimits \Big [{\bar{\lambda }}^{\beta }_\alpha \Big ( \nabla ^A_\sigma \nabla ^{A,\sigma }\psi +i\lambda ^\gamma _\sigma \mathop {\textrm{Im}}\nolimits (\psi {\bar{\lambda }}^\sigma _\gamma )-iV^\gamma \nabla ^A_\gamma \psi \Big ) \Big ],\\ K_3&=\ -\mathop {\textrm{Im}}\nolimits \Big [ \Big ( \frac{1}{2}(\nabla ^A_\alpha \nabla ^{A,\sigma }+\nabla ^{A,\sigma }\nabla ^A_\alpha )\psi +i\lambda ^\gamma _\alpha \Big (\frac{3}{2}\mathop {\textrm{Im}}\nolimits (\psi {\bar{\lambda }}^\sigma _\gamma )+\nabla _\gamma V^\sigma \Big )\\&\quad -i\lambda ^{\gamma \sigma }\Big (\frac{1}{2}\mathop {\textrm{Im}}\nolimits (\psi {\bar{\lambda }}_{\gamma \alpha })+\nabla _\alpha V_\gamma \Big )-iV^\gamma \nabla ^A_\gamma \lambda ^\sigma _\alpha \Big ){\bar{\lambda }}^{\beta }_\sigma \Big ],\\ K_4&=\ -\mathop {\textrm{Im}}\nolimits \Big [ {\bar{\lambda }}^\sigma _\alpha \Big ( \frac{1}{2}(\nabla ^A_\sigma \nabla ^{A,{\beta }}+\nabla ^{A,{\beta }}\nabla ^A_\sigma )\psi +i\lambda ^\gamma _\sigma \Big (\frac{3}{2}\mathop {\textrm{Im}}\nolimits (\psi {\bar{\lambda }}^{\beta }_\gamma )+\nabla _\gamma V^{\beta }\Big )\\&\quad -i\lambda ^{\gamma {\beta }}\Big (\frac{1}{2}\mathop {\textrm{Im}}\nolimits (\psi {\bar{\lambda }}_{\gamma \sigma })+\nabla _\sigma V_\gamma \Big )-iV^\gamma \nabla ^A_\gamma \lambda ^{\beta }_\sigma \Big )\Big ]. \end{aligned}$$

This can be further rearranged as

$$\begin{aligned} -\partial _t {{\widetilde{\text {Ric}}}_\alpha }^{\beta }=\lambda ^2(T^1+T^2+T^4)+J_1+J_2+J_3, \end{aligned}$$

where $J_1$, $J_2$ and $J_3$ are

$$\begin{aligned} J_1&=\ \mathop {\textrm{Im}}\nolimits \Big [ \frac{1}{2}(\nabla ^A_\alpha \nabla ^{A,{\beta }}\psi +\nabla ^{A,{\beta }}\nabla ^A_\alpha \psi ) {\bar{\psi }}+{\bar{\lambda }}^{\beta }_\alpha \nabla ^A_\sigma \nabla ^{A,\sigma }\psi \\&\quad -\frac{1}{2}(\nabla ^A_\alpha \nabla ^{A,\sigma }\psi +\nabla ^{A,\sigma }\nabla ^A_\alpha \psi ) {\bar{\lambda }}^{\beta }_\sigma -\frac{1}{2}{\bar{\lambda }}^\sigma _\alpha (\nabla ^A_\sigma \nabla ^{A,{\beta }}\psi +\nabla ^{A,{\beta }}\nabla ^A_\sigma \psi ) \Big ], \\ J_2&=\ \frac{3}{2}\mathop {\textrm{Re}}\nolimits (\lambda ^\gamma _\alpha {\bar{\psi }})\mathop {\textrm{Im}}\nolimits (\psi {\bar{\lambda }}^{\beta }_\gamma ) -\frac{1}{2}\mathop {\textrm{Re}}\nolimits (\lambda ^{\gamma {\beta }}{\bar{\psi }})\mathop {\textrm{Im}}\nolimits (\psi {\bar{\lambda }}_{\gamma \alpha }) +\mathop {\textrm{Re}}\nolimits ({\bar{\lambda }}^{\beta }_\alpha \lambda ^\gamma _\sigma ) \mathop {\textrm{Im}}\nolimits (\psi {\bar{\lambda }}^\sigma _\gamma ) \\&\quad -\frac{3}{2}\mathop {\textrm{Re}}\nolimits (\lambda ^\gamma _\alpha {\bar{\lambda }}^{\beta }_\sigma ) \mathop {\textrm{Im}}\nolimits (\psi {\bar{\lambda }}^\sigma _\gamma ) +\frac{1}{2}\mathop {\textrm{Re}}\nolimits (\lambda ^{\gamma \sigma }{\bar{\lambda }}^{\beta }_\sigma )\mathop {\textrm{Im}}\nolimits (\psi {\bar{\lambda }}_{\gamma \alpha }) \\&\quad -\frac{3}{2}\mathop {\textrm{Re}}\nolimits ({\bar{\lambda }}^\sigma _\alpha \lambda ^\gamma _\sigma ) \mathop {\textrm{Im}}\nolimits (\psi {\bar{\lambda }}^{\beta }_\gamma ) +\frac{1}{2}\mathop {\textrm{Re}}\nolimits ({\bar{\lambda }}^\sigma _\alpha \lambda ^{\gamma {\beta }})\mathop {\textrm{Im}}\nolimits (\psi {\bar{\lambda }}_{\gamma \sigma }) ,\\ J_3&= \ \mathop {\textrm{Re}}\nolimits (\lambda ^\gamma _\alpha {\bar{\psi }}) \nabla _\gamma V^{\beta }-\mathop {\textrm{Re}}\nolimits (\lambda ^{\gamma {\beta }}{\bar{\psi }})\nabla _\alpha V_\gamma -V^\gamma \mathop {\textrm{Re}}\nolimits ( \nabla ^A_\gamma \lambda ^{\beta }_\alpha {\bar{\psi }})\\&\quad -V^\gamma \mathop {\textrm{Re}}\nolimits ({\bar{\lambda }}^{\beta }_\alpha \nabla ^A_\gamma \psi ) \\&\quad -\mathop {\textrm{Re}}\nolimits (\lambda ^\gamma _\alpha {\bar{\lambda }}^{\beta }_\sigma ) \nabla _\gamma V^\sigma +\mathop {\textrm{Re}}\nolimits (\lambda ^{\gamma \sigma }{\bar{\lambda }}^{\beta }_\sigma )\nabla _\alpha V_\gamma +V^\gamma \mathop {\textrm{Re}}\nolimits (\nabla ^A_\gamma \lambda ^\sigma _\alpha {\bar{\lambda }}^{\beta }_\sigma ) \\&\quad -\mathop {\textrm{Re}}\nolimits ({\bar{\lambda }}^\sigma _\alpha \lambda ^\gamma _\sigma )\nabla _\gamma V^{\beta }+\mathop {\textrm{Re}}\nolimits ({\bar{\lambda }}^\sigma _\alpha \lambda ^{\gamma {\beta }})\nabla _\sigma V_\gamma +V^\gamma \mathop {\textrm{Re}}\nolimits ({\bar{\lambda }}^\sigma _\alpha \nabla ^A_\gamma \lambda ^{\beta }_\sigma ). \end{aligned}$$

Then $I_1+J_1+J_2$ will vanish modulo $\{\psi \nabla T^3,\lambda ^2 T^1,\lambda ^2 T^4\}$. Precisely, we have

$$\begin{aligned} I_1+J_1&=\ \nabla \psi T^3+\psi \nabla T^3-2{\text {Ric}}_{\alpha \nu }\mathop {\textrm{Im}}\nolimits (\psi {\bar{\lambda }}^{{\beta }\nu })\\&\quad +\mathop {\textrm{Im}}\nolimits \bigg ( \frac{1}{2}[\nabla ^{A,\mu },\nabla ^A_\alpha ]\psi {\bar{\lambda }}^{\beta }_\mu +\frac{1}{2}[\nabla ^{A,\mu },\nabla ^{A,{\beta }}]\psi {\bar{\lambda }}_{\mu \alpha }+\psi \overline{[\nabla ^{A,\mu },\nabla ^{A,{\beta }}]\lambda _{\mu \alpha }}\\&\quad +\psi \overline{\nabla ^{A,{\beta }}T_{\mu \alpha ,}^{3\ \mu }}+\frac{1}{2}\psi \overline{[\nabla ^{A,{\beta }},\nabla ^A_\alpha ]\psi } \bigg )\\&=\ \nabla \psi T^3+\psi \nabla T^3-2{\text {Ric}}_{\alpha \nu }\mathop {\textrm{Im}}\nolimits (\psi {\bar{\lambda }}^{{\beta }\nu }) +\frac{1}{2}{{\textbf{F}}^\mu }_\alpha \mathop {\textrm{Re}}\nolimits (\psi {\bar{\lambda }}^{\beta }_\mu ) -\frac{1}{2}{\textbf{F}}^{\mu {\beta }}\mathop {\textrm{Re}}\nolimits (\psi {\bar{\lambda }}_{\mu \alpha })\\&\quad + {\text {Ric}}^{\mu {\beta }} \mathop {\textrm{Im}}\nolimits (\psi {\bar{\lambda }}_{\mu \alpha })+ R_{\mu {\beta }\alpha \delta }\mathop {\textrm{Im}}\nolimits (\psi {\bar{\lambda }}^{\mu \delta }) -\frac{1}{2}|\psi |^2{{\textbf{F}}^{\beta }}_{\alpha }. \end{aligned}$$

We rewrite $J_2$ as

$$\begin{aligned} J_2&= \ \frac{3}{2}\mathop {\textrm{Im}}\nolimits (\psi {\bar{\lambda }}^{{\beta }\gamma }){\widetilde{\text {Ric}}}_{\alpha \gamma }-\frac{1}{2}\mathop {\textrm{Im}}\nolimits (\psi {\bar{\lambda }}_{\gamma \alpha }){\widetilde{\text {Ric}}}^{\gamma {\beta }}+\mathop {\textrm{Im}}\nolimits (\psi {\bar{\lambda }}^{\sigma \gamma }){{{\tilde{R}}}^{\beta }}_{\ \sigma \alpha \gamma }. \end{aligned}$$

Then we obtain

$$\begin{aligned} I_1+J_1+J_2&= \ \psi \nabla T^3+\lambda ^2 (T^1+T^4) +\frac{1}{2}\mathop {\textrm{Im}}\nolimits (\psi {\bar{\lambda }}_{\gamma \alpha }){\widetilde{\text {Ric}}}^{\gamma {\beta }}-\frac{1}{2}\mathop {\textrm{Im}}\nolimits (\psi {\bar{\lambda }}^{{\beta }\gamma }){\widetilde{\text {Ric}}}_{\alpha \gamma }\\&\quad +\frac{1}{2}{\tilde{{\textbf{F}}}^\mu }_{\ \alpha } \mathop {\textrm{Re}}\nolimits (\psi {\bar{\lambda }}^{\beta }_\mu ) -\frac{1}{2}\tilde{{\textbf{F}}}^{\mu {\beta }}\mathop {\textrm{Re}}\nolimits (\psi {\bar{\lambda }}_{\mu \alpha })-\frac{1}{2}|\psi |^2{\tilde{{\textbf{F}}}^{\beta }}_{\ \alpha }\\&= \ \psi \nabla T^3+\lambda ^2 (T^1+T^4). \end{aligned}$$

We can also show that $I_2-(\nabla ^\mu V^\nu +\nabla ^\nu V^\mu ){R_{\mu \alpha \nu }}^{\beta }+J_3$ vanishes modulo $\{T^1\nabla V,V\nabla T^1\}$. This is because $J_3$ can be written as

$$\begin{aligned} J_3&= \ \mathop {\textrm{Re}}\nolimits (\lambda ^\gamma _\alpha {\bar{\psi }}) \nabla _\gamma V^{\beta }-\mathop {\textrm{Re}}\nolimits (\lambda ^{\gamma {\beta }}{\bar{\psi }})\nabla _\alpha V_\gamma -V^\gamma \mathop {\textrm{Re}}\nolimits ( \nabla ^A_\gamma \lambda ^{\beta }_\alpha {\bar{\psi }})\\&\quad +\mathop {\textrm{Re}}\nolimits ({\bar{\lambda }}^{\beta }_\alpha \lambda _\sigma ^\gamma ) ( \nabla _\gamma V^\sigma -\nabla ^\sigma V_\gamma )-V^\gamma \mathop {\textrm{Re}}\nolimits ({\bar{\lambda }}^{\beta }_\alpha \nabla ^A_\gamma \psi ) \\&\quad -\mathop {\textrm{Re}}\nolimits (\lambda ^\gamma _\alpha {\bar{\lambda }}^{\beta }_\sigma ) \nabla _\gamma V^\sigma +\mathop {\textrm{Re}}\nolimits (\lambda ^{\gamma \sigma }{\bar{\lambda }}^{\beta }_\sigma )\nabla _\alpha V_\gamma +V^\gamma \mathop {\textrm{Re}}\nolimits (\nabla ^A_\gamma \lambda ^\sigma _\alpha {\bar{\lambda }}^{\beta }_\sigma ) \\&\quad -\mathop {\textrm{Re}}\nolimits ({\bar{\lambda }}^\sigma _\alpha \lambda ^\gamma _\sigma )\nabla _\gamma V^{\beta }+\mathop {\textrm{Re}}\nolimits ({\bar{\lambda }}^\sigma _\alpha \lambda ^{\gamma {\beta }})\nabla _\sigma V_\gamma +V^\gamma \mathop {\textrm{Re}}\nolimits ({\bar{\lambda }}^\sigma _\alpha \nabla ^A_\gamma \lambda ^{\beta }_\sigma ) \\&=\ {\widetilde{\text {Ric}}}_{\alpha \gamma }\nabla ^\gamma V^{\beta }-{\widetilde{\text {Ric}}}^{\gamma {\beta }}\nabla _\alpha V_\gamma -V^\gamma \nabla _\gamma {{\widetilde{\text {Ric}}}_{\alpha }}^{\beta }. \end{aligned}$$

Then we have

$$\begin{aligned} I_2-(\nabla ^\mu V^\nu +\nabla ^\nu V^\mu ){R_{\mu \alpha \nu }}^{\beta }+J_3=-T^1_{\alpha \gamma }\nabla ^\gamma V^{\beta }+T^{1,\gamma {\beta }}\nabla _\alpha V_\gamma +V^\gamma \nabla _\gamma T^{1,\ {\beta }}_{\alpha }. \end{aligned}$$

This concludes the proof of the $T^1$-equations. $\square$

#### The equation for $T^2$

 By the second Bianchi identities for the Riemannian curvature and the following equality

$$\begin{aligned}&\nabla _\delta {{\tilde{R}}}_{\sigma \gamma \alpha {\beta }}+\nabla _\sigma {{\tilde{R}}}_{\gamma \delta \alpha {\beta }}+\nabla _\gamma {{\tilde{R}}}_{\delta \sigma \alpha {\beta }}\\&\quad =\ \mathop {\textrm{Re}}\nolimits (T^3_{\delta \gamma ,{\beta }}{\bar{\lambda }}_{\alpha \sigma }+T^3_{\delta \sigma ,\alpha }{\bar{\lambda }}_{{\beta }\gamma }-T^3_{\delta \gamma ,\alpha }{\bar{\lambda }}_{{\beta }\sigma }-T^3_{\delta \sigma ,{\beta }}{\bar{\lambda }}_{\alpha \gamma }+T^3_{\sigma \gamma ,\alpha }{\bar{\lambda }}_{{\beta }\delta }-T^3_{\sigma \gamma ,{\beta }}{\bar{\lambda }}_{\alpha \delta }), \end{aligned}$$

we have the counterpart of the second Bianchi identities

$$\begin{aligned} \nabla _\delta T^2_{\sigma \gamma \alpha {\beta }} + \nabla _{\sigma } T^2_{\gamma \delta \alpha {\beta }} + \nabla _{\gamma } T^2_{\delta \sigma \alpha {\beta }} = T^1\lambda , \end{aligned}$$

which combine with the algebraic symmetries of the same tensor to yield an elliptic system for $T^2$. Precisely, using the above relation we have

$$\begin{aligned} \nabla ^{\sigma }T^2_{\sigma \gamma \alpha {\beta }} = \nabla _{\alpha } T^1_{\gamma {\beta }} - \nabla _{{\beta }} T^1_{\gamma \alpha } + T^1\lambda , \end{aligned}$$

which combined with the previous one yields the desired elliptic system, with $T^1$ viewed as a source term. $\square$

#### The equations for $T^3$

 This has the form

$$\begin{aligned} \begin{aligned} (i\partial ^B_t -\Delta ^A_g)T^3_{\alpha {\beta },\gamma }&=\ \lambda T^5+V\nabla T^3+T^3(\nabla V+\lambda ^2+R)+(\nabla ^A\lambda +\lambda V)(T^1+T^2+T^4)\\&\quad +\nabla (T^2+T^4)\lambda . \end{aligned} \end{aligned}$$

Recall the $\lambda$-equations

$$\begin{aligned}&i\partial ^B_t \lambda _{{\beta }\gamma }+\nabla ^A_\mu \nabla ^{A,\mu } \lambda _{{\beta }\gamma }-\frac{1}{2}\left( {\widetilde{\text {Ric}}}_{{\beta }\delta }\lambda ^\delta _\gamma +{\widetilde{\text {Ric}}}_{\gamma \delta }\lambda ^\delta _{\beta }\right) + {{\tilde{R}}}_{{\beta }\sigma \gamma \delta }\lambda ^{\sigma \delta }+\frac{i}{2}\left( \tilde{{\textbf{F}}}_{{\beta }\delta }\lambda ^\delta _\gamma +\tilde{{\textbf{F}}}_{\gamma \delta }\lambda ^\delta _{\beta }\right) \\&\quad -\frac{i}{2}\lambda ^\delta _{\beta }\left[ \mathop {\textrm{Im}}\nolimits (\psi {\bar{\lambda }}_{\delta \gamma })+2\nabla _\gamma V_\delta \right] -\frac{i}{2}\lambda ^\delta _\gamma \left[ \mathop {\textrm{Im}}\nolimits (\psi {\bar{\lambda }}_{\delta {\beta }})+2\nabla _{\beta }V_\delta \right] -iV^\delta \nabla ^A_\delta \lambda _{{\beta }\gamma }=0. \end{aligned}$$

Applying $\nabla ^A_\alpha$ and $\nabla ^A_{\beta }$ to the above $\lambda _{{\beta }\gamma }$ and $\lambda _{\alpha \gamma }$-equations respectively, we obtain the difference

$$\begin{aligned} 0&=\ \Big [\nabla ^A_\alpha \Big (i\partial ^B_t \lambda _{{\beta }\gamma }+\nabla ^A_\mu \nabla ^{A,\mu } \lambda _{{\beta }\gamma }\Big )-\nabla ^A_{\beta }\Big (i\partial ^B_t \lambda _{\alpha \gamma }+\nabla ^A_\mu \nabla ^{A,\mu } \lambda _{\alpha \gamma }\Big )\Big ]\\&\quad +\Big [\nabla ^A_\alpha \Big [-\frac{1}{2}\Big ({\widetilde{\text {Ric}}}_{{\beta }\delta }\lambda ^\delta _\gamma +{\widetilde{\text {Ric}}}_{\gamma \delta }\lambda ^\delta _{\beta }\Big ) +{{\tilde{R}}}_{{\beta }\sigma \gamma \delta }\lambda ^{\sigma \delta }+\frac{i}{2}\Big (\tilde{{\textbf{F}}}_{{\beta }\delta }\lambda ^\delta _\gamma +\tilde{{\textbf{F}}}_{\gamma \delta }\lambda ^\delta _{\beta }\Big )\Big ]\\&\quad -\nabla ^A_{\beta }\Big [-\frac{1}{2}\Big ({\widetilde{\text {Ric}}}_{\alpha \delta }\lambda ^\delta _\gamma +{\widetilde{\text {Ric}}}_{\gamma \delta }\lambda ^\delta _\alpha \Big ) +{{\tilde{R}}}_{\alpha \sigma \gamma \delta }\lambda ^{\sigma \delta }+\frac{i}{2}\Big (\tilde{{\textbf{F}}}_{\alpha \delta }\lambda ^\delta _\gamma +\tilde{{\textbf{F}}}_{\gamma \delta }\lambda ^\delta _\alpha \Big )\Big ]\Big ]\\&\quad +\Big [\nabla ^A_\alpha \Big [-\frac{i}{2}\lambda ^\delta _{\beta }[\mathop {\textrm{Im}}\nolimits (\psi {\bar{\lambda }}_{\delta \gamma })+2\nabla _\gamma V_\delta ]-\frac{i}{2}\lambda ^\delta _\gamma [\mathop {\textrm{Im}}\nolimits (\psi {\bar{\lambda }}_{\delta {\beta }})+2\nabla _{\beta }V_\delta ]-iV^\delta \nabla ^A_\delta \lambda _{{\beta }\gamma }\Big ]\\&\quad -\nabla ^A_{\beta }\Big [-\frac{i}{2}\lambda ^\delta _\alpha [\mathop {\textrm{Im}}\nolimits (\psi {\bar{\lambda }}_{\delta \gamma })+2\nabla _\gamma V_\delta ]-\frac{i}{2}\lambda ^\delta _\gamma [\mathop {\textrm{Im}}\nolimits (\psi {\bar{\lambda }}_{\delta \alpha })+2\nabla _\alpha V_\delta ]-iV^\delta \nabla ^A_\delta \lambda _{\alpha \gamma }\Big ]\Big ]\\&:=\ I+II+III. \end{aligned}$$

We first compute *I*. We commute $\nabla ^A_\alpha$ with $\partial ^B_t$ and $\nabla ^A_\mu \nabla ^{A,\mu }$ to give 
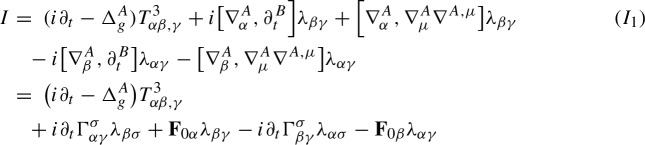

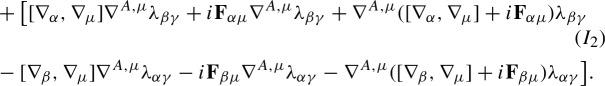
 For $I_1$, by the formulas for $\partial _t \Gamma$ in (8.14), for $G_{\mu \nu }$ in (8.13) and for the commutators $[\nabla _\alpha ,\nabla _{\beta }]$ we have

$$\begin{aligned} I_1&=\ i(\nabla _\alpha G_{\gamma \delta }+\nabla _\gamma G_{\alpha \delta }-\nabla _\delta G_{\alpha \gamma })\lambda ^\delta _{\beta }-i(\nabla _{\beta }G_{\gamma \delta }+\nabla _\gamma G_{{\beta }\delta }-\nabla _\delta G_{{\beta }\gamma })\lambda ^\delta _\alpha +\nabla T^1 \lambda \\&\quad +T^5_\alpha \lambda _{{\beta }\gamma }-T^5_{\beta }\lambda _{\alpha \gamma }+(\mathop {\textrm{Re}}\nolimits (\lambda ^\sigma _\alpha \overline{\nabla ^A_\sigma \psi })-\tilde{{\textbf{F}}}_{\alpha \sigma }V^\sigma )\lambda _{{\beta }\gamma }-(\mathop {\textrm{Re}}\nolimits (\lambda ^\sigma _{\beta }\overline{\nabla ^A_\sigma \psi })-\tilde{{\textbf{F}}}_{{\beta }\sigma }V^\sigma )\lambda _{\alpha \gamma }\\&=\ \nabla T^1 \lambda + T^5_\alpha \lambda _{{\beta }\gamma }-T^5_{\beta }\lambda _{\alpha \gamma }+I_{11}+I_{12}, \end{aligned}$$

where $I_{11}$, $I_{12}$ are the terms containing $\lambda \lambda \nabla \lambda$ and $V\lambda$ respectively,

$$\begin{aligned} I_{11}&:=\ i(\nabla _\alpha \mathop {\textrm{Im}}\nolimits (\psi {\bar{\lambda }}_{\gamma \delta })+\nabla _\gamma \mathop {\textrm{Im}}\nolimits (\psi {\bar{\lambda }}_{\alpha \delta })-\nabla _\delta \mathop {\textrm{Im}}\nolimits (\psi {\bar{\lambda }}_{\alpha \gamma }))\lambda ^\delta _{\beta }\\&\quad -i(\nabla _{\beta }\mathop {\textrm{Im}}\nolimits (\psi {\bar{\lambda }}_{\gamma \delta })+\nabla _\gamma \mathop {\textrm{Im}}\nolimits (\psi {\bar{\lambda }}_{{\beta }\delta })-\nabla _\delta \mathop {\textrm{Im}}\nolimits (\psi {\bar{\lambda }}_{{\beta }\gamma }))\lambda ^\delta _\alpha \\&\quad +\mathop {\textrm{Re}}\nolimits (\lambda ^\sigma _\alpha \overline{\nabla ^A_\sigma \psi })\lambda _{{\beta }\gamma }-\mathop {\textrm{Re}}\nolimits (\lambda ^\sigma _{\beta }\overline{\nabla ^A_\sigma \psi })\lambda _{\alpha \gamma },\\ I_{12}&:=\ \frac{i}{2}\big [(\nabla _\alpha \nabla _\gamma +\nabla _\gamma \nabla _\alpha )V_\delta \lambda ^\delta _{\beta }+R_{\alpha \sigma \gamma \delta }V^\delta \lambda ^\sigma _{\beta }+R_{\gamma \sigma \alpha \delta }V^\delta \lambda ^\sigma _{\beta }\big ]\\&\quad -\frac{i}{2}\big [(\nabla _{\beta }\nabla _\gamma +\nabla _\gamma \nabla _{\beta })V_\delta \lambda ^\delta _\alpha +R_{{\beta }\sigma \gamma \delta }V^\delta \lambda ^\sigma _\alpha +R_{\gamma \sigma {\beta }\delta }V^\delta \lambda ^\sigma _\alpha \big ]\\&\quad -\tilde{{\textbf{F}}}_{\alpha \sigma }V^\sigma \lambda _{{\beta }\gamma }+\tilde{{\textbf{F}}}_{{\beta }\sigma }V^\sigma \lambda _{\alpha \gamma }. \end{aligned}$$

Here, using the expressions for $\tilde{{\textbf{F}}}_{\alpha {\beta }}$ and ${{\tilde{R}}}_{{\beta }\alpha \gamma \delta }$, the expression $I_{11}$ can be rewritten as

$$\begin{aligned} I_{11}= i\nabla _\alpha \mathop {\textrm{Im}}\nolimits (\psi {\bar{\lambda }}_{\gamma \delta })\lambda ^\delta _{\beta }-i\nabla _{\beta }\mathop {\textrm{Im}}\nolimits (\psi {\bar{\lambda }}_{\gamma \delta })\lambda ^\delta _\alpha -i\nabla ^A_\gamma \psi \tilde{{\textbf{F}}}_{\alpha {\beta }}+\nabla ^A_\delta \psi {{\tilde{R}}}_{{\beta }\alpha \gamma \delta }, \end{aligned}$$

Using commutators $[\nabla _\gamma ,\nabla _\alpha ]$ and the Bianchi identities, the $I_{12}$ expression can be rewritten as

$$\begin{aligned} I_{12}&=i\nabla _\alpha \nabla _\gamma V_\delta \lambda ^\delta _{\beta }+i R_{\gamma \sigma \alpha \delta }V^\delta \lambda ^\sigma _{\beta }-i\nabla _{\beta }\nabla _\gamma V_\delta \lambda ^\delta _\alpha \\&\quad -i R_{\gamma \sigma {\beta }\delta }V^\delta \lambda ^\sigma _\alpha -\tilde{{\textbf{F}}}_{\alpha \sigma }V^\sigma \lambda _{{\beta }\gamma }+\tilde{{\textbf{F}}}_{{\beta }\sigma }V^\sigma \lambda _{\alpha \gamma } \end{aligned}$$

For $I_2$, we use the Riemannian curvature tensor to write

$$\begin{aligned} I_2&=\ R_{\alpha \mu \mu \delta }\nabla ^{A,\delta }\lambda _{{\beta }\gamma }+R_{\alpha \mu {\beta }\delta }\nabla ^{A,\mu }\lambda ^\delta _\gamma +R_{\alpha \mu \gamma \delta }\nabla ^{A,\mu }\lambda ^\delta _{\beta }+i{\textbf{F}}_{\alpha \mu }\nabla ^{A,\mu }\lambda _{{\beta }\gamma }\\&\quad +\nabla ^{A,\mu }(R_{\alpha \mu {\beta }\delta }\lambda ^\delta _\gamma +R_{\alpha \mu \gamma \delta }\lambda ^\delta _{\beta }+i{\textbf{F}}_{\alpha \mu }\lambda _{{\beta }\gamma })\\&\quad -R_{{\beta }\mu \mu \delta }\nabla ^{A,\delta }\lambda _{\alpha \gamma }-R_{{\beta }\mu \alpha \delta }\nabla ^{A,\mu }\lambda ^\delta _\gamma -R_{{\beta }\mu \gamma \delta }\nabla ^{A,\mu }\lambda ^\delta _\alpha -i{\textbf{F}}_{{\beta }\mu }\nabla ^{A,\mu }\lambda _{\alpha \gamma }\\&\quad -\nabla ^{A,\mu }(R_{{\beta }\mu \alpha \delta }\lambda ^\delta _\gamma +R_{{\beta }\mu \gamma \delta }\lambda ^\delta _\alpha +i{\textbf{F}}_{{\beta }\mu }\lambda _{\alpha \gamma })\\&= -{\text {Ric}}_{\alpha \delta }\nabla ^{A,\delta }\lambda _{{\beta }\gamma }+2R_{\alpha \mu {\beta }\delta }\nabla ^{A,\mu }\lambda ^\delta _\gamma +2R_{\alpha \mu \gamma \delta }\nabla ^{A,\mu }\lambda ^\delta _{\beta }+2i{\textbf{F}}_{\alpha \mu }\nabla ^{A,\mu }\lambda _{{\beta }\gamma }\\&\quad +\nabla ^\mu R_{\alpha \mu {\beta }\delta }\lambda ^\delta _\gamma +\nabla ^\mu R_{\alpha \mu \gamma \delta }\lambda ^\delta _{\beta }+i\nabla ^\mu {\textbf{F}}_{\alpha \mu }\lambda _{{\beta }\gamma }\\&\quad +{\text {Ric}}_{{\beta }\delta }\nabla ^{A,\delta }\lambda _{\alpha \gamma }-2R_{{\beta }\mu \alpha \delta }\nabla ^{A,\mu }\lambda ^\delta _\gamma -2R_{{\beta }\mu \gamma \delta }\nabla ^{A,\mu }\lambda ^\delta _\alpha -2i{\textbf{F}}_{{\beta }\mu }\nabla ^{A,\mu }\lambda _{\alpha \gamma }\\&\quad -\nabla ^\mu R_{{\beta }\mu \alpha \delta }\lambda ^\delta _\gamma -\nabla ^\mu R_{{\beta }\mu \gamma \delta }\lambda ^\delta _\alpha -i\nabla ^\mu {\textbf{F}}_{{\beta }\mu }\lambda _{\alpha \gamma }\\&=\ 2R_{\alpha \mu {\beta }\delta }{T^{3,\mu \delta }}_{,\gamma }+(T^1+T^2+T^4)\nabla ^A\lambda +\nabla (T^2+T^4)\lambda +J_1, \end{aligned}$$

where the terms in $J_1$ have the form $\lambda \lambda \nabla \lambda$ as

$$\begin{aligned} J_1&=\ \nabla ^{A,\delta }\lambda _{{\beta }\gamma }(-{\widetilde{\text {Ric}}}_{\alpha \delta }+2i\tilde{{\textbf{F}}}_{\alpha \delta })+\nabla ^{A,\delta }\lambda _{\alpha \gamma }({\widetilde{\text {Ric}}}_{{\beta }\delta }-2i\tilde{{\textbf{F}}}_{{\beta }\delta })\\&\quad +i\nabla ^\mu \tilde{{\textbf{F}}}_{\alpha \mu }\lambda _{{\beta }\gamma }-i\nabla ^\mu \tilde{{\textbf{F}}}_{{\beta }\mu }\lambda _{\alpha \gamma }\\&\quad +2{{\tilde{R}}}_{\alpha \mu \gamma \delta }\nabla ^{A,\mu }\lambda ^\delta _{\beta }+\nabla ^\mu {{\tilde{R}}}_{\alpha \mu {\beta }\delta }\lambda ^\delta _\gamma +\nabla ^\mu {{\tilde{R}}}_{\alpha \mu \gamma \delta }\lambda ^\delta _{\beta }\\&\quad -2{{\tilde{R}}}_{{\beta }\mu \gamma \delta }\nabla ^{A,\mu }\lambda ^\delta _\alpha -\nabla ^\mu {{\tilde{R}}}_{{\beta }\mu \alpha \delta }\lambda ^\delta _\gamma -\nabla ^\mu {{\tilde{R}}}_{{\beta }\mu \gamma \delta }\lambda ^\delta _\alpha . \end{aligned}$$

We next rewrite the *III* expression as

$$\begin{aligned} III&=\ \nabla ^A_\alpha \Big [-\frac{i}{2}\lambda ^\delta _{\beta }[\mathop {\textrm{Im}}\nolimits (\psi {\bar{\lambda }}_{\delta \gamma })+2\nabla _\gamma V_\delta ]-\frac{i}{2}\lambda ^\delta _\gamma [\mathop {\textrm{Im}}\nolimits (\psi {\bar{\lambda }}_{\delta {\beta }})+2\nabla _{\beta }V_\delta ]-iV^\delta \nabla ^A_\delta \lambda _{{\beta }\gamma }\Big ]\\&\quad -\nabla ^A_{\beta }\Big [-\frac{i}{2}\lambda ^\delta _\alpha [\mathop {\textrm{Im}}\nolimits (\psi {\bar{\lambda }}_{\delta \gamma })+2\nabla _\gamma V_\delta ]-\frac{i}{2}\lambda ^\delta _\gamma [\mathop {\textrm{Im}}\nolimits (\psi {\bar{\lambda }}_{\delta \alpha })+2\nabla _\alpha V_\delta ]-iV^\delta \nabla ^A_\delta \lambda _{\alpha \gamma }\Big ]\\&=\ -\frac{i}{2} T^3_{\alpha {\beta },\delta }[\mathop {\textrm{Im}}\nolimits (\psi {\bar{\lambda }}^\delta _{\gamma })+2\nabla _\gamma V^\delta ]+III_1+III_2, \end{aligned}$$

where

$$\begin{aligned} III_1&:=\ -\frac{i}{2}\lambda ^\delta _{\beta }\nabla _\alpha \mathop {\textrm{Im}}\nolimits (\psi {\bar{\lambda }}_{\delta \gamma })-\frac{i}{2}\nabla ^A_\alpha (\lambda ^\delta _\gamma \mathop {\textrm{Im}}\nolimits (\psi {\bar{\lambda }}_{\delta {\beta }}))\\&\quad +\frac{i}{2}\lambda ^\delta _\alpha \nabla _{\beta }\mathop {\textrm{Im}}\nolimits (\psi {\bar{\lambda }}_{\delta \gamma })+\frac{i}{2}\nabla ^A_{\beta }(\lambda ^\delta _\gamma \mathop {\textrm{Im}}\nolimits (\psi {\bar{\lambda }}_{\delta \alpha })),\\ III_2&:=\ -i\lambda ^\delta _{\beta }\nabla _\alpha \nabla _\gamma V_\delta -i\nabla ^A_\alpha (\lambda ^\delta _\gamma \nabla _{\beta }V_\delta )-i\nabla ^A_\alpha (V^\delta \nabla ^A_\delta \lambda _{{\beta }\gamma })\\&\quad +i\lambda ^\delta _\alpha \nabla _{\beta }\nabla _\gamma V_\delta +i\nabla ^A_{\beta }(\lambda ^\delta _\gamma \nabla _\alpha V_\delta )+i\nabla ^A_{\beta }(V^\delta \nabla ^A_\delta \lambda _{\alpha \gamma }). \end{aligned}$$

The $I_{12}+III_2$ expression vanishes modulo $\{V\nabla T^3,T^3\nabla V,\lambda V(T^2+T^4)\}$. Precisely, we can further write $III_2$ as 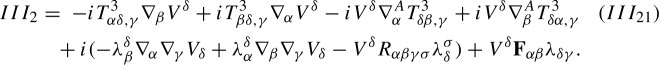 Then replacing $R_{\alpha {\beta }\gamma \delta }$, ${\textbf{F}}_{\alpha {\beta }}$ by ${{\tilde{R}}}_{\alpha {\beta }\gamma \delta }$ and $\tilde{{\textbf{F}}}_{\alpha {\beta }}$ respectively, we have 
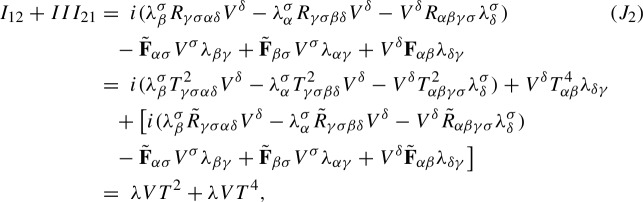
 where the term $J_2$ vanishes due to the representations of ${{\tilde{R}}}_{\gamma \sigma \alpha \delta }$ and $\tilde{{\textbf{F}}}_{\alpha \sigma }$.

Next, we show that the terms $I_{11}+J_1+II+III_1$ vanish modulo $\lambda \lambda T^3$. We have

$$\begin{aligned} I_{11}+III_1&=\ -i\nabla ^A_\gamma \psi \tilde{{\textbf{F}}}_{\alpha {\beta }}+\nabla ^A_\delta \psi {{\tilde{R}}}_{{\beta }\alpha \gamma \delta } +\frac{i}{2}\lambda ^\delta _{\beta }\nabla _\alpha \mathop {\textrm{Im}}\nolimits (\psi {\bar{\lambda }}_{\delta \gamma })-\frac{i}{2}\lambda ^\delta _\alpha \nabla _{\beta }\mathop {\textrm{Im}}\nolimits (\psi {\bar{\lambda }}_{\delta \gamma })\\&\quad -\frac{i}{2}\nabla ^A_\alpha (\lambda ^\delta _\gamma \mathop {\textrm{Im}}\nolimits (\psi {\bar{\lambda }}_{\delta {\beta }}))+\frac{i}{2}\nabla ^A_{\beta }(\lambda ^\delta _\gamma \mathop {\textrm{Im}}\nolimits (\psi {\bar{\lambda }}_{\delta \alpha })). \end{aligned}$$

We rewrite *II* as 
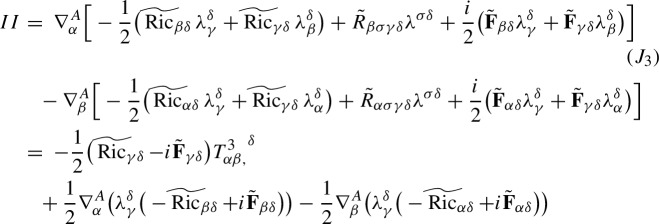

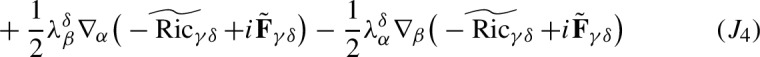

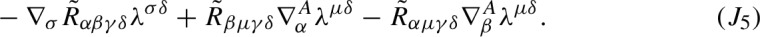
 Hence,

$$\begin{aligned}&I_{11}+III_1+J_3+J_4\\&\quad =-i\nabla ^A_\gamma \psi \tilde{{\textbf{F}}}_{\alpha {\beta }}+\nabla ^A_\delta \psi {{\tilde{R}}}_{{\beta }\alpha \gamma \delta } +\frac{1}{2}\lambda ^\delta _{\beta }\nabla _\alpha (-\lambda _{\gamma \delta }{\bar{\psi }}+\lambda _{\gamma \sigma }{\bar{\lambda }}^\sigma _\delta )\\&\qquad +\frac{1}{2}\lambda ^\delta _\alpha \nabla _{\beta }(\lambda _{\gamma \delta }{\bar{\psi }}-\lambda _{\gamma \sigma }{\bar{\lambda }}^\sigma _\delta )+\frac{1}{2}\nabla ^A_\alpha \big [\lambda ^\delta _\gamma \big (-{\bar{\lambda }}_{\delta {\beta }}\psi +\lambda _{{\beta }\sigma }{\bar{\lambda }}^\sigma _\delta \big )\big ]\\&\qquad +\frac{1}{2}\nabla ^A_{\beta }\big [\lambda ^\delta _\gamma \big ({\bar{\lambda }}_{\delta \alpha }\psi -\lambda _{\alpha \sigma }{\bar{\lambda }}^\sigma _\delta \big )\big ]\\&\quad =-i\nabla ^A_\gamma \psi \tilde{{\textbf{F}}}_{\alpha {\beta }}+\nabla ^A_\delta \psi {{\tilde{R}}}_{{\beta }\alpha \gamma \delta }-\nabla ^A_\alpha \lambda ^\delta _\gamma \big ( {\widetilde{\text {Ric}}}_{{\beta }\delta }-i\tilde{{\textbf{F}}}_{{\beta }\delta }\big )+\nabla ^A_{\beta }\lambda ^\delta _\gamma \big ({\widetilde{\text {Ric}}}_{\alpha \delta }-i\tilde{{\textbf{F}}}_{\alpha \delta }\big )\\&\qquad +\overline{\nabla ^A_\alpha \lambda ^{\sigma \delta }}\lambda _{{\beta }\sigma }\lambda _{\gamma \delta }-\overline{\nabla ^A_{\beta }\lambda ^{\sigma \delta }}\lambda _{\alpha \sigma }\lambda _{\gamma \delta } -\lambda ^\delta _\gamma \mathop {\textrm{Re}}\nolimits (\nabla ^A_\alpha \psi {\bar{\lambda }}_{{\beta }\delta })+\lambda ^\delta _\gamma \mathop {\textrm{Re}}\nolimits (\nabla ^A_{\beta }\psi {\bar{\lambda }}_{\alpha \delta })+\lambda ^2 T^3. \end{aligned}$$

Since by ${{\tilde{R}}}$ and $\tilde{{\textbf{F}}}$ we also have

$$\begin{aligned} J_1+J_5&= -\nabla ^{A,\delta }\lambda _{{\beta }\gamma }{\widetilde{\text {Ric}}}_{\alpha \delta }+\nabla ^{A,\delta }\lambda _{\alpha \gamma }{\widetilde{\text {Ric}}}_{{\beta }\delta }+\lambda ^\delta _\gamma \mathop {\textrm{Re}}\nolimits (\nabla ^A_\alpha \psi {\bar{\lambda }}_{{\beta }\delta }-\nabla ^A_{\beta }\psi {\bar{\lambda }}_{\alpha \delta })\\&\quad -\overline{\nabla ^A_\alpha \lambda ^{\mu \sigma }}\lambda _{\mu {\beta }}\lambda _{\sigma \gamma }+\overline{\nabla ^A_{\beta }\lambda ^{\mu \sigma }}\lambda _{\mu \alpha }\lambda _{\sigma \gamma }-i\nabla ^{A,\delta }\lambda _{\alpha \gamma }\tilde{{\textbf{F}}}_{{\beta }\delta }+i\nabla ^{A,\delta }\lambda _{{\beta }\gamma }\tilde{{\textbf{F}}}_{\alpha \delta }\\&\quad +\nabla ^{A,\sigma }\psi {{\tilde{R}}}_{\alpha {\beta }\gamma \sigma }+i\nabla ^A_\gamma \psi \tilde{{\textbf{F}}}_{\alpha {\beta }}+\lambda ^2 T^3. \end{aligned}$$

Then in the above two formulas all terms cancel except for $\lambda \lambda T^3$. Hence, we obtain that $I_{11}+J_1+II+III_1$ vanishes modulo $\lambda \lambda T^3$. This concludes the proof of the $T^3$-equations. $\square$

#### The equations for $T^4$

 These have the form

$$\begin{aligned} \begin{aligned} (\partial _t -\Delta _g)T^4_{\alpha {\beta }}&=-{\text {Ric}}_{\alpha \delta } {T^{4,\delta }}_{\beta } + {\text {Ric}}_{\beta \delta } {T^{4,\delta }}_{\alpha } - R_{\beta \alpha \sigma \delta } T^{2,\sigma \delta }\\&\quad -\mathop {\textrm{Re}}\nolimits (\nabla ^{A,\sigma }\psi \overline{T^3_{\alpha {\beta },\sigma }})-V^\gamma \mathop {\textrm{Im}}\nolimits (\lambda _{\gamma }^\sigma \overline{T^3_{\alpha {\beta },\sigma }})+V^\gamma \mathop {\textrm{Im}}\nolimits (T^3_{\gamma \alpha ,\sigma }{\bar{\lambda }}^\sigma _{\beta }). \end{aligned} \end{aligned}$$

By the *A*-equations we have

$$\begin{aligned} (\partial _t -\Delta _g)T^4_{\alpha {\beta }}&= -[\Delta _g,\nabla _\alpha ]A_{\beta }+[\Delta _g,\nabla _{\beta }]A_\alpha -\nabla _\alpha \big ({\widetilde{\text {Ric}}}_{{\beta }\delta }A^\delta \big )+\nabla _{\beta }\big ({\widetilde{\text {Ric}}}_{\alpha \delta }A^\delta \big )\\&\quad +\nabla _\alpha \nabla ^\sigma \tilde{{\textbf{F}}}_{{\beta }\sigma }-\nabla _{\beta }\nabla ^\sigma \tilde{{\textbf{F}}}_{\alpha \sigma }-\Delta _g \tilde{{\textbf{F}}}_{\alpha {\beta }}\\&\quad -\partial _t \tilde{{\textbf{F}}}_{\alpha {\beta }} +\nabla _\alpha \big [\mathop {\textrm{Re}}\nolimits (\lambda ^\gamma _{\beta }\overline{\nabla ^A_\gamma \psi })-\tilde{{\textbf{F}}}_{{\beta }\delta }V^\delta \big ]-\nabla _{\beta }\big [\mathop {\textrm{Re}}\nolimits (\lambda ^\gamma _\alpha \overline{\nabla ^A_\gamma \psi })-\tilde{{\textbf{F}}}_{\alpha \delta }V^\delta \big ]\\&:=\ I_1+I_2+I_3. \end{aligned}$$

For the commutator we use the Bianchi identities to compute

$$\begin{aligned}&\ -[\nabla ^\sigma \nabla _\sigma ,\nabla _{\alpha }] A_\beta + [ \nabla ^\sigma \nabla _\sigma , \nabla _\beta ] A_\alpha \\&\quad = \ -\nabla ^\sigma (R_{\sigma \alpha \beta \delta } A^\delta - R_{\sigma \beta \alpha \delta } A^\delta ) - (R_{\sigma \alpha \beta \delta } - R_{\sigma \beta \alpha \delta }) \nabla ^\sigma A^\delta - {R^\sigma }_{\alpha \sigma \delta } \nabla ^\delta A_\beta +{R^\sigma }_{\beta \sigma \delta } \nabla ^\delta A_\alpha \\&\quad = \ -\nabla ^\sigma R_{\beta \alpha \sigma \delta } A^\delta - 2 R_{\beta \alpha \sigma \delta } \nabla ^\sigma A^\delta - {\text {Ric}}_{\alpha \delta } \nabla ^{\delta } A_\beta + {\text {Ric}}_{\beta \delta } \nabla ^{\delta } A_\alpha \\&\quad = \ -(\nabla _\beta {\text {Ric}}_{\alpha \delta } - \nabla _\alpha R_{\beta \delta } ) A^\delta - R_{\beta \alpha \sigma \delta } {\textbf{F}}^{\sigma \delta }- {\text {Ric}}_{\alpha \delta } ({{\textbf{F}}^\delta }_\beta +\nabla _{\beta } A^\delta ) + {\text {Ric}}_{\beta \delta } ({{\textbf{F}}^\delta }_\alpha + \nabla _{\alpha } A^\delta ) \\&\quad = \ -\nabla _\beta ( {\text {Ric}}_{\alpha \delta } A^\delta ) + \nabla _\alpha ({\text {Ric}}_{\beta \delta } A^\delta ) - R_{\beta \alpha \sigma \delta } {\textbf{F}}^{\sigma \delta }- {\text {Ric}}_{\alpha \delta } {{\textbf{F}}^\delta }_\beta + {\text {Ric}}_{\beta \delta } {{\textbf{F}}^\delta }_\alpha . \end{aligned}$$

We commute $\nabla _\alpha ,\ \nabla _{\beta }$ with $\nabla ^\sigma$ and use $\nabla _\alpha \tilde{{\textbf{F}}}_{{\beta }\sigma }+\nabla _{\beta }\tilde{{\textbf{F}}}_{\sigma \alpha }+\nabla _\sigma \tilde{{\textbf{F}}}_{\alpha {\beta }}=0$ to compute $I_2$ by

$$\begin{aligned} I_2&= \ R_{\alpha \sigma {\beta }\gamma } {\tilde{{\textbf{F}}}^{\gamma \sigma }} + R_{\alpha \sigma \sigma \gamma } \tilde{{\textbf{F}}}^{\ \gamma }_{{\beta }} - R_{\beta \sigma \alpha \gamma } \tilde{{\textbf{F}}}^{\gamma \sigma } - R_{\beta \sigma \sigma \gamma } \tilde{{\textbf{F}}}^{\ \gamma }_{\alpha } \\&= \ {\text {Ric}}_{\alpha \delta } {\tilde{{\textbf{F}}}^\delta }_{\ \beta } - {\text {Ric}}_{\beta \delta } {\tilde{{\textbf{F}}}^\delta }_{\ \alpha } + R_{\beta \alpha \sigma \delta } \tilde{{\textbf{F}}}^{\sigma \delta }. \end{aligned}$$

Then we obtain

$$\begin{aligned} I_1+I_2=-{\text {Ric}}_{\alpha \delta } {T^{4,\delta }}_{\beta } + {\text {Ric}}_{\beta \delta } {T^{4,\delta }}_{\alpha } - R_{\beta \alpha \sigma \delta } T^{4,\sigma \delta } \end{aligned}$$

For $I_3$ we compute $\partial _t \tilde{{\textbf{F}}}_{\alpha {\beta }}$ first.

$$\begin{aligned} \partial _t \tilde{{\textbf{F}}}_{\alpha {\beta }}=\mathop {\textrm{Im}}\nolimits (\partial _t \lambda _{\alpha \sigma }{\bar{\lambda }}^\sigma _{\beta }-\partial _t \lambda _{{\beta }\sigma }{\bar{\lambda }}^\sigma _\alpha )+\partial _t g^{\sigma \mu }\mathop {\textrm{Im}}\nolimits (\lambda _{\alpha \sigma }{\bar{\lambda }}_{{\beta }\mu }) \end{aligned}$$

By the *g*-equations and

$$\begin{aligned} i\partial ^{B}_t\lambda _{\alpha {\beta }}+\nabla ^A_{\alpha }\nabla ^{A}_{\beta }\psi -i\lambda ^{\gamma }_{\alpha }\mathop {\textrm{Im}}\nolimits (\psi {\bar{\lambda }}_{\gamma {\beta }})-i\lambda ^{\gamma }_{\alpha }\nabla _{{\beta }} V_{\gamma }-i\lambda _{\beta }^\gamma \nabla _\alpha V_\gamma -iV^\gamma \nabla ^A_\gamma \lambda _{\alpha {\beta }}=0, \end{aligned}$$

we have

$$\begin{aligned} \mathop {\textrm{Im}}\nolimits (\partial _t \lambda _{\alpha \sigma }{\bar{\lambda }}^\sigma _{\beta })&=-\mathop {\textrm{Re}}\nolimits (B\lambda _{\alpha \sigma }{\bar{\lambda }}^\sigma _{\beta }-\nabla ^A_\alpha \nabla ^A_\sigma \psi {\bar{\lambda }}^\sigma _{\beta })+\mathop {\textrm{Im}}\nolimits (\lambda ^\gamma _\alpha {\bar{\lambda }}^\sigma _{\beta })(\mathop {\textrm{Im}}\nolimits (\psi {\bar{\lambda }}_{\gamma \sigma })+\nabla _\sigma V_\gamma )\\&\quad +\nabla _\alpha (\tilde{{\textbf{F}}}^\gamma _{\ {\beta }}V_\gamma )-V^\gamma \mathop {\textrm{Im}}\nolimits (\lambda _{\gamma \sigma }\overline{\nabla ^A_\alpha \lambda ^\sigma _{\beta }})+V^\gamma \mathop {\textrm{Im}}\nolimits (T^1_{\gamma \alpha ,\sigma }{\bar{\lambda }}^\sigma _{\beta }) \end{aligned}$$

Then we rewrite the expression $\partial _t \tilde{{\textbf{F}}}_{\alpha {\beta }}$ as

$$\begin{aligned} \begin{aligned} \partial _t \tilde{{\textbf{F}}}_{\alpha {\beta }}&=\ \nabla _\alpha \mathop {\textrm{Re}}\nolimits (\nabla ^A_\sigma \psi {\bar{\lambda }}^\sigma _{\beta }) -\nabla _{\beta }\mathop {\textrm{Re}}\nolimits (\nabla ^A_\sigma \psi {\bar{\lambda }}^\sigma _\alpha )-\mathop {\textrm{Re}}\nolimits (\nabla ^{A,\sigma }\psi \overline{T^3_{\alpha {\beta },\sigma }})\\&\quad +\nabla _\alpha (\tilde{{\textbf{F}}}^\gamma _{\ {\beta }}V_\gamma )-\nabla _{\beta }(\tilde{{\textbf{F}}}^\gamma _{\ \alpha }V_\gamma )-V^\gamma \mathop {\textrm{Im}}\nolimits (\lambda _{\gamma }^\sigma \overline{T^3_{\alpha {\beta },\sigma }})+V^\gamma \mathop {\textrm{Im}}\nolimits (T^3_{\gamma \alpha ,\sigma }{\bar{\lambda }}^\sigma _{\beta })\\&=\ \nabla _\alpha \tilde{{\textbf{F}}}_{0{\beta }}-\nabla _{\beta }\tilde{{\textbf{F}}}_{0\alpha }+\nabla \psi T^3+\lambda V T^3. \end{aligned} \end{aligned}$$

Hence, we have

$$\begin{aligned} I_3&=\ \nabla _\alpha \mathop {\textrm{Re}}\nolimits (\nabla ^A_\sigma \psi {\bar{\lambda }}^\sigma _{\beta }) -\nabla _{\beta }\mathop {\textrm{Re}}\nolimits (\nabla ^A_\sigma \psi {\bar{\lambda }}^\sigma _\alpha )-\mathop {\textrm{Re}}\nolimits (\nabla ^{A,\sigma }\psi \overline{T^1_{\alpha {\beta },\sigma }})\\&\quad +\nabla _\alpha (\tilde{{\textbf{F}}}^\gamma _{\ {\beta }}V_\gamma )-\nabla _{\beta }(\tilde{{\textbf{F}}}^\gamma _{\ \alpha }V_\gamma )-V^\gamma \mathop {\textrm{Im}}\nolimits (\lambda _{\gamma }^\sigma \overline{T^1_{\alpha {\beta },\sigma }})+V^\gamma \mathop {\textrm{Im}}\nolimits (T^3_{\gamma \alpha ,\sigma }{\bar{\lambda }}^\sigma _{\beta })\\&\quad +\nabla _\alpha [\mathop {\textrm{Re}}\nolimits (\lambda ^\gamma _{\beta }\overline{\nabla ^A_\gamma \psi })-\tilde{{\textbf{F}}}_{{\beta }\delta }V^\delta ]-\nabla _{\beta }[\mathop {\textrm{Re}}\nolimits (\lambda ^\gamma _\alpha \overline{\nabla ^A_\gamma \psi })-\tilde{{\textbf{F}}}_{\alpha \delta }V^\delta ]\\&= -\mathop {\textrm{Re}}\nolimits (\nabla ^{A,\sigma }\psi \overline{T^3_{\alpha {\beta },\sigma }})-V^\gamma \mathop {\textrm{Im}}\nolimits (\lambda _{\gamma }^\sigma \overline{T^3_{\alpha {\beta },\sigma }})+V^\gamma \mathop {\textrm{Im}}\nolimits (T^3_{\gamma \alpha ,\sigma }{\bar{\lambda }}^\sigma _{\beta }) \end{aligned}$$

This concludes the proof $T^4$-equations. $\square$

## The Reconstruction of the Flow

In this last section we close the circle of ideas in this paper, and prove that one can start from the good gauge solution given by Theorem 2.5, and reconstruct the flow at the level of *d*-dimensional embedded submanifolds. For completeness, we provide here another, more complete statement of our main theorem.

### Theorem 9.1

(Small data local well-posedness) Let $d\geqq 2$ and $s>\frac{d}{2}$. Consider the skew mean curvature flow (1.1) for maps *F* from ${{\mathbb {R}}}^d$ to the Euclidean space $({{\mathbb {R}}}^{d+2},g_{{{\mathbb {R}}}^{d+2}})$ with initial data $\Sigma _0$ which, in some coordinates, has a metric $g_0$ satisfying $\Vert |D|^{\sigma _d}(g_0-I_d)\Vert _{H^{s+1-\sigma _d}}\leqq \epsilon _0$ and mean curvature $\Vert {\textbf{H}}_0 \Vert _{H^s(\Sigma _0)}\leqq \epsilon _0$. In addition, we assume that $\Vert g_0-I_d\Vert _{Y^{lo}_0}\lesssim \epsilon _0$ in dimension $d= 2$.

If $\epsilon _0>0$ is sufficiently small, then there exists a unique solution

$$\begin{aligned} F: {{\mathbb {R}}}^d \times [0,1] \rightarrow ({{\mathbb {R}}}^{d+2},g_{{{\mathbb {R}}}^{d+2}}) \end{aligned}$$

which, when represented in harmonic coordinates at the initial time and heat coordinates dynamically, has regularity

$$\begin{aligned} \partial _x^2 F,\ \partial _t F \in C([0,1]; H^{s}({{\mathbb {R}}}^d)). \end{aligned}$$

and induced metric and mean curvature

$$\begin{aligned} |D|^{\sigma _d}(g-I_d) \in C([0,1]; H^{s+2-\sigma _d}({{\mathbb {R}}}^d)), \quad {\textbf{H}} \in C([0,1]; H^{s}({{\mathbb {R}}}^d)). \end{aligned}$$

In addition the mean curvature satisfies the bounds

$$\begin{aligned} \Vert \lambda \Vert _{l^2 X^s} + \Vert (h,A)\Vert _{{{\varvec{ {\mathcal {E}}}}}^s}\lesssim \Vert \lambda _0\Vert _{H^s}+\Vert h_0\Vert _{{\textbf{Y}}_0^{s+2}}. \end{aligned}$$

where $\lambda$ and *A* are expressed using the Coulomb gauge initially and the heat gauge dynamically in the normal bundle $N \Sigma _t$.

We prove the theorem in several steps.

### The Moving Frame

Once we have the initial data $(h_0,A_0,\lambda _0)$ which is small in ${{\mathcal {H}}}^s\times H^s$ by Proposition 4.1 and 4.2, Theorem 2.5 yields the good gauge local solution $\lambda$, along with the associated derived variables (*h*, *A*). But this does not yet give us the actual maps *F*.

Here we undertake the task of reconstructing the frame $(F_\alpha , m)$. For this we use the system consisting of (2.6) and (2.14), viewed as a linear ode. We recall these equations here:

9.1 $$\begin{aligned} \left\{ \begin{aligned}&\partial _{\alpha }F_{{\beta }}=\Gamma ^{\gamma }_{\alpha {\beta }}F_{\gamma }+\mathop {\textrm{Re}}\nolimits (\lambda _{\alpha {\beta }}{\bar{m}}),\\&\partial _{\alpha }^A m=-\lambda ^{\gamma }_{\alpha } F_{\gamma }, \end{aligned}\right. \end{aligned}$$

respectively

9.2 $$\begin{aligned} \left\{ \begin{aligned}&\partial _t F_{\alpha }=-\mathop {\textrm{Im}}\nolimits (\partial ^A_{\alpha } \psi {\bar{m}}-i\lambda _{\alpha \gamma }V^{\gamma } {\bar{m}})+[\mathop {\textrm{Im}}\nolimits (\psi {\bar{\lambda }}^{\gamma }_{\alpha })+\nabla _{\alpha } V^{\gamma }]F_{\gamma },\\&\partial ^{B}_t m=-i(\partial ^{A,\alpha } \psi -i\lambda ^{\alpha }_{\gamma }V^{\gamma } )F_{\alpha }. \end{aligned}\right. \end{aligned}$$

We start with the frame at time $t=0$, which already is known to solve (9.1), and has the following properties:

(i) *Orthogonality*, $F_\alpha \perp m$, $\langle m,m\rangle =2$, $\langle m,{\bar{m}}\rangle =0$ and consistency with the metric $g_{\alpha {\beta }} = \langle F_\alpha ,F_{\beta }\rangle$.
(ii) *Integrability*, $$\partial _\beta F_\alpha = \partial _\alpha F_\beta $$.
(iii) *Consistency* with the second fundamental form and the connection *A*:
  $$\begin{aligned} \partial _\alpha F_{\beta }\cdot m=\lambda _{\alpha {\beta }}, \qquad \langle \partial _\alpha m,m\rangle = -2 i A_\alpha . \end{aligned}$$

Next we extend this frame to times $t > 0$ by simultaneously solving the pair of equations (9.1) and (9.2).

#### The Solvability of (9.1) and (9.2)

The system consisting of (9.1) and (9.2) is overdetermined, and the necessary and sufficient condition for existence of solutions is provided by Frobenius’ theorem. We now verify these compatibility conditions in two steps:

- Compatibility conditions for the system (9.1) at fixed time. Here, by $T^2_{\alpha {\beta }\mu \nu }=0$, $T^3_{\alpha {\beta },\gamma }=0$, $T^4_{\alpha {\beta }}=0$ and we have
  $$\begin{aligned}&\partial _{\alpha }(\Gamma ^\sigma _{{\beta }\gamma }F_\sigma +\mathop {\textrm{Re}}\nolimits (\lambda _{{\beta }\gamma }{\bar{m}}))-\partial _{{\beta }}(\Gamma ^\sigma _{\alpha \gamma }F_\sigma +\mathop {\textrm{Re}}\nolimits (\lambda _{\alpha \gamma }{\bar{m}})) =0, \end{aligned}$$
  and
  $$\begin{aligned} \partial _\alpha (iA_{\beta }m+\lambda ^\sigma _{\beta }F_\sigma )-\partial _{\beta }(iA_\alpha m+\lambda ^\sigma _\alpha F_\sigma )=0, \end{aligned}$$
  as needed.
- Compatibility conditions between the system (9.1) and (9.2). By (9.1), (9.2) and (8.15) we have
  $$\begin{aligned} \partial _t(iA_\alpha m+\lambda ^\sigma _\alpha F_\sigma )-\partial _\alpha (iB m+i(\partial ^{A,\sigma }\psi -i\lambda ^\sigma _\gamma V^\gamma )F_\sigma ) =0 \end{aligned}$$
  and
  $$\begin{aligned}{} & {} \partial _{\beta }[-\mathop {\textrm{Im}}\nolimits (\partial ^A_{\alpha } \psi {\bar{m}}-i\lambda _{\alpha \gamma }V^{\gamma } {\bar{m}})+[\mathop {\textrm{Im}}\nolimits (\psi {\bar{\lambda }}^{\gamma }_{\alpha })+\nabla _{\alpha } V^{\gamma }]F_{\gamma }]\\ {}{} & {} \quad -\partial _t[\Gamma ^\gamma _{{\beta }\alpha }F_\gamma +\mathop {\textrm{Re}}\nolimits (\lambda _{{\beta }\alpha }{\bar{m}})] = 0. \end{aligned}$$

#### Solving the System (9.1)–(9.2) Locally

Starting from the existing frame at time $t=0$, we want to extend it forward in time by solving (9.2), while insuring that (9.1) remains valid. The difficulty is that we lack the uniform integrability in time for the coefficients in (9.2). However, in view of the local energy decay bounds for $\lambda$ and $\psi$, we do know that locally we have $\lambda \in L^2_t H^{s+\frac{1}{2}}$. We choose a distinguished coordinate, say $x_d$, and denote the remaining coordinates by $x'$. Then in view of Sobolev embeddings we have the local regularity

$$\begin{aligned} \partial \lambda \in C_{x_d} L^2_t H^{s-1}_{x'} \cap L^2_{x_d} L^2_t C_{x'} \end{aligned}$$

Thus on a “good" $x_d$ slice we have $\partial \lambda \in L^2_t C_{x'}$ and we can extend our frame forward in time as a continuous function, with $L^2_t L^\infty _{x'}$ time derivatives and bounded spatial derivatives.

At fixed time all the coefficients are continuous so we can start from the above $x_d$ slice and solve the system (9.1) globally in *x*, obtaining a global frame $(F_\alpha ,m)$ which is locally Lipschitz in *x* and continuous in *t*. By Frobenius’ theorem, this solution must also satisfy (9.2) on any good $x_d$ slice, which is a.e. Thus we have obtained the desired global frame $(F_\alpha ,m)$ for $t \in [0,1]$.

#### Propagating the Properties (i)–(iii)

Here we show that the properties (i)–(iii) above also extend to all $t \in [0,1]$. The properties (ii) and (iii) follow directly from the equations (9.1) and (9.2) once the orthogonality conditions in (i) are verified. We denote

$$\begin{aligned} {{{\tilde{g}}}}_{00}=\langle m,m\rangle ,\quad {{{\tilde{g}}}}_{\alpha 0}=\langle F_\alpha , m\rangle ,\quad {{{\tilde{g}}}}_{\alpha {\beta }}=\langle F_\alpha ,F_{\beta }\rangle . \end{aligned}$$

The first step is to propagate (i) forward in time on a good $x_d$ slice. Indeed, by (9.2) and (8.13) we have

$$\begin{aligned} \begin{aligned} \partial _t {{{\tilde{g}}}}_{\alpha 0}&=-\frac{i}{2}(\overline{\partial ^A_\alpha \psi }+i {\bar{\lambda }}_{\alpha \gamma }V^\gamma )({{{\tilde{g}}}}_{00}-2)-i(\overline{\partial ^{A,\sigma } \psi }+i {\bar{\lambda }}^\sigma _{\gamma }V^\gamma )(g_{\alpha \sigma }-{{{\tilde{g}}}}_{\alpha \sigma })\\&\quad +\frac{i}{2}(\partial ^A_\alpha \psi +i \lambda _{\alpha \gamma }V^\gamma )\langle {\bar{m}},m\rangle +(\mathop {\textrm{Im}}\nolimits (\psi {\bar{\lambda }}_\alpha ^\gamma )+\nabla _\alpha V^\gamma ){{{\tilde{g}}}}_{\gamma 0}+iB {{{\tilde{g}}}}_{\alpha 0}, \\ \partial _t ({{{\tilde{g}}}}_{00}-2)&=2\mathop {\textrm{Im}}\nolimits [(\partial ^{A,\alpha }\psi -i \lambda ^\alpha _\gamma V^\gamma ) {{{\tilde{g}}}}_{\alpha 0}],\\ \partial _t \langle m,{\bar{m}}\rangle&= -2iB\langle m,{\bar{m}}\rangle -2i (\partial ^{A,\alpha }\psi -i \lambda ^\alpha _\gamma V^\gamma ) {\bar{{{{\tilde{g}}}}}}_{\alpha 0},\\ \partial _t(g_{\alpha {\beta }}-{{{\tilde{g}}}}_{\alpha {\beta }})&=\ (\mathop {\textrm{Im}}\nolimits (\psi {\bar{\lambda }}_\alpha ^\gamma )+\nabla _\alpha V^\gamma )(g_{{\beta }\gamma }-{{{\tilde{g}}}}_{{\beta }\gamma })+(\mathop {\textrm{Im}}\nolimits (\psi {\bar{\lambda }}_{\beta }^\gamma )+\nabla _{\beta }V^\gamma )(g_{\alpha \gamma }-{{{\tilde{g}}}}_{\alpha \gamma })\\&\quad +\mathop {\textrm{Im}}\nolimits (\partial ^A_\alpha \psi {{{\tilde{g}}}}_{{\beta }0}-i\lambda _{\alpha \gamma }V^\gamma {{{\tilde{g}}}}_{{\beta }0})-\mathop {\textrm{Im}}\nolimits (\partial ^A_{\beta }\psi {\bar{{{{\tilde{g}}}}}}_{\alpha 0}-i\lambda _{{\beta }\gamma }V^\gamma {\bar{{{{\tilde{g}}}}}}_{\alpha 0}). \end{aligned} \end{aligned}$$

Viewed as a linear system of ode’s in time, these equations allow us to propagate (i) in time, given that it is satisfied at $t = 0$.

It remains to propagate (i) spatially. Using (9.1) we compute

$$\begin{aligned} \partial _\alpha {{{\tilde{g}}}}_{{\beta }0}&=\Gamma ^\gamma _{\alpha {\beta }} {{{\tilde{g}}}}_{\gamma 0}+\frac{1}{2}\lambda _{\alpha {\beta }}\langle {\bar{m}},m\rangle +\frac{1}{2}{\bar{\lambda }}_{\alpha {\beta }}({{{\tilde{g}}}}_{00}-2)+{\bar{\lambda }}^\gamma _\alpha (g_{{\beta }\gamma }-{{{\tilde{g}}}}_{{\beta }\gamma })+iA_\alpha {{{\tilde{g}}}}_{{\beta }0},\\ \partial _{\alpha }({{{\tilde{g}}}}_{00}-2)&=-2\mathop {\textrm{Re}}\nolimits (\lambda ^\gamma _\alpha {{{\tilde{g}}}}_{\gamma 0}),\\ \partial _\alpha \langle m,{\bar{m}}\rangle&=-2i A_\alpha \langle m,{\bar{m}}\rangle -2\mathop {\textrm{Re}}\nolimits \lambda ^\gamma _\alpha {\bar{{{{\tilde{g}}}}}}_{\gamma 0},\\ \partial _\alpha (g_{{\beta }\gamma }- {{{\tilde{g}}}}_{{\beta }\gamma })&=\Gamma ^\sigma _{\alpha {\beta }}(g_{\sigma \gamma }-{{{\tilde{g}}}}_{\sigma \gamma })+\Gamma ^\sigma _{\alpha \gamma }(g_{\sigma {\beta }}-{{{\tilde{g}}}}_{\sigma {\beta }})+\mathop {\textrm{Re}}\nolimits ({\bar{\lambda }}_{{\beta }\alpha }{{{\tilde{g}}}}_{\gamma 0}+{\bar{\lambda }}_{\gamma \alpha }{{{\tilde{g}}}}_{{\beta }0}). \end{aligned}$$

By ode uniqueness and the choice of the initial data, the desired properties (i) for the frame are indeed propagated spatially.

#### The Sobolev Regularity of the Frame

Here we show that our frame has the global regularity

$$\begin{aligned} \partial _x(F_\alpha , m) \in L^\infty H^{s}, \qquad \partial _t (F_\alpha , m) \in L^\infty H^{s-1}. \end{aligned}$$

As a consequence of the property (i), we directly see that $(F_\alpha ,m) \in L^\infty$. From (9.1) it then follows that $\partial _x (F_\alpha ,m) \in L^\infty$. This allows us to differentiate further in (9.1) and bound higher derivatives of the frame, up to the $H^{s}$ regularity for $\partial _x(F_\alpha ,m)$, which is imposed by $\lambda$. We can directly estimate this last norm. Precisely, by (9.1), (2.32) and Sobolev embeddings we have

$$\begin{aligned} \Vert \partial _x F_\alpha \Vert _{H^s}&\lesssim \ \Vert \Gamma F_\gamma +\lambda m\Vert _{H^s}\\&\lesssim \ \Vert \Gamma \Vert _{H^s}\Vert F_\gamma \Vert _{L^\infty \cap {\dot{H}}^s}+\Vert \lambda \Vert _{H^s}\Vert m\Vert _{L^\infty \cap {\dot{H}}^s}\\&\lesssim \ \epsilon _0 (\Vert g\Vert ^{1/2}_{L^\infty }+\Vert \partial _x F_\alpha \Vert _{H^s})+\epsilon _0(1+\Vert \partial _x m\Vert _{H^s})\\&\lesssim \ \epsilon _0(1+\Vert \partial _x F_\alpha \Vert _{H^s}+\Vert \partial _x m\Vert _{H^s}) \end{aligned}$$

and

$$\begin{aligned} \Vert \partial _\alpha m\Vert _{H^{s}}&\lesssim \ \Vert Am+\lambda F_\gamma \Vert _{H^{s}}\\&\lesssim \ \Vert A\Vert _{H^{s}}\Vert m\Vert _{L^\infty \cap {\dot{H}}^s}+\Vert \lambda \Vert _{H^s}\Vert F_\gamma \Vert _{L^\infty \cap {\dot{H}}^s}\\&\lesssim \ \epsilon _0(1+\Vert \partial _x F_\alpha \Vert _{H^s}+\Vert \partial _\alpha m\Vert _{H^{s}}). \end{aligned}$$

These imply the uniform bound

$$\begin{aligned} \Vert \partial _x F_\alpha \Vert _{H^s}+\Vert \partial _x m\Vert _{H^{s}}\lesssim \epsilon _0. \end{aligned}$$

### The Moving Manifold $\Sigma _t$

Here we propagate the full map *F* by simply integrating (2.13), i.e.

$$\begin{aligned} F(t)=F(0)+\int _0^t -\mathop {\textrm{Im}}\nolimits (\psi {\bar{m}})+V^\gamma F_\gamma \textrm{d}s. \end{aligned}$$

Then by (9.1), we have

$$\begin{aligned} \partial _\alpha F(t)=\partial _\alpha F(0)+\int _0^t -\mathop {\textrm{Im}}\nolimits (\partial ^A_{\alpha } \psi {\bar{m}}-i\lambda _{\alpha \gamma }V^{\gamma } {\bar{m}})+[\mathop {\textrm{Im}}\nolimits (\psi {\bar{\lambda }}^{\gamma }_{\alpha })+\nabla _{\alpha } V^{\gamma }]F_{\gamma } \textrm{d}s, \end{aligned}$$

which is consistent with above definition of $F_\alpha$.

### The (SMCF) Equation for *F*

Here we establish that *F* solves (1.1). Using the relation $\lambda _{\alpha {\beta }}=\partial _\alpha \partial _{\beta }F \cdot m$ we have

$$\begin{aligned} -\mathop {\textrm{Im}}\nolimits (\psi {\bar{m}})&=\ -\mathop {\textrm{Im}}\nolimits (g^{\alpha {\beta }}\partial _\alpha \partial _{\beta }F\cdot (\nu _1+i\nu _2)\ (\nu _1-i\nu _2))\\&=\ (\Delta _g F\cdot \nu _1) \nu _2-(\Delta _g F\cdot \nu _2) \nu _1\\&=\ J (\Delta _g F)^{\perp }=J{\textbf{H}}(F). \end{aligned}$$

This implies that the *F* solves (1.1).

## Data Availability

Data sharing not applicable to this article as no datasets were generated or analysed during the current study.

## Footnotes

1 However, in three and higher dimensions one could still work with $\psi$ if desired.

2 Ideally here one would like to set $\delta = 0$, but this is only possible in dimensions three and higher.
